# Discovery of 5′-Substituted 5-Fluoro-2′-deoxyuridine Monophosphate Analogs: A Novel Class of Thymidylate Synthase Inhibitors

**DOI:** 10.1021/acsptsci.2c00252

**Published:** 2023-02-23

**Authors:** Madhuri Dasari, Stephen C. Pelly, Jiafeng Geng, Hannah B. Gold, Nicole Pribut, Savita K. Sharma, Michael P. D’Erasmo, Perry W. Bartsch, Carrie Sun, Kiran Toti, Rebecca S. Arnold, John A. Petros, Lingjie Xu, Yi Jiang, Eric J. Miller, Dennis C. Liotta

**Affiliations:** † Department of Chemistry, Emory University College of Arts and Sciences, Atlanta, Georgia 30322, United States; ‡ Department of Urology, Emory University School of Medicine, Atlanta, Georgia 30322, United States; § Winship Cancer Institute, Emory University, Atlanta, Georgia 30322, United States; ∥ Junrui Biotechnology, Hangzhou, Zhejiang 310000, China; ⊥ Department of Pharmacology and Chemical Biology, Emory University School of Medicine, Atlanta, Georgia 30322, United States

**Keywords:** 5-fluorouracil, FdUMP, nucleoside, nucleotide, thymidylate synthase, anticancer

## Abstract

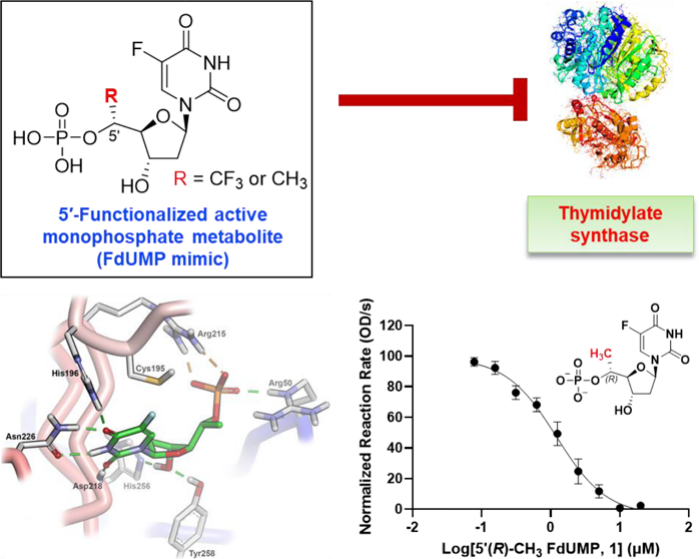

5-Fluorouracil and 5-fluorouracil-based prodrugs have been used clinically for decades to treat cancer. Their anticancer effects are most prominently ascribed to inhibition of thymidylate synthase (TS) by metabolite 5-fluoro-2′-deoxyuridine 5′-monophosphate (FdUMP). However, 5-fluorouracil and FdUMP are subject to numerous unfavorable metabolic events that can drive undesired systemic toxicity. Our previous research on antiviral nucleotides suggested that substitution at the nucleoside 5′-carbon imposes conformational restrictions on the corresponding nucleoside monophosphates, rendering them poor substrates for productive intracellular conversion to viral polymerase-inhibiting triphosphate metabolites. Accordingly, we hypothesized that 5′-substituted analogs of FdUMP, which is uniquely active at the monophosphate stage, would inhibit TS while preventing undesirable metabolism. Free energy perturbation-derived relative binding energy calculations suggested that 5′(*R*)-CH_3_ and 5′(*S*)-CF_3_ FdUMP analogs would maintain TS potency. Herein, we report our computational design strategy, synthesis of 5′-substituted FdUMP analogs, and pharmacological assessment of TS inhibitory activity.

## Introduction

**Figure 1:**
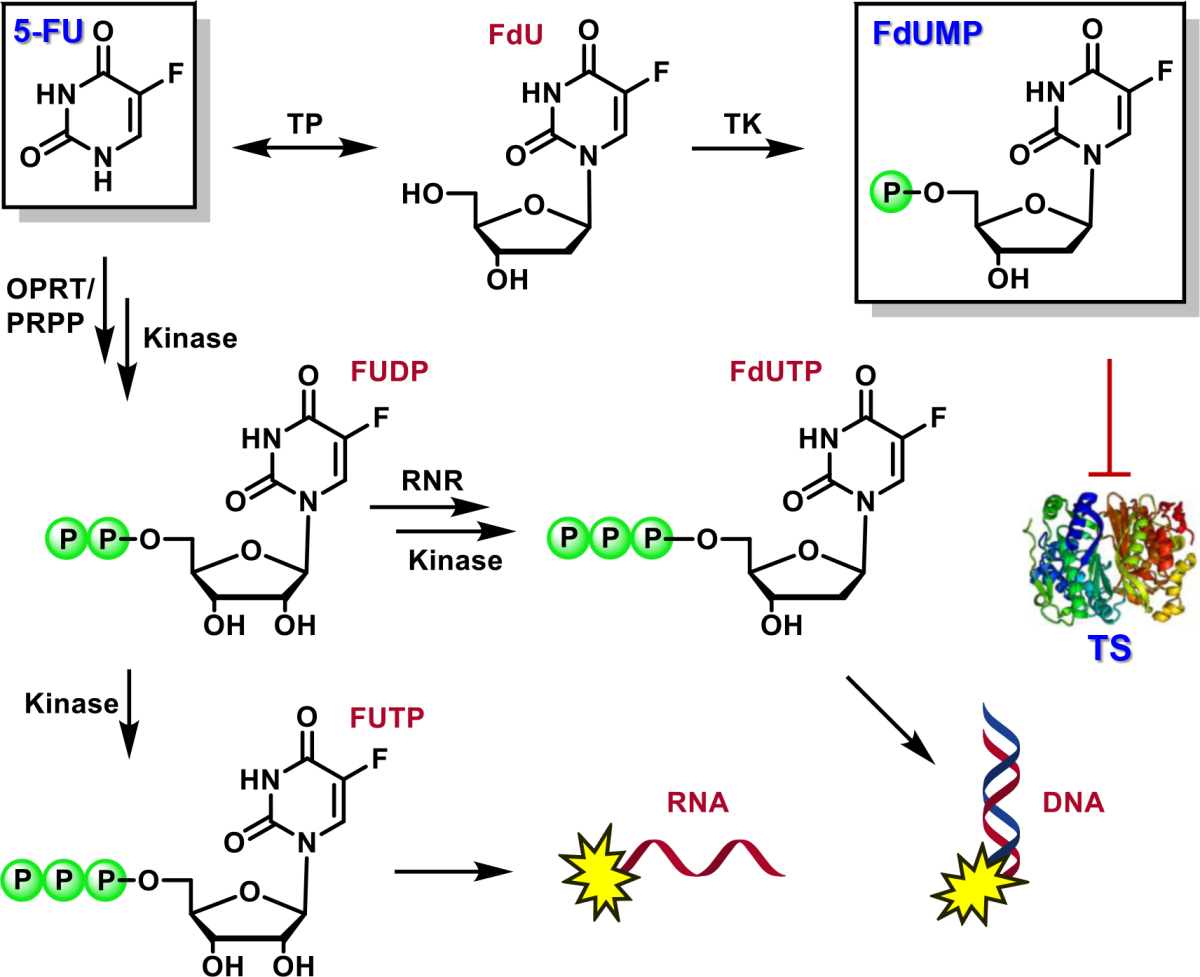
Metabolic activation of 5-fluorouracil (5-FU) to 5-fluoro-2′-deoxyuridine 5′-monophosphate (FdUMP, TS inhibitor), 5-fluoro-2′-deoxyuridine 5′-triphosphate (FdUTP, DNA polymerase substrate), and 5-fluorouridine 5′-triphosphate (FUTP, RNA polymerase substrate). TP, thymidine phosphorylase; TK, thymidine kinase; OPRT, orotate phosphoribosyl transferase; PRPP, phosphoribosyl pyrophosphate; RNR, ribonucleotide reductase; TS, thymidylate synthase (PDB ID 1HZW).

5-Fluorouracil (5-FU) is a pyrimidine-based antimetabolite approved in 1962 for treating various malignancies, including colorectal, breast, pancreatic, stomach, esophageal, and cervical cancers.^1−4^ It exerts its anticancer activity most notably through inhibition of the thymidylate synthase (TS) enzyme via its active metabolite 5-fluoro-2′-deoxyuridine 5′-monophosphate (FdUMP, Figure 1).^5−7^ Under physiological conditions, the TS enzyme plays an essential role in catalyzing the *de novo* synthesis of deoxythymidine monophosphate (dTMP) from deoxyuridine monophosphate (dUMP) in the presence of the 5,10-CH_2_-tetrahydrofolate (mTHF) cofactor and a TS active site cysteine residue.^8,9^ FdUMP covalently inhibits this catalytic cycle, leading to the depletion of dTMP levels, DNA damage, and cell death.^10−12^ 5-FU additionally generates two other cytotoxic metabolites, 5-fluoro-2′-deoxyuridine 5′-triphosphate (FdUTP) and 5-fluorouridine 5′-triphosphate (FUTP), which incorporate into growing oligonucleotides as substrates for DNA and RNA polymerases, respectively (Figure 1).^1^

**Figure 2:**
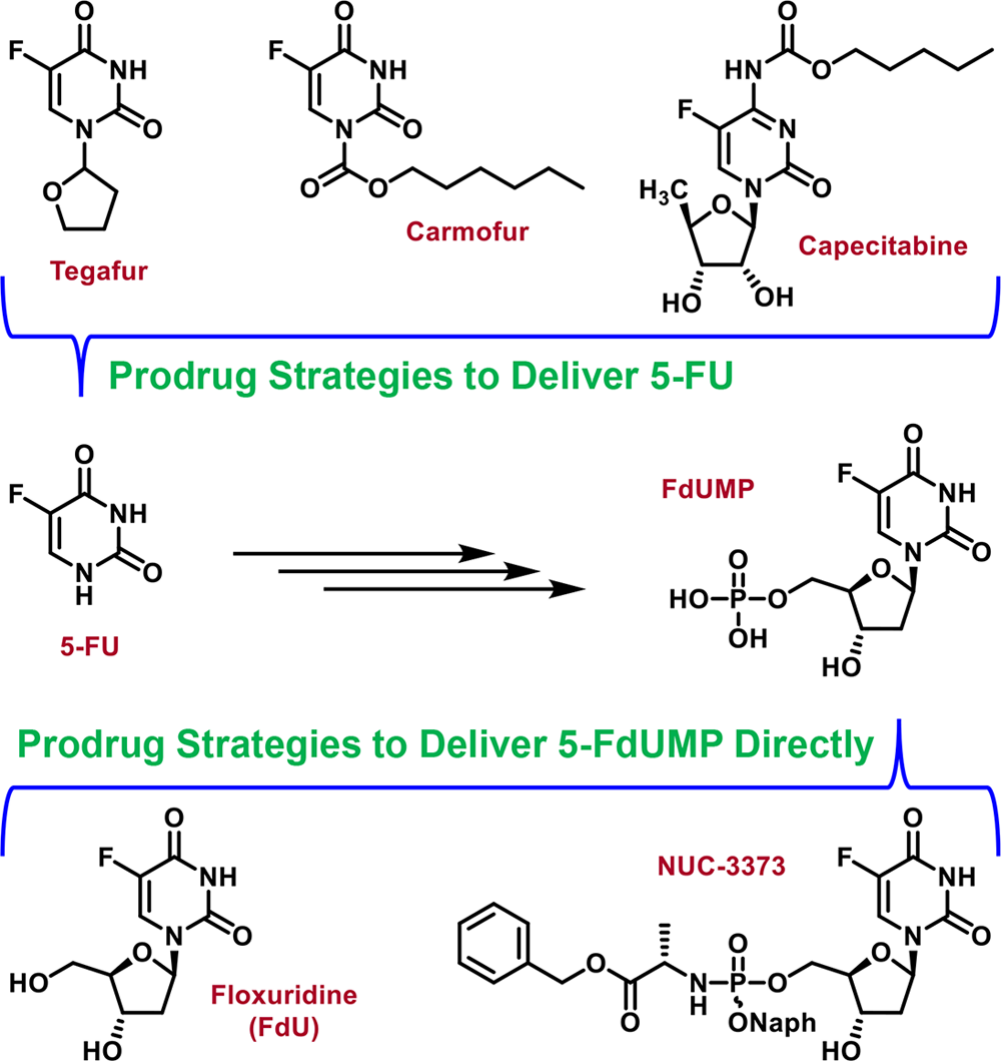
Early prodrug strategies rely on release of 5-FU to generate FdUMP, whereas other strategies (e.g., NUC-3373) generate FdUMP directly without 5-FU release.

Although 5-FU effectively treats a fraction of cancer patients with these unique mechanisms of action, its widespread clinical utility is significantly limited by catabolic degradation in the liver (up to 85%) by dihydropyrimidine dehydrogenase (DPD) as well as by poor oral bioavailability.^13,14^ To circumvent the undesired metabolic degradation and improve systemic exposure, several 5-FU prodrugs, such as capecitabine, doxifluridine, floxuridine, tegafur, and carmofur, have been developed and used in the clinic.^2,14−17^ However, the productive metabolism of these prodrugs to FdUMP proceeds via the generation of 5-FU, subsequently resulting in DPD-mediated degradation and systemic toxicity, among other undesirable consequences (Figure 2). Alternatively, the 5-FU prodrug NUC-3373 (currently in Phase 1b/2a clinical trials for the treatment of colorectal malignancies) releases the active FdUMP metabolite without generating 5-FU (Figure 2).^18−21^ While this approach offers several potential advantages over 5-FU and approved prodrugs thereof, NUC-3373 requires intravenous administration and is still responsible for the production of FdUTP and FUTP, which indiscriminately target both healthy tissue and tumor tissue.

**Figure 3:**
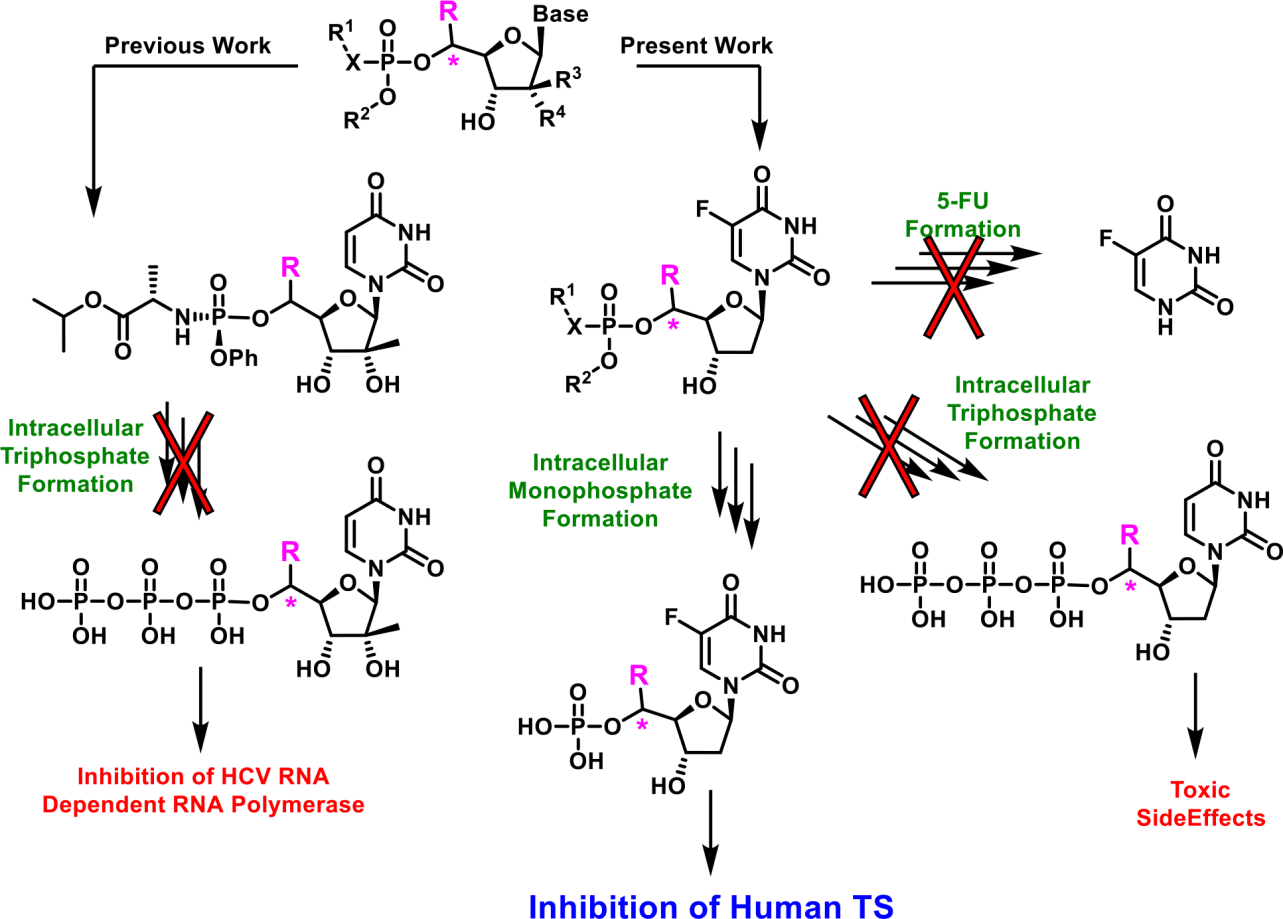
Repurposing the nucleoside/nucleotide 5′-functionalization strategy for FdUMP. R groups of interest include small substituents, such as CH_3_, CF_3_, etc.

Given the shortcomings discussed above, there is a need to develop novel analogs and/or prodrugs of FdUMP that limit the formation of promiscuous triphosphate metabolites while efficiently releasing FdUMP (or efficacious analogs thereof) and avoiding 5-FU generation. Previous results from our laboratory using cell-based hepatitis C virus (HCV) replicon assays revealed that functionalization of 2′-*C*-methyluridine at the 5′-carbon (i.e., 5′-CH_3_) significantly compromised antiviral activity of the corresponding mono- and diphosphate prodrugs (Figure 3).^22^ In this assay, the antiviral activity of nucleoside mono- and diphosphate prodrugs is dependent upon: (1) conversion of prodrug to the corresponding nucleoside monophosphate or diphosphate; (2) conversion of nucleoside monophosphate or diphosphate to the corresponding nucleoside triphosphate; and (3) substrate recognition by and concomitant inhibition of HCV RNA-dependent RNA polymerase (RdRP). Importantly, conversion of these prodrugs to the corresponding mono- and diphosphates relies on enzyme-mediated activation at positions relatively distal from the 5′ carbon. Therefore, the lack of antiviral activity of 5′-CH_3_ derivatives is much more likely to be controlled by slow conversion of mono- and diphosphate metabolites to the corresponding triphosphates and/or poor substrate recognition of these triphosphates by HCV RdRP.

While these results indicated that our 5′-functionalization strategy would clearly not be effective for nucleoside-based therapeutics requiring efficient conversion to (and target recognition of) the corresponding triphosphates, FdUMP is uniquely active at the monophosphate stage.^2^ Accordingly, we hypothesized that 5′-functionalized FdUMP analogs could maintain potent inhibition of TS and associated antitumor efficacy while reducing undesired side effects and systemic toxicity by limiting the generation of 5-FU and the corresponding nucleoside triphosphates (Figure 3). We report herein our approach to testing this hypothesis using a combination of molecular modeling, organic synthesis, and biochemical techniques.

**Figure 4:**
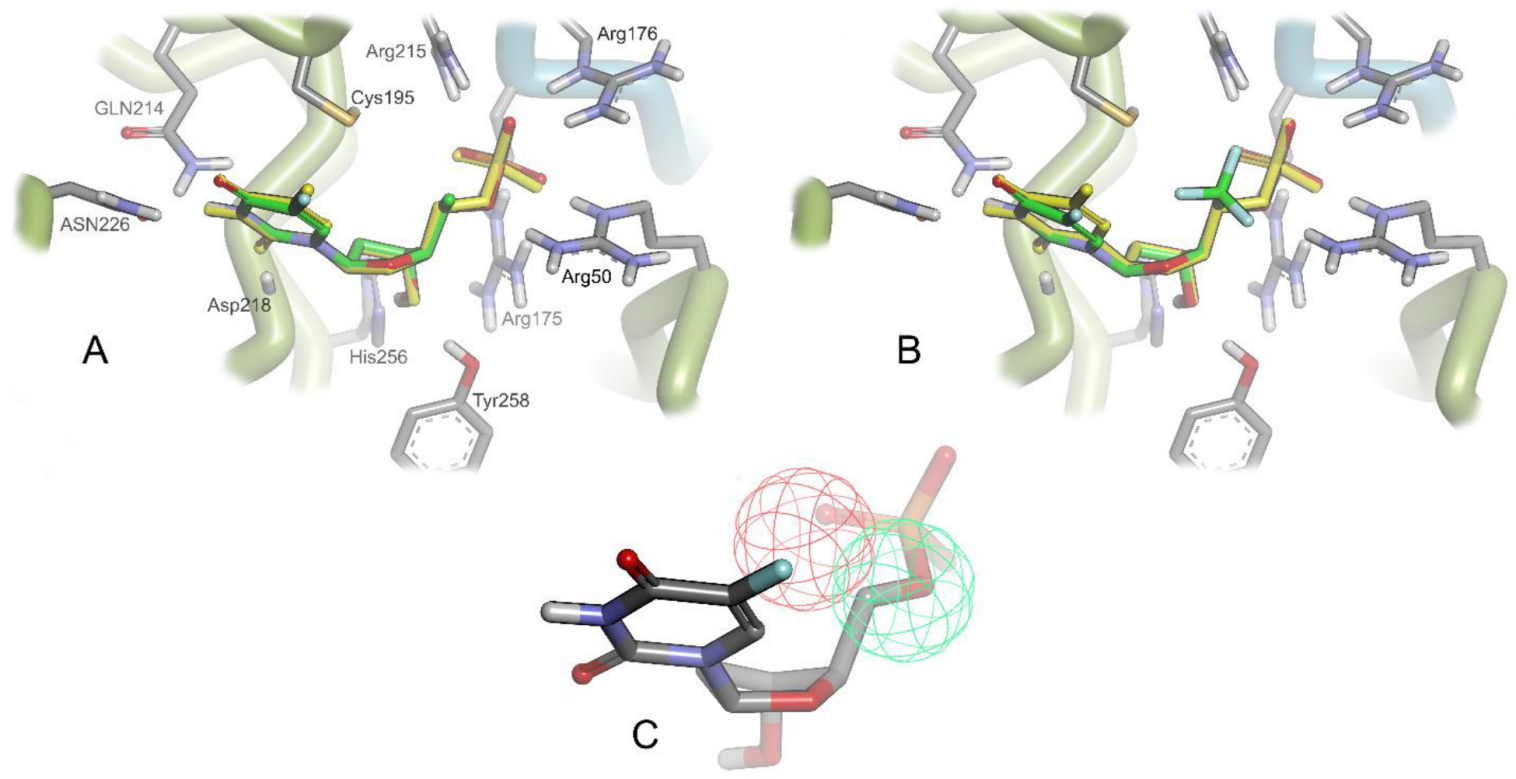
Glide-SP docking poses of (A) the 5′(*R*)-CH_3_ and (B) the 5′(*S*)-CF_3_ analogs of FdUMP in hTS (PDB 6QXG) overlaid with FdUMP (in yellow) from the cocrystal structure. (C) Graphical representation of the “substitution tolerant” zone (green sphere) and the “substitution intolerant” zone (red sphere).

Initially, molecular modeling was employed to assess whether 5′-substituted FdUMP analogs could be well accommodated within the human TS (hTS) binding pocket. Utilizing the available X-ray cocrystal structure of hTS in complex with FdUMP (PDB ID 6QXG), several FdUMP analogs featuring small 5′-substituents (Table S1) were docked (Schrödinger’s Glide-SP) into the FdUMP binding pocket. Initial binding energy calculations using Prime MM-GBSA suggested that three different substitutions, 5′(*R*)-CH_3_, 5′(*S*)-CHF_2_, and 5′(*S*)-CF_3_, would maintain TS inhibitory activity comparable to FdUMP. The predicted binding poses of FdUMP analogs featuring these substitutions also aligned very closely (Table S2) with the conformation of FdUMP cocrystallized in this pocket (PDB ID 6QXG). We further investigated relative binding free energy values using the FEP-based methodology (Desmond – D. E. Shaw research group).^23,24^ The resulting FEP-calculated binding energies suggested that two of these three substitutions, 5′(*R*)-CH_3_ and 5′(*S*)-CF_3_, would effectively inhibit TS function (Table S3). Consistent with these results are poor conformational alignments and poor FEP-predicted binding energies of FdUMP analogs featuring 5′(*S*)-CH_3_, 5′(CH_3_)_2_, and 5′(*R*)-CF_3_ motifs, relative to FdUMP and the 5′(*R*)-CH_3_- and 5′(*S*)-CF_3_-substituted analogs. As illustrated in Figure 4, a general observation from these computational experiments can be described: Small 5′-substituents occupying the green region of the TS catalytic site were much more likely to be tolerated compared to small 5′-substituents occupying the red region (Figure 4C). This is highlighted by the predicted stereochemical preference of hTS for 5′(*R*)-CH_3_ and 5′(*S*)-CF_3_ substituents (better relative binding energies, green region) over their epimeric counterparts, 5′(*S*)-CH_3_ and 5′(*R*)-CF_3_-bearing congeners (poorer relative binding energies, red region). This predicted diastereopreference can be ascribed to unfavorable intramolecular steric interactions as well as clashes with the catalytic Cys195, ultimately leading to an unsuitable binding conformation. In contrast, the green region highlights a small pocket that could nicely accommodate 5′-substituents, such as 5′(*R*)-CH_3_ and 5′(*S*)-CF_3_. It is also notable that, even with the preferred stereoconfiguration, larger substituents (e.g., ethyl) were disfavored (Table S3) relative to smaller substituents (e.g., methyl).

**Figure 5:**
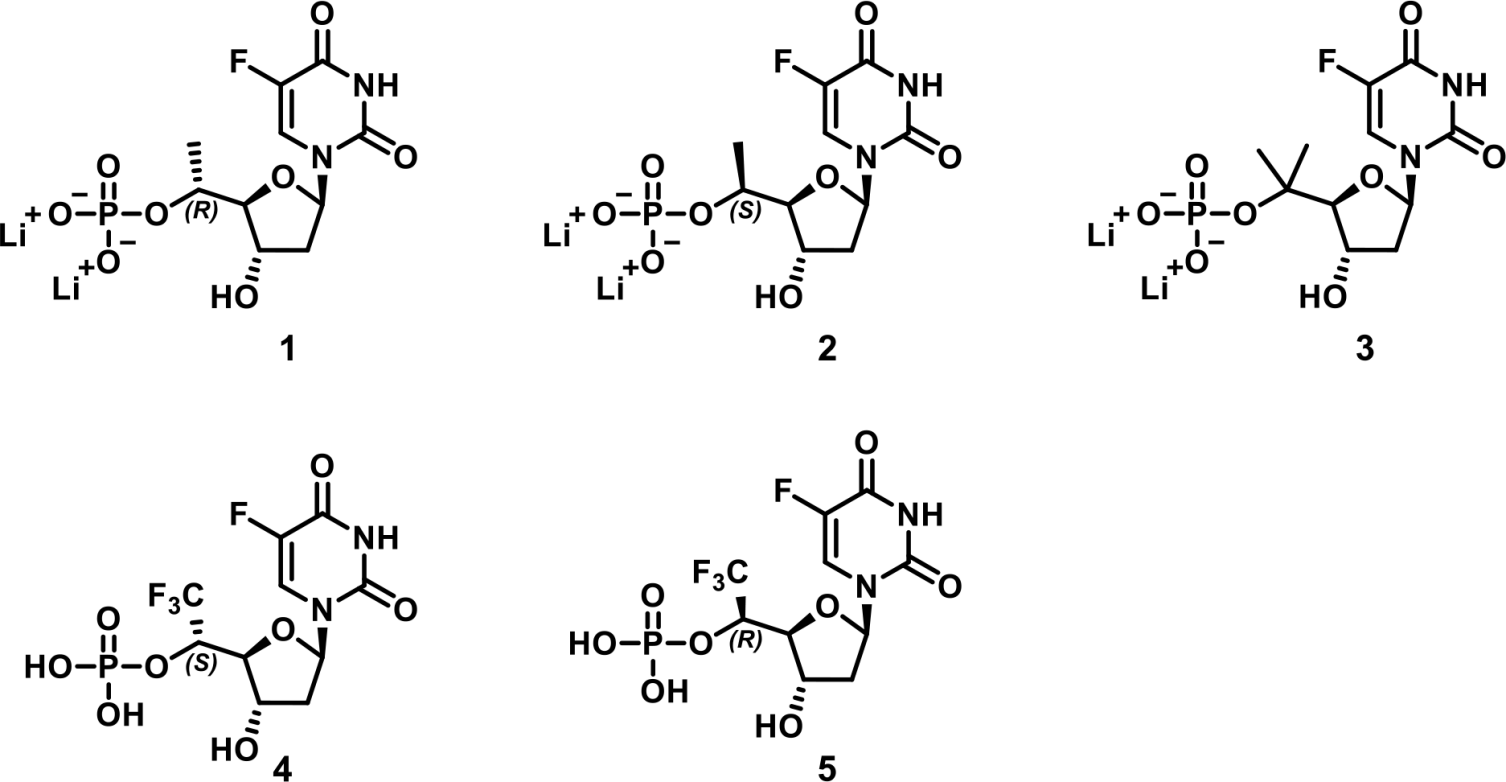
Novel 5′-substituted FdUMP analogs **1**–**5**.

**Scheme 1:**
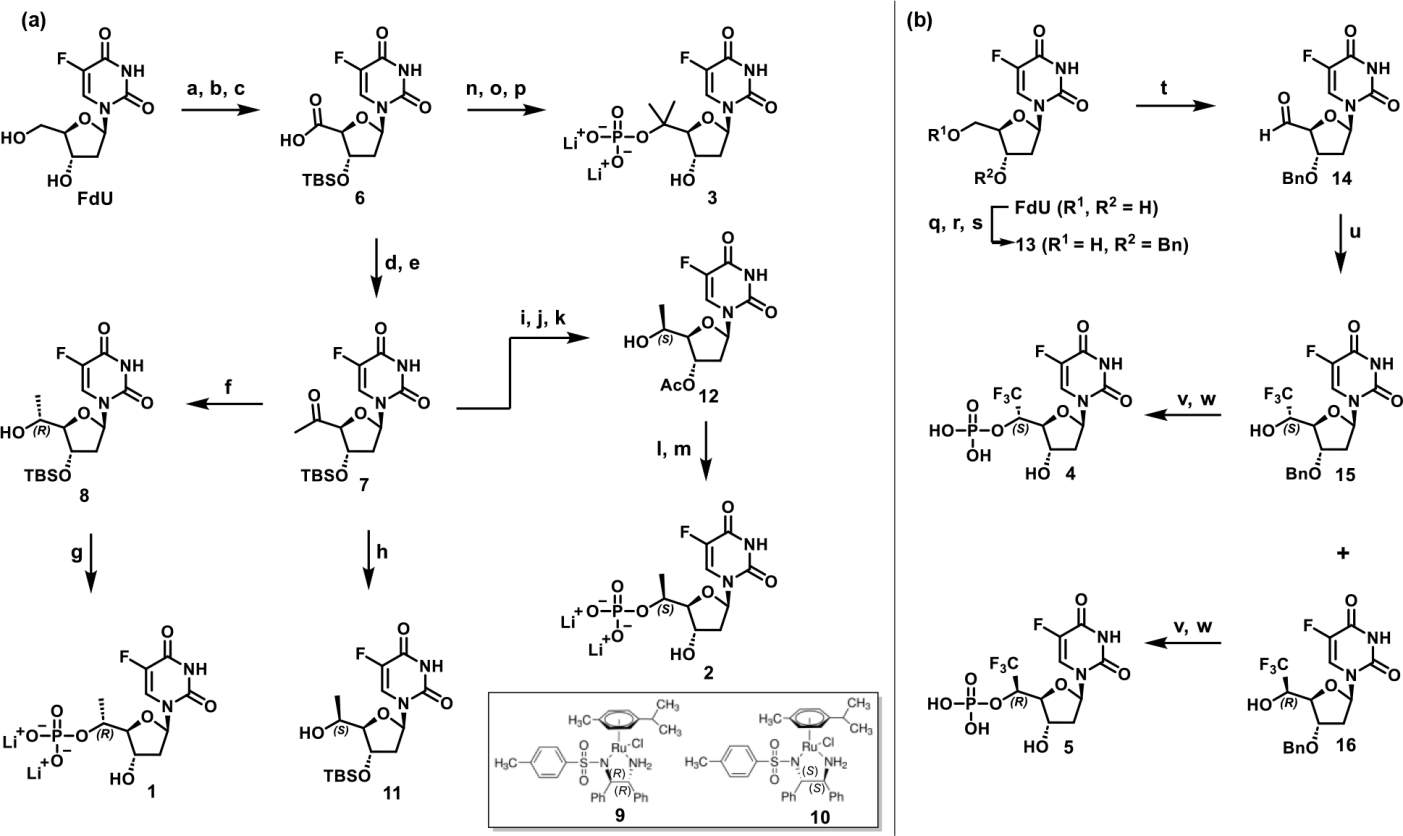
Synthesis of 5′-Functionalized FdUMP Analogs Reagents and conditions: (a) TBSCl, imidazole, DMAP, DMF, rt, 87%; (b) PPTS, MeOH, rt, 67%; (c) TEMPO, PhI(OAc)_2_, MeCN/THF/H_2_O, rt, 85%; (d) Me(OMe)NH-HCl, T3P, EtOAc/pyridine, rt (e) MeMgBr, THF, −78°C, 71%, over 2 steps; (f) **9**, HCO_2_Na, H_2_O/EtOAc, rt, 75%, 98:2 *dr*; (g) *i*, POCl_3_, pyridine, MeCN/H_2_O, 0°C to rt; *ii*, NH_4_F, H_2_O, rt; *iii*, Dowex-Li^+^, rt, 7%; (h) **10**, HCO_2_Na, H_2_O/EtOAc, rt, 83%, 7:3 *dr*; (i) TBAF, THF, 0°C; (j) Ac_2_O, DMAP, pyridine, rt, 47% over 2 steps; (k) **10**, HCO_2_Na, H_2_O/EtOAc, rt, 67%, 99:1 *dr*; (l) POCl_3_, pyridine, MeCN/H_2_O, 0°C to rt, 20%; (m) *i*, NH_3_(aq), H_2_O, rt; *ii*, Dowex-Li^+^, rt, 27% over 2 steps; (n) TMSCHN_2_, toluene, MeOH, rt, 86%; (o) MeMgBr, THF, 0°C to rt, 72%; (p) *i*, POCl_3_, trimethyl phosphate, 0°C to rt, 14%; *ii*, Dowex-Li^+^, rt, 12%; (q) TrCl, pyridine, μwave, 100°C, 78%; (r) NaH, BnBr, THF, rt, 67%; (s) 80% AcOH/H_2_O, rt, 81%; (t) DMP, DCM, rt; (u) TMSCF_3_, TBAF, THF, 0 °C to rt, then 0.5 N HCl, rt, silica gel chromatography (EtOAc/hexanes; 0–80%), 45% over 2 steps, **15** (*S*), 27%; **16** (*R*), 18%; (v) POCl_3_, pyridine, MeCN/H_2_O, 0°C to rt; (w) Pd(OH)_2_, H_2_, MeOH, rt (**4** (*S*), 12%, over 2 steps; **5** (*R*), 6%, over 2 steps).

To empirically interrogate this computationally predicted stereochemical preference, we synthesized a series of FdUMP analogs functionalized with 5′(*R*)-CH_3_, 5′(*S*)-CH_3_, 5′-*gem*-(CH_3_)_2_, 5′(*R*)-CF_3_, or 5′(*S*)-CF_3_ motifs (Figure 5) and implemented a cell-free enzymatic assay to measure TS inhibition. Synthesis of the 5′(*R/S*)-CH_3_, 5′(*R*/*S*)-CF_3_, and 5′-*gem*-(CH_3_)_2_ analogs is presented in Scheme 1. As depicted in Scheme 1a, the two 5′-CH_3_ epimers were obtained using a highly diastereoselective approach involving key synthetic intermediate **7**, which was prepared from commercially available 5-fluoro-2′-deoxyuridine (FdU). First, FdU was converted to carboxylic acid **6** in 49% yield over three steps by (1) protecting both 3′- and 5′-FdU alcohols as TBS ethers using TBSCl, imidazole, and DMAP, (2) selectively removing the 5′-OTBS moiety using PPTS, and (3) oxidizing the resulting 5′-hydroxyl group using (diacetoxyiodo)benzene and catalytic TEMPO. Intermediate **6** was next converted to the corresponding Weinreb amide using propylphosphonic anhydride (T3P) and Weinreb’s salt. The resulting crude material was treated with methylmagnesium bromide to afford the methyl ketone **7** as a key intermediate in 71% yield over two steps. Asymmetric transfer hydrogenation (ATH) of methyl ketone **7** using Noyori’s chiral catalyst, RuCl(*p*-cymene)[(*R,R*)-Ts-DPEN] (**9**), furnished the 5′(*R)*-CH_3_-functionalized alcohol **8** in 75% yield and 98:2 *dr*.^22,25,26^ The absolute configuration of the 5′(*R*) stereocenter was determined by X-ray crystal analysis (Table S5). The secondary alcohol **8** was further converted to the monophosphate **1** over three synthetic steps in 7% overall yield. Treatment of **8** with POCl_3_ and pyridine afforded the corresponding monophosphate, which was then silyl-deprotected using aqueous NH_4_F to afford the desired product as the triethylammonium salt. Counterion exchange using Dowex-Li^+^ furnished the final 5′(*R*)-CH_3_ FdUMP analog **1** as the bis-lithium salt.

To synthesize the epimeric 5′(*S*)-CH_3_ FdUMP analog (**2**, Scheme 1), key intermediate **7** was first subjected to ATH conditions using the same metal catalyst with the opposite enantiomeric ligand, RuCl(*p*-cymene)[(*S*,*S*)-Ts-DPEN] (**10**). Although this procedure generated the 5′(*S*)-CH_3_ alcohol **11** in 83% yield, the diastereoselectivity (7:3 *dr*) was suboptimal. In an attempt to enhance stereoselectivity, a small set of chiral transition metal catalysts was screened,^25^ albeit with limited success, as each of these conditions failed to substantially improve 5′(*S*)-CH_3_ diastereoselectivity, affording either predominantly the 5′-(*R*)-isomer or a diastereomeric mixture (Table S4). These results suggested that a mixture of substrate-controlled and catalyst-controlled diastereopreferential reactivity is operative. We then hypothesized that reducing the steric bulk of the 3′-OH protecting group from TBS to acetyl (Ac) would allow catalyst-controlled reactivity to predominate. Accordingly, key intermediate **7** was converted in 47% yield over two steps to its 3′-O-acetyl derivative, which was then subjected to ATH using Noyori’s chiral catalyst **10** to deliver the desired 5′(*S*)-CH_3_ alcohol **12** in 67% yield and 99:1 *dr*. The absolute configuration of the 5′(*S*) stereocenter was determined by single crystal X-ray analysis (Table S5). Alcohol **12** was then converted to the corresponding monophosphate **2** using a three-step procedure in which **12** was first treated with POCl_3_ to give the monophosphate in 20% yield. Next, removal of the 3′-O-acetyl protecting group using aqueous NH_3_, followed by counterion exchange using Dowex-Li^+^ afforded 5′(*S*)-CH_3_ FdUMP **2** as the bis-lithium salt in 27% yield over two steps. The 5′-*gem*-(CH_3_)_2_ analog **3** was synthesized in three steps from the intermediate **6**. The acid **6** was first converted to its methyl ester in 86% using TMSCHN_2_, followed by Grignard methylation using methylmagnesium bromide to give the tertiary alcohol in 72% yield. Subsequent phosphorylation conditions involving POCl_3_ additionally facilitated contaminant TBS deprotection to give the monophosphate in 14%. Counterion exchange using Dowex-Li^+^ afforded the 5′-*gem*-(CH_3_)_2_ FdUMP **3** in 12% isolated yield.

Synthesis of 5′(*R*)-CF_3_ and 5′(*S*)-CF_3_ substituted FdUMP analogs are described in Scheme 1b. First, the FdU 3′-OH group was protected as the corresponding benzyl ether **13** using a three-step protocol involving: (1) protection of the 5′-OH group as a trityl ether in 78% yield using trityl chloride in pyridine under MW irradiation at 100 °C; (2) protection of the 3′-OH as the corresponding benzyl ether in 67% yield using benzyl bromide and sodium hydride; and (3) removal of the trityl group using 80% aqueous acetic acid to afford intermediate **13** in 81% yield (42% yield over 3 steps). Next, alcohol **13** was oxidized to aldehyde **14** using DMP. The resulting crude material was then treated with Ruppert–Prakash’s reagent (TMSCF_3_) and catalytic TBAF to furnish a diastereomeric mixture of 5′-CF_3_-substituted alcohols (**15** and **16**) in 45% yield over two steps.^27−29^ Fortunately, this diastereomeric mixture could be cleanly resolved using silica gel column chromatography to deliver 5′(*S*)-CF_3_ (**15**, 27% yield) and 5′(*R*)-CF_3_ (**16**, 18% yield) derivatives, respectively. The absolute stereochemical assignment was determined using X-ray crystallography, indicating that alcohol **15** featured the 5′(*S*)-CF_3_ motif (Table S5). These intermediate alcohols were further converted to the corresponding monophosphates in two steps: (1) treatment of **15** and **16** with POCl_3_ in pyridine gave the 3′-OBn monophosphates; and (2) hydrogenolysis of the 3′-OBn groups using Pd(OH)_2_ on carbon afforded 5′(*S*)-CF_3_ FdUMP analog **4** in 12% yield and 5′(*R*)-CF_3_ FdUMP analog **5** in 6% yield over two steps.

**Figure 6:**
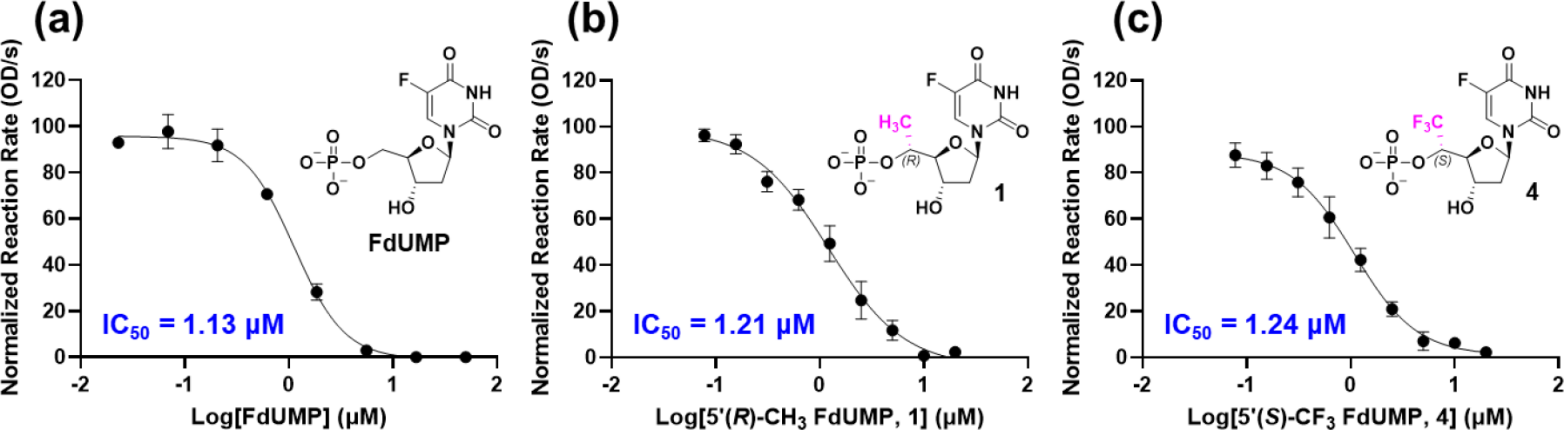
Concentration–response curves of FdUMP (a) and active 5′-functionalized analogs (b and c) against recombinant hTS-mediated conversion of 2′-deoxyuridine monophosphate (dUMP) and 5,10-methylene tetrahydrofolate (mTHF) to deoxythymidine monophosphate (dTMP) and dihydrofolate (DHF), respectively. The final concentrations of hTS, dUMP, and mTHF in the reaction systems were 0.84 μM, 50 μM, and 250 μM, respectively. The reaction rates were determined by following the change in the optical absorbance at 340 nm, which corresponded to the formation of the coproduct, DHF.

Next, we evaluated the efficacy of FdUMP, as well as all five 5′-substituted FdUMP analogs, using a cell-free hTS inhibition assay.^30−34^ This assay measures TS-catalyzed conversion of dUMP and mTHF to dTMP and dihydrofolate (DHF), the latter of which uniquely absorbs strongly at 340 nm (Figures S1 and S2). This allows reaction monitoring by measuring absorbance at 340 nm over time in the presence of decreasing concentrations of FdUMP or analog thereof (Figure S3). Concentration–response curves were constructed by plotting the normalized reaction rates (calculated using data from the first 100 s after reaction initiation) versus log[inhibitor]. Figure 6 illustrates the concentration–response curves of three of the monophosphates, including 5′(*R*)-CH_3_ FdUMP (**1**, IC_50_ = 1.21 μM, Figure 6b) and 5′(*S*)-CF_3_ FdUMP (**4**, IC_50_ = 1.24 μM, Figure 6c), both of which exhibited equimolar potency to FdUMP (IC_50_ = 1.13 μM, Figure 6a). In contrast, the 5′-epimeric isomers, 5′(*S*)-CH_3_ (**2**) and 5′(*R*)-CF_3_ (**5**), as well as the 5′-*gem*-dimethyl analog (**3**) were shown to be completely inactive even at higher concentrations (>20 μM). These results directly support the docking results (Figure 4) as well as the FEP calculations (Table S3), both of which suggested a strong stereochemical and steric preference for 5′(*R*)-CH_3_ (**1**) and 5′(*S*)-CF_3_ (**4**) FdUMP derivatives over the other three 5′-substituted congeners.

## Conclusion

In summary, the mechanism of antitumor activity of fluoropyrimidine-based therapeutics, including 5-FU, floxuridine, and capecitabine, relies heavily on inhibition of TS by the common metabolite FdUMP. However, most of the drugs and prodrugs in this class also cause significant toxicities that are driven by high systemic concentrations of 5-FU. We reported herein a novel application of our previously reported nucleoside 5′-functionalization strategy. Although this approach previously failed to deliver active anti-HCV agents, application of this concept to FdUMP, which is uniquely active at the nucleoside monophosphate stage, proved to be fruitful. As predicted by our modeling efforts and empirically corroborated by synthesizing and evaluating the TS inhibitory activity of these novel nucleoside monophosphates, a strong stereochemical preference for 5′(*R*)-CH_3_- and 5′(*S*)-CF_3_-substituted FdUMP analogs is apparent. Ongoing efforts involve mechanism of action determination and detailed metabolism experiments designed to test the hypothesis that 5′-functionalization of nucleoside and nucleotide therapeutics limits conversion to the corresponding triphosphate metabolites.

## Supporting Information Available

Supporting Information includes: The Supporting Information is available free of charge at https://pubs.acs.org/doi/10.1021/acsptsci.2c00252.

Molecular modeling protocol and associated data (Table S1, list of 5′-substitutions; Table S2, Prime MM-GBSA; Table S3, FEP); detailed synthetic schemes for compounds **1**–**5** (Schemes S1–S4); ATH catalyst screen (Table S4); small molecule X-ray crystal structures (Table S5); synthetic procedures, spectroscopic characterization data, and copies of NMR spectra for all purified compounds; *in vitro* hTS inhibition assay protocol, associated UV absorption spectra (Figures S1 and S2), and representative enzyme inhibition data (Figure S3); summary table comprising tested compounds with IC_50_ potencies and FEP relative binding energy predictions (Table S6) (PDF)

## Abbreviations

5-FU: 5-fluorouracil
FdUMP: 5-fluoro-2′-deoxyuridine 5′-monophosphate
FdUTP: 5-fluoro-2′-deoxyuridine 5′-triphosphate
FUTP: 5-fluorouridine 5′-triphosphate
FUDR: 5-fluoro-2′-deoxyuridine
TS: thymidylate synthase
hTS: human thymidylate synthase
dUMP: 2′-deoxyuridine 5′-monophosphate
dTMP: 2′-deoxythymidine 5′-monophosphate
DPD: dihydropyrimidine dehydrogenase
HCV: hepatitis C virus
RdRP: RNA-dependent RNA polymerase
MM-GBSA: molecular mechanics generalized Born surface area
FEP: free energy perturbation
FUDR: 5-fluoro-2′-deoxyuridine
TBSCl: *tert*-butyldimethylsilyl chloride
DMAP: 4-dimethylaminopyridine
PPTS: pyridinium *p*-toluenesulfonate
TEMPO: (2,2,6,6-tetramethylpiperidin-1-yl)oxy
RuCl(*p*-cymene)[(*R*,*R*)-Ts-DPEN]: [*N*-[(1*R*,2*R*)-2-(amino-κ*N*)-1,2-diphenylethyl]-4-methylbenzenesulfonamidato-κ*N*]chloro[(1,2,3,4,5,6-η)-1-methyl-4-(1-methylethyl)benzene]-ruthenium
RuCl(*p*-cymene)[(*S*,*S*)-Ts-DPEN]: [*N*-[(1*S*,2*S*)-2-(amino-κ*N*)-1,2-diphenylethyl]-4-methylbenzenesulfonamidato-κ*N*]chloro[(1,2,3,4,5,6-η)-1-methyl-4-(1-methylethyl)benzene]-ruthenium
TrCl: triphenylmethyl chloride
DMP: Dess-Martin periodinane
