# Supplementary material for: Discovery of 5′-Substituted 5-Fluoro-2′-deoxyuridine Monophosphate Analogs: A Novel Class of Thymidylate Synthase Inhibitors
Source: ACS Pharmacol Transl Sci. 2023 Feb 23;6(5):702–9. doi: 10.1021/acsptsci.2c00252 (PMC10186355; doi:10.1021/acsptsci.2c00252)
Supplement: Supplementary file 1 — pt2c00252_si_001.pdf [file pt2c00252_si_001.pdf]

Supporting Information

Discovery of 5′-Substituted 5-Fluoro-2′-Deoxyuridine Monophosphate Analogs: A Novel Class of Thymidylate Synthase Inhibitors

Madhuri Dasari[1], Stephen C. Pelly[1], Jiafeng Geng[1], Hannah B. Gold[1], Nicole Pribut[1], Savita K. Sharma[1], Michael P. D'Erasmo[1], Perry W. Bartsch[1], Carrie Sun[2], Kiran Toti[1], Rebecca S. Arnold[2,3], John A. Petros[2,3], Lingjie Xu[4], Yi Jiang[4], Eric J. Miller[3,5], and Dennis C. Liotta[1,3]**

[1]Department of Chemistry, Emory University College of Arts & Sciences, Atlanta, GA 30322, USA
[2]Department of Urology, Emory University School of Medicine, Atlanta, GA 30322, USA
[3]Winship Cancer Institute, Emory University, Atlanta, GA 30322, USA
[4]Junrui Biotechnology, Hangzhou, Zhejiang 310000, China.
[5]Department of Pharmacology & Chemical Biology, Emory University School of Medicine, Atlanta, GA 30322, USA

Table of Contents

Molecular Modeling

Protocol **pg 2-5**
Table S1 **pg 2**
Table S2 **pg 3**
Table S3 **pg 4**

Synthesis

Table S4 **pg 5**
Scheme S1 **pg 6**
Scheme S2 **pg 6**
Scheme S3 **pg 7**
Scheme S4 **pg 8**
Table S5 **pg 9**

Experimental Section

Methods and characterization **pg 10-24**
***In Vitro* HTS assay** **pg 25-28**
Figure S1 **pg 26**
Figure S2 **pg 26**
Figure S3 **pg 27**
Table S6 **pg 28**
Spectra **pg 29-83**

Molecular Modeling Protocol

Docking studies of FdUMP derivatives with one or more substitutions at the 5′ position was performed using Glide (Schrödinger Release 2022-1: Glide, Schrödinger, LLC, New York, NY, 2020). A crystal structure of human TS co-crystalized with FdUMP was used (PDB ID 6QXG, 2.08 Å, chain A and B).

Protein preparation was carried out using the Protein Preparation wizard in the Schrödinger 2022-1 suite, including adding side chains if missing. All water molecules were deleted after the preparation workflow and the FdUMP ligand in chain A was used to identify the docking binding pocket. Docking studies were carried out using Glide-SP with expanded sampling. Following this, binding energy calculations were carried out using the Prime MM-GBSA tool, with a 5 Å minimization radius. Glide-SP results are unitless, more negative values represent better docking scores. Prime MM-GBSA units are kcal/mol.

Selected FdUMP derivatives were further analyzed by free energy perturbation (FEP) methods using Desmond (D. E. Shaw Research, 2021) to compare relative binding free energy values ($\Delta\Delta G$) as compared to the co-crystalized FdUMP ligand.

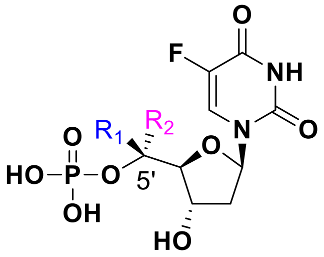

#	R_1	R_2	#	R_1	R_2
**1**	CF_3	H	**13**	CH_2OH	H
**2**	H	CF_3	**14**	H	CH_2OH
**3**	CH_3	H	**15**	CCH	H
**4**	H	CH_3	**16**	H	CCH
**5**	CHF_2	H	**17**	$CHCH_2$	H
**6**	H	CHF_2	**18**	H	$CHCH_2$
**7**	C_2H_5	H	**19**	CN	H
**8**	H	C_2H_5	**20**	H	CN
**9**	C_3H_7	H	**21**	$(CH_3)_2$	H
**10**	H	C_3H_7	**22**	H	$(CH_3)_2$
**11**	$CH(CH_3)_2$	H	**23**	Cyclopropyl	-
**12**	H	$CH(CH_3)_2$	**24**	Oxetane	-

Table S1: FdUMP analogs with various 5′-substitutions were docked in the active site of hTS.

Name (5′-moiety)	Docked pose overlaid with the FdUMP co-crystal pose	Binding Energy Prime MM-GBSA (KCal/mol)
R-CH_3	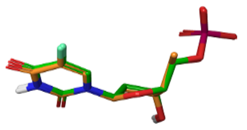	-65.10
FdUMP	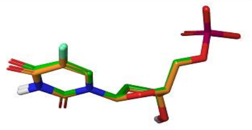	-63.35
S-CHF_2	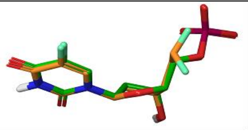	-63.11
S-CF_3	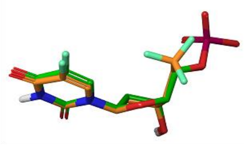	-60.57
S-CH_3	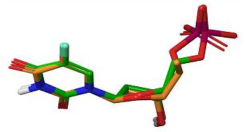	-54.82
Cyclopropyl	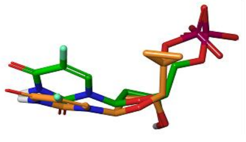	-54.64
gem-dimethyl	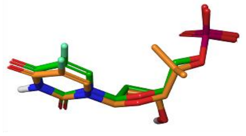	-51.86
R-CHF_2	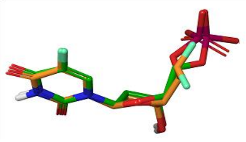	-51.18
R-CF_3	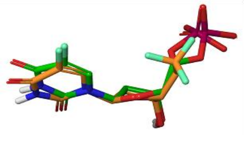	-51.03

Table S2: Prime MM-GBSA binding energy values of 5′-substituted FdUMP analogs within hTS. The first column represents various substitutions at the 5′-position of FdUMP. The second column represents superimposed poses of the co-crystal structure of FdUMP (Green) and docked poses of 5′-substituted

FdUMP (Orange). The third column represents binding energy values calculated by Prime MM-GBSA for each analog.

Structure	FEP ΔΔG (KCal/mol)	Comment
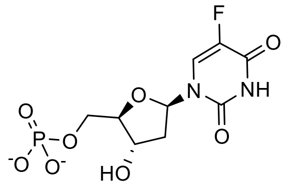	Reference	FdUMP
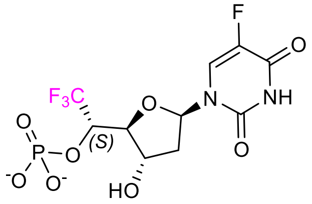	-5.24	Binds effectively
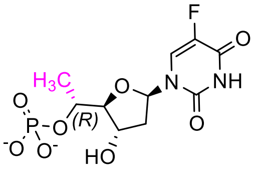	-0.62	Binds effectively
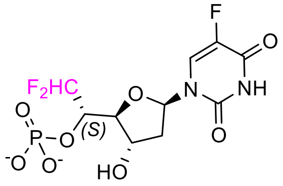	+1.19	Poorer than FdUMP
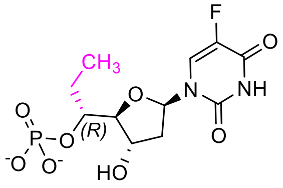	+1.49	Poorer than FdUMP
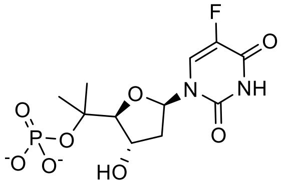	+3.60	Much poorer than FdUMP
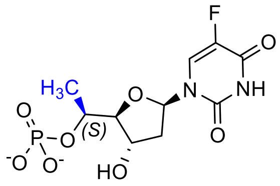	+4.46	Much poorer than FdUMP

Table S3: Relative binding energy values as calculated by FEP (Desmond – D.E. Shaw research group). The first column represents structures of FdUMP and 5′-substituted FdUMP analogs. The second column represents the relative binding energy differences calculated by FEP for FdUMP and the respectivee 5′-substituted FdUMP analogs. The third column describes the effective binding strength of 5′-substituted FdUMP analogs compared to FdUMP. Negative values imply more effective binding than the co-crystalized ligand, FdUMP.

Catalyst Screen

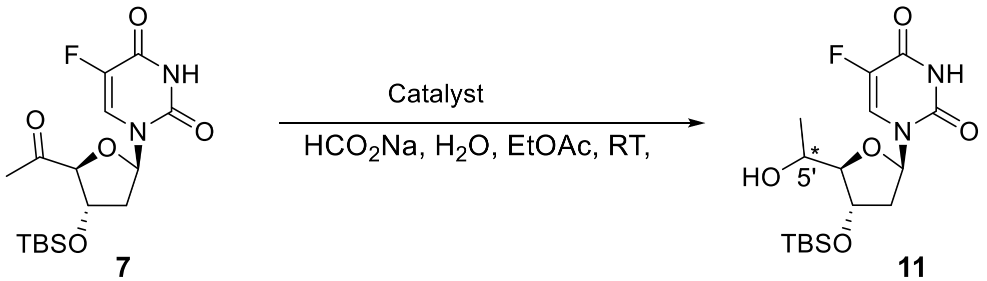

Experiment	Catalyst (Metal/Ligand)	**11** 5′ (*S/R*) dr ratio
1	$[Cp^*RhCl_2]_2$/(*S,S,S*)-CsDPEN	3:97
2	$[Cp^*RhCl_2]_2$/(*R,R*)-TsDPEN	2:98
3	$[Cp^*RhCl_2]_2$/(*S,S*)-TsDPEN	3:97
4	$[Cp^*IrCl_2]_2$/(*S,S*)-TsDPEN	44:56

Table S4: Catalyst screen for the diastereoselective conversion of **7** to **11** using different combinations of transition metal catalysts and chiral ligands.

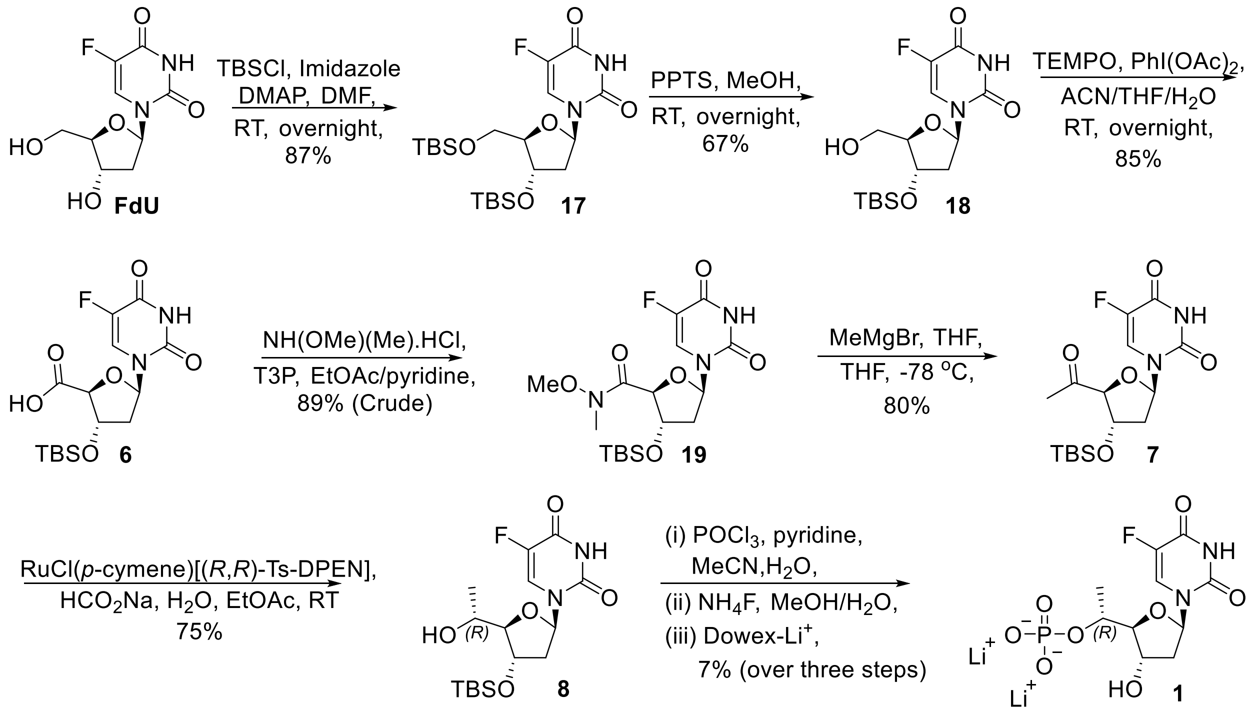

Scheme S1: Synthesis of 5′(*R*)-methyl FdUMP analog.

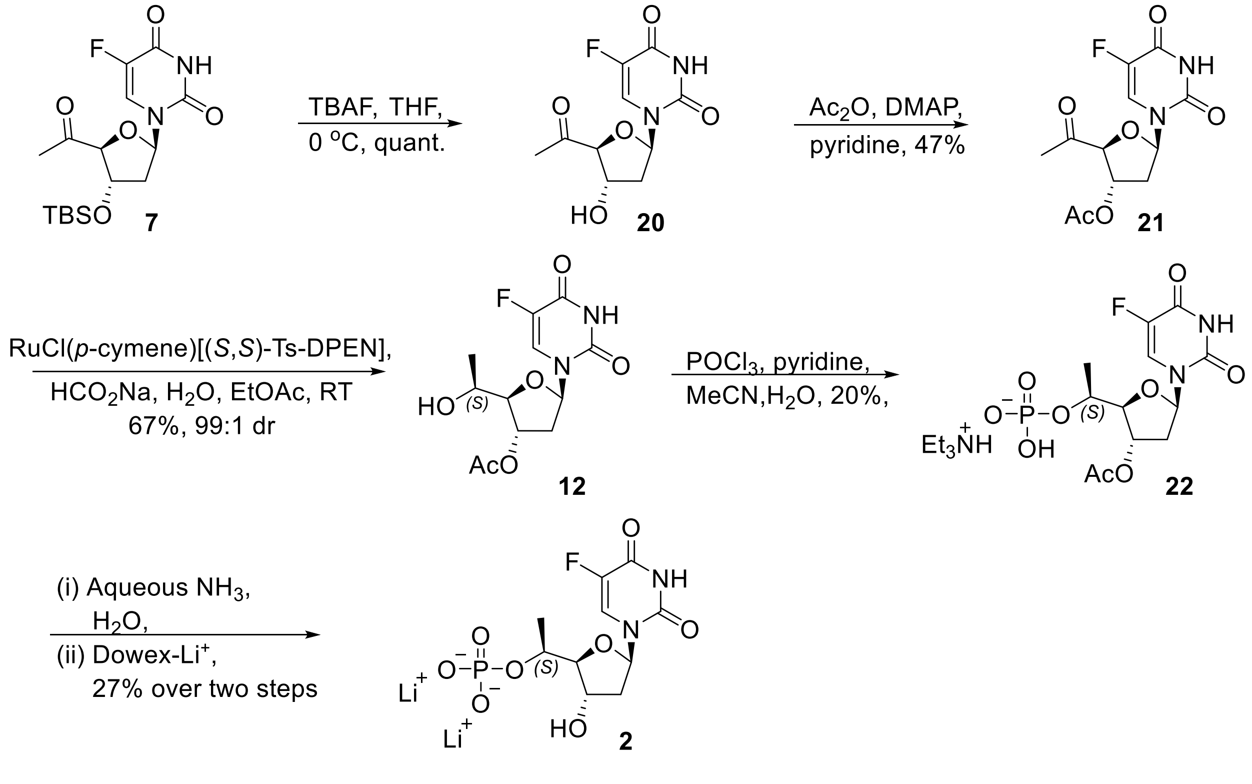

Scheme S2: Synthesis of 5′(*S*)-methyl FdUMP analog.

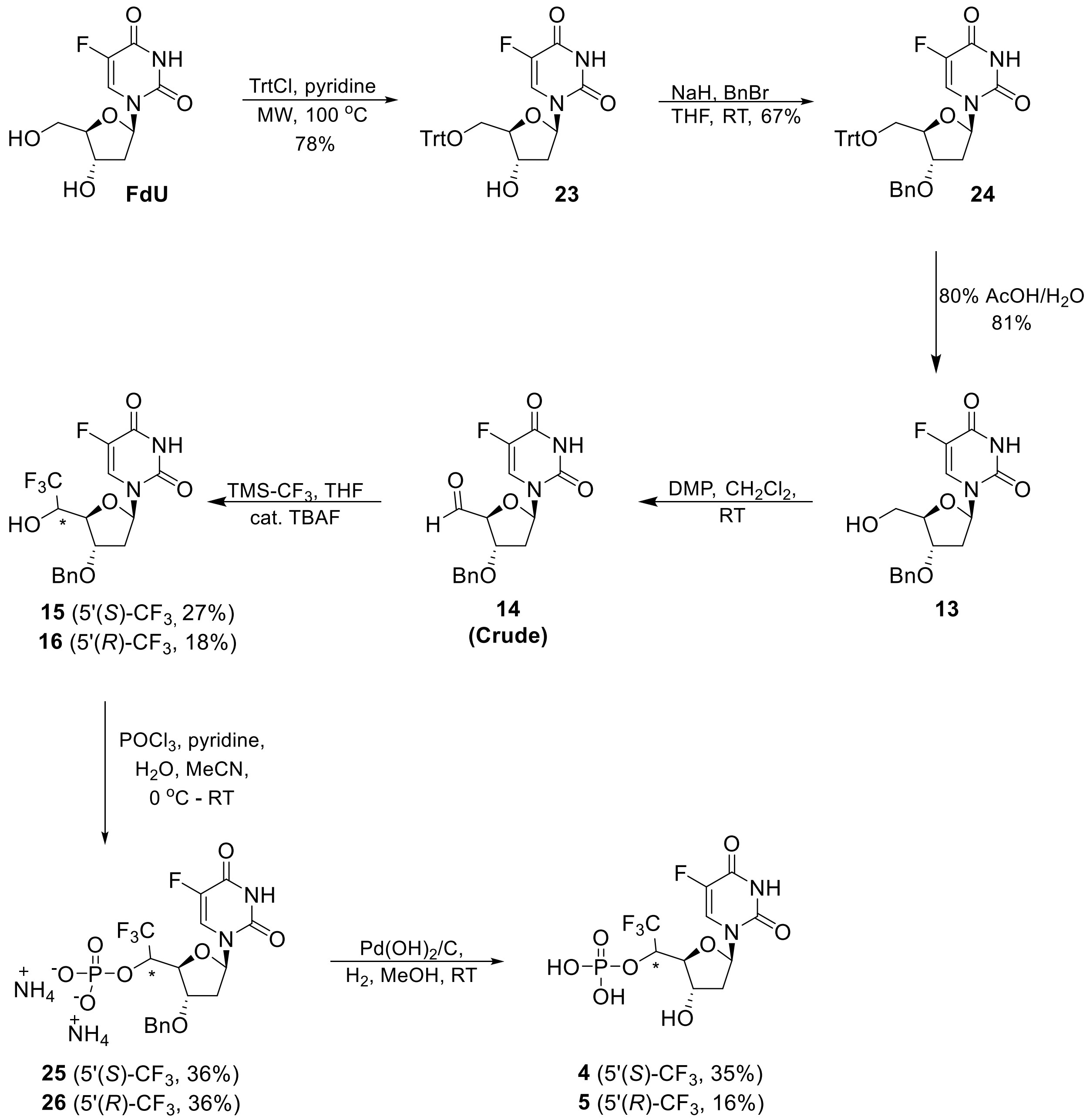

Scheme S3: Synthesis of 5′(*R*) and 5′(*S*)-trifluoromethyl FdUMP analogs.

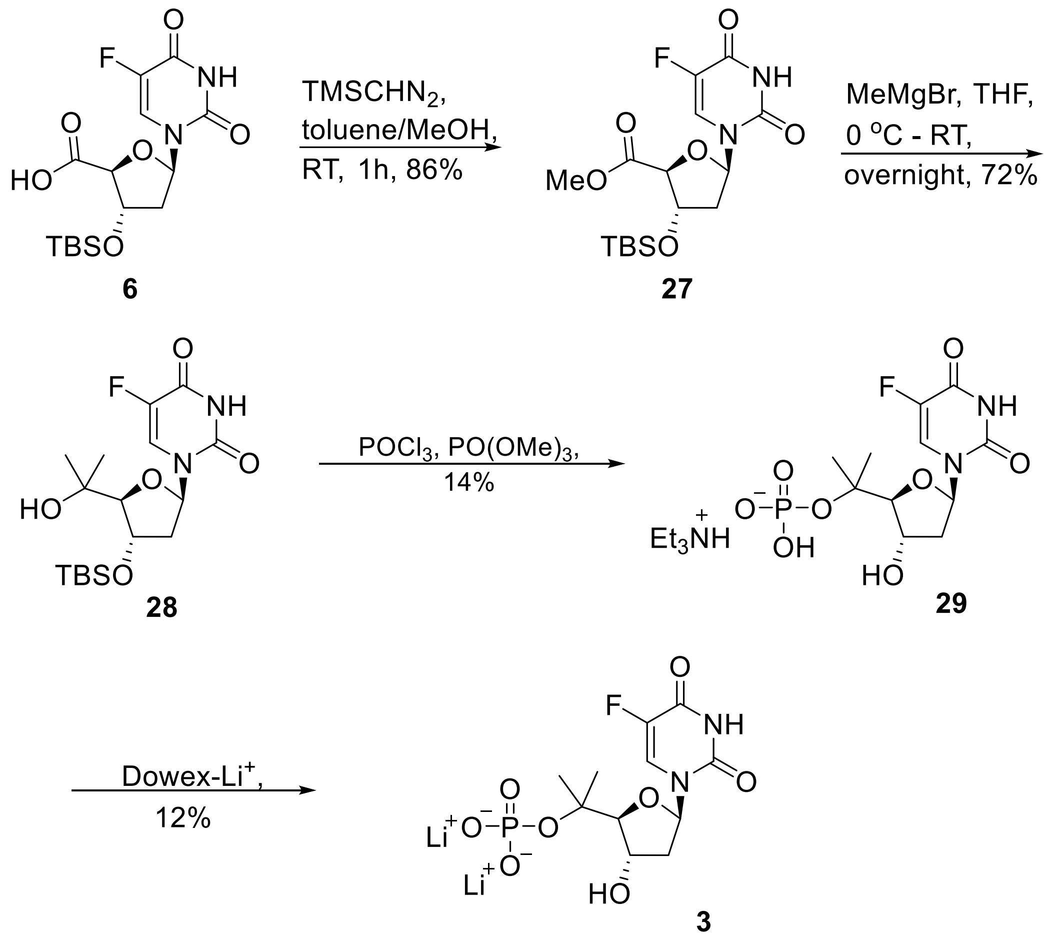

Scheme S4: Synthesis of 5′-g*em*-dimethyl FdUMP analog.

Compound	Crystal Structure
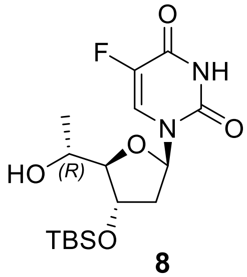 **8** (CCDC# 2194902)	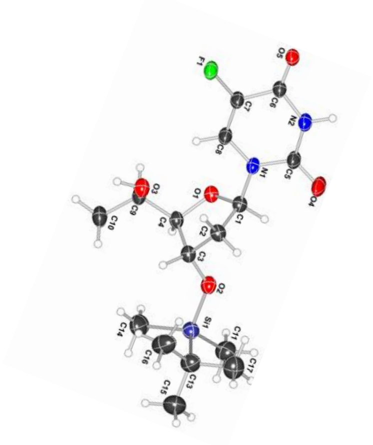
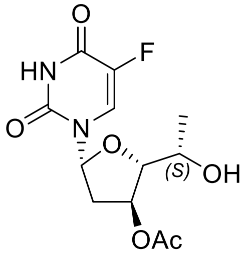 **12** (CCDC# 2194912)	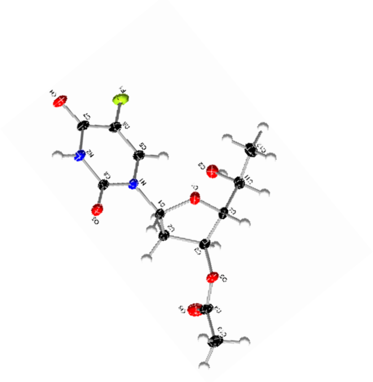
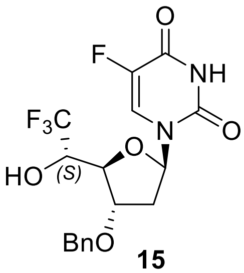 **15**	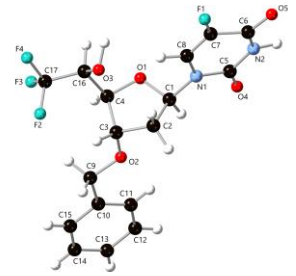

Table S5: X-ray crystal structures of 5′(*R*)-methyl, 5′(*S*)-methyl AND 5′(*S*)-trifluoromethyl FdUMP analogs

Experimental Section

Unless otherwise indicated, all reactions were conducted in oven dried or flame-dried glassware using distilled and degassed solvents under a positive pressure of dry argon with standard Schlenk techniques. General chemicals and reagents were obtained from commercial suppliers and were used without further purification. Reaction progress was monitored by either thin layer chromatography (TLC) using silica-precoated glass plates (Merck KGaA; silica gel 60 F_{254}, 0.25 mm thickness) or liquid chromatography–mass spectrometry (LC-MS) on an Agilent Technologies 6100 quadrupole instrument equipped with UV detection at 254 and 210 nm and Agilent C18 XDB eclipse column (50 mm x 4.6 mm, 3.5 µM). Automated flash column chromatography was performed using a Teledyne ISCO CombiFlash Companion system with silica gel-packed columns or RediSepRf reverse-phase C18 gold columns (Teledyne Isco). NMR spectra (^{1}H, ^{13}C, ^{19}F, and ^{31}P) were obtained using either a Varian INOVA 600 MHz spectrometer, a Varian INOVA 500 MHz spectrometer, a Varian INOVA 400 MHz spectrometer, a Varian VNMR 400 MHz spectrometer, a Bruker 400 MHz spectrometer, or a Bruker 600 MHz spectrometer. NMR samples were prepared and processed in deuterated chloroform ($CDCl_3$, residual solvent peaks: ^{1}H = 7.26 ppm, ^{13}C = 77.16 ppm) or deuterated methanol (CD_3OD, residual solvent peaks: ^{1}H = 3.31 ppm, ^{13}C = 49.0 ppm) or deuterated water (D_2O, residual solvent peak: ^{1}H = 4.79 ppm) or deuterated DMSO (DMSO, residual solvent peaks: ^{1}H = 2.50 ppm, ^{13}C = 39.52 ppm). The residual chloroform or methanol or H_2O peak in ^{1}H NMR was used as an absolute reference for ^{31}P NMR and ^{19}F NMR, unless otherwise specified. NMR data were reported to include chemical shifts (δ) reported in ppm, multiplicities indicated as s (singlet), d (doublet), t (triplet), q (quartet), p (pentet), hept (heptet), td (triplet of doublets), m (multiplet), br (broad), coupling constants (*J*) reported in Hz, and integration normalized to 1 atom (H, C, or P). High resolution mass spectrometry (HRMS) was performed by the Emory University Mass Spectrometry Center, directed by Dr. Fred Strobel. Liquid chromatography-mass spectrometry (LC-MS) was performed on an Agilent 1200 HPLC equipped with a 6120 Quadrupole mass spectrometer (ESI-API) eluting with mixtures of HPLC grade MeOH and H_2O (all spiked with 0.1% formic acid) through an analytical, reverse-phase, Agilent C18 XDB eclipse column (50 mm x 4.6 mm, 3.5 µM). LC-MS samples were prepared in aqueous solutions of MeOH. Final compound purity was assessed using NMR and LC-MS, and purity of all final compounds reported herein were determined to be ≥95% pure.

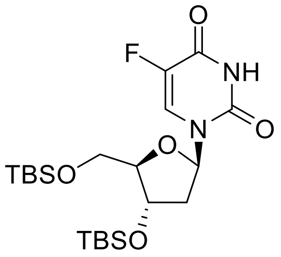

1-((2R,4S,5R)-4-((tert-butyldimethylsilyl)oxy)-5-(((tert-butyldimethylsilyl)oxy)methyl) tetrahydrofuran-2-yl)-5-fluoropyrimidine-2,4(1H,3H)-dione (17)

To a solution of FdU (5.0 g, 20.3 mmol, 1.0 eq) in anhydrous DMF (45 mL) was added imidazole (6.91 g, 101.5 mmol, 5 eq), and 4-dimethylaminopyridine (248.12 mg, 2.0 mmol, 0.1 eq). To this mixture *tert*-butyldimethylchlorosilane (7.65 g, 50.7 mmol, 2.5 eq) was added in portions and stirred at room temperature for 3 h. The reaction mixture was quenched with saturated $NaHCO_3$ solution (100 mL) and extracted with CH_2Cl_2 (x3). The organic layer was again washed with water (x2) followed by brine solution. The organic layers were dried over anhydrous Na_2SO_4, filtered, and concentrated to get a crude mixture. Purification of the crude mixture by silica gel chromatography using 0-50% EtOAc/hexanes eluted **17** in ~20-35% gradient as a white solid (8.9 g, 17.6 mmol, 87% yield). ^{1}H NMR (600 MHz, $CDCl_3$) δ 9.82 (s, 1H), 8.02 (d, *J* = 6.2 Hz, 1H), 6.28 (d, *J* = 1.7 Hz, 1H), 4.40 (dt, *J* = 6.7, 3.6 Hz, 1H), 3.94 – 3.89 (m,

2H), 3.80 – 3.65 (m, 1H), 2.31 (ddd, *J* = 13.3, 6.1, 3.9 Hz, 1H), 2.08 – 2.01 (m, 1H), 0.91 (s, 9H), 0.87 (s, 9H), 0.11 (d, *J* = 4.2 Hz, 6H), 0.06 (d, *J* = 3.6 Hz, 6H). ^{13}C NMR (151 MHz, $CDCl_3$) δ 157.2 (d, *J* = 26.6 Hz), 149.1, 140.6 (d, *J* = 236.6 Hz), 124.3 (d, *J* = 34.1 Hz), 88.1, 85.6, 71.5, 62.7, 41.9, 25.9, 25.8, 18.5, 18.0, -4.5, -4.7, -5.4, -5.5. ^{19}F NMR (565 MHz, $CDCl_3$) δ -164.2 (t, *J* = 5.6 Hz). HRMS (APCI) *m*/*z* calculated for $C_{21}H_{40}O_5N_2FSi_2$ $[M+H]^+$: 475.24543, found 475.24563.

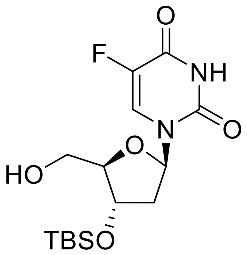

1-((2R,4S,5R)-4-((tert-butyldimethylsilyl)oxy)-5-(hydroxymethyl)tetrahydrofuran-2-yl)-5-fluoropyrimidine-2,4(1H,3H)-dione (18)

To a solution of 1-((2*R*,4*S*,5*R*)-4-((*tert*-butyldimethylsilyl)oxy)-5-(((*tert*-butyldimethylsilyl)oxy)methyl)tetrahydrofuran-2-yl)-5-fluoro pyrimidine-2,4(1*H*,3*H*)-dione (**17**, 19.0 g, 34.0 mmol, 1.0 eq) in methanol (400 mL) was added pyridinium p-toluenesulfonate (11.11 g, 44.2 mmol, 1.3 eq) and stirred at room temperature overnight with the exclusion of light. After 17 h, the reaction mixture was concentrated under reduced pressure and redissoved in EtOAc and washed with water followed by brine solution. The organic layer was dried over anhydrous Na_2SO_4, filtered, and concentrated in vacuo. Purification by silica gel flash chromatography using 10 - 100% EtOAc/hexanes eluted **18** in ~50% gradient as a white solid (8.3 g, 23.0 mmol, 67% yield). ^{1}H NMR (600 MHz, $CDCl_3$) δ 9.12 (s, 1H), 7.96 (dd, *J* = 6.4, 1.4 Hz, 1H), 6.23 (td, *J* = 6.5, 3.3 Hz, 1H), 4.66 – 4.30 (m, 1H), 4.06 – 3.83 (m, 2H), 3.80 (dt, *J* = 11.3, 2.2 Hz, 1H), 2.55 – 1.93 (m, 2H), 0.89 (s, 9H), 0.08 (s, 6H). ^{13}C NMR (151 MHz, $CDCl_3$) δ 157.0 (d, *J* = 26.8 Hz), 148.9, 140.6 (d, *J* = 236.8 Hz), 125.1 (d, *J* = 34.3 Hz), 87.7, 86.3, 71.4, 61.9, 41.3, 25.8, 18.1, -4.5, -4.7. ^{19}F NMR (565 MHz, $CDCl_3$) δ -164.6 (t, *J* = 3.9 Hz). HRMS (APCI) *m*/*z* calculated for $C_{15}H_{26}O_5N_2FSi$ $[M+H]^+$: 361.15895, found 361.15853.

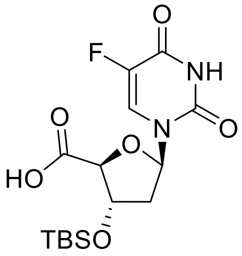

(2S,3S,5R)-3-((tert-butyldimethylsilyl)oxy)-5-(5-fluoro-2,4-dioxo-3,4-dihydropyrimidin-1(2H)-yl)tetrahydrofuran-2-carboxylic acid (6)

To a solution of 1-((2*R*,4*S*,5*R*)-4-((*tert*-butyldimethylsilyl)oxy)-5-(hydroxymethyl)tetrahydrofuran-2-yl)-5-fluoropyrimidine-2,4(1*H*,3*H*)-dione (**18**, 12.32 g, 34.1 mmol, 1.0 eq) in a mixture of MeCN (55 mL), water (55 mL), and THF (55 mL) was added iodobenzene diacetate (24.22 g, 75.1 mmol, 2.2 eq) followed by TEMPO (1.07 g, 6.8 mmol, 0.2 eq) and stirred at room temperature overnight. After 17 h, the reaction mixture was concentrated under reduced pressure to a crude solid. The solid was suspended in a mixture of CH_2Cl_2/hexanes (1:1) and filtered and rinsed thoroughly with excess solvent mixture to remove

excess reagents and byproducts. The resulting solid was freeze dried to obtain **6** as a solid (11 g, 29.3 mmol, 85%). ^{1}H NMR (400 MHz, $CDCl_3$) δ 8.64 (d, *J* = 6.8 Hz, 1H), 6.44 (ddd, *J* = 9.2, 5.2, 1.8 Hz, 1H), 4.57 (d, *J* = 4.3 Hz, 1H), 4.44 (s, 1H), 2.33 (dd, *J* = 13.2, 5.1 Hz, 1H), 1.87 (ddd, *J* = 13.4, 9.1, 4.4 Hz, 1H), 0.89 (s, 9H), 0.12 (s, 3H), 0.10 (s, 3H). ^{13}C NMR (101 MHz, $CDCl_3$) δ 173.7, 157.6 (d, *J* = 26.9 Hz), 149.2, 140.6 (d, *J* = 235.6 Hz), 125.5 (d, *J* = 35.2 Hz), 87.5, 85.7, 76.0, 39.9, 25.7, 18.0, -4.8, -4.9. ^{19}F NMR (376 MHz, $CDCl_3$) δ -164.7 (d, *J* = 6.5 Hz). HRMS (APCI) *m/z* calculated for $C_{15}H_{22}O_6N_2FSi$ $[M-H]^-$: 373.12366, found 373.12431. LC-MS (ESI) 75-95% MeOH/H_2O (0.1% HCO_2H), 3 min, 1.00 mL/min, rt = 0.79 min, *m/z* = 373 $[M-H]^-$.

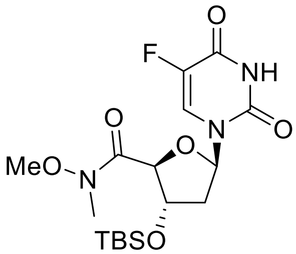

(2S,3S,5R)-3-((tert-butyldimethylsilyl)oxy)-5-(5-fluoro-2,4-dioxo-3,4-dihydropyrimidin-1(2H)-yl)-N-methoxy-N-methyltetrahydrofuran-2-carboxamide (19)

To a mixture of (2*S*,3*S*,5*R*)-3-((*tert*-butyldimethylsilyl)oxy)-5-(5-fluoro-2,4-dioxo-3,4-dihydropyrimidin-1(2*H*)-yl)tetrahydrofuran-2-carboxylic acid (**6**, 0.13 g, 0.3 mmol, 1.0 eq) and *N,O*-dimethylhydroxylamine hydrochloride (37.45 mg, 0.3 mmol, 1.15 eq) in ethyl acetate (0.9 mL) and anhydrous pyridine (0.3 mL) at 0 °C was added propylphosphoric anhydride solution (414.98 uL, 0.6 mmol, 2.0 eq) dropwise and stirred for 2 h at 0 °C. TLC analysis (5% MeOH/CH_2Cl_2) indicated complete consumption of starting material. The reaction was quenched with aqueous citric acid (1.5 mL) and extracted with EtOAc (x2). The combined organic layers were again washed with saturated $NaHCO_3$ solution followed by water and brine. The organic layer was dried over anhydrous Na_2SO_4, filtered, and concentrated under reduced pressure to get a crude mixture. The mixture was azeotropically distilled under reduced pressure by the addition of toluene (x2) followed by CH_2Cl_2 to obtain crude **19** as a solid (124 mg, 0.2 mmol, 89% crude yield), which was used in next reaction without further purification. ^{1}H NMR (600 MHz, $CDCl_3$) δ 8.95 (d, *J* = 6.8 Hz, 1H), 8.16 (s, 1H), 6.51 (ddd, *J* = 9.3, 5.1, 1.7 Hz, 1H), 4.81 (s, 1H), 4.47 (d, *J* = 4.3 Hz, 1H), 3.75 (t, *J* = 0.7 Hz, 3H), 3.25 (s, 3H), 2.28 (dd, *J* = 13.2, 5.2 Hz, 1H), 2.00 (ddd, *J* = 13.3, 9.2, 4.3 Hz, 1H), 0.91 (d, *J* = 0.6 Hz, 9H), 0.11 (s, 3H), 0.10 (s, 3H). HRMS (APCI) *m/z* calculated for $C_{17}H_{29}O_6N_3FSi$ $[M+H]^+$: 418.18042, found 418.18057. LC-MS (ESI) 95% MeOH/H_2O (0.1% HCO_2H), 3 min, 1.00 mL/min, rt = 1.5 min, *m/z* = 418 $[M+H]^+$.

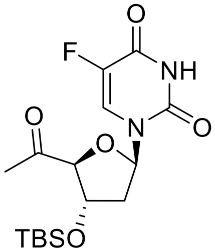

1-((2R,4S,5S)-5-acetyl-4-((tert-butyldimethylsilyl)oxy)tetrahydrofuran-2-yl)-5-fluoropyrimidine-2,4(1H,3H)-dione (7)

To a solution of (2*S*,3*S*,5*R*)-3-((*tert*-butyldimethylsilyl)oxy)-5-(5-fluoro-2,4-dioxo-3,4-dihydropyrimidin-1(2*H*)-yl)-N-methoxy-N-methyltetrahydrofuran-2-carboxamide (**19**, 3.90 g, 9.34 mmol, 1.0 eq) in

anhydrous THF (60 mL) at -22 °C (ice+NaCl) was added methylmagnesium bromide (9.34 mL, 28.0 mmol, 3.0 eq, 3.0 M in ether) dropwise and stirred for 1.5 h (during this time, the temperature rose to -18 °C). TLC analysis (50% EtOAc/hexanes) indicated complete conversion. The reaction mixture was quenched with careful addition of saturated aqueous NH_4Cl (55 mL) and allowed to warm to room temperature. The reaction mixture was extracted with EtOAc (x2). The combined organic layers were dried over anhydrous Na_2SO_4, filtered, and concentrated under reduced pressure to obtain a crude solid. The crude solid was purified by silica gel chromatography using 10-60% EtOAc/hexanes to elute **7** in ~35-42% gradient as a solid (2.8 g, 7.5 mmol, 80% yield). ^{1}H NMR (600 MHz, $CDCl_3$) δ 8.96 (s, 1H), 8.50 (d, *J* = 6.5 Hz, 1H), 6.39 (ddd, *J* = 8.2, 5.3, 1.6 Hz, 1H), 4.56 (d, *J* = 1.9 Hz, 1H), 4.50 – 4.36 (m, 1H), 2.33 (ddd, *J* = 13.4, 5.4, 2.2 Hz, 1H), 2.27 (s, 3H), 1.88 (ddd, *J* = 12.1, 8.2, 5.0 Hz, 1H), 0.93 (s, 9H), 0.15 (s, 3H), 0.14 (s, 3H). ^{13}C NMR (151 MHz, $CDCl_3$) δ 205.7, 156.9 (d, *J* = 27.1 Hz), 148.9, 140.7 (d, *J* = 237.3 Hz), 125.0 (d, *J* = 34.9 Hz), 91.1, 86.9, 73.6, 39.9, 27.5, 25.7, 18.0, -4.4, -4.7. ^{19}F NMR (565 MHz, $CDCl_3$) δ -163.7 – -163.8 (m). HRMS (APCI) *m/z* calculated for $C_{16}H_{24}O_5N_2FSi$ $[M-H]^-$: 371.1444, found 371.14466.

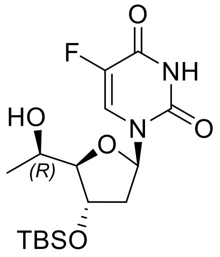

1-((2R,4S,5R)-4-((tert-butyldimethylsilyl)oxy)-5-((R)-1-hydroxyethyl)tetrahydrofuran-2-yl)-5-fluoropyrimidine-2,4(1H,3H)-dione (8)

An oven dried three neck flask was charged with 1-((2*R*,4*S*,5*S*)-5-acetyl-4-((*tert*-butyldimethylsilyl)oxy)tetrahydrofuran-2-yl)-5-fluoropyrimidine-2,4(1*H*,3*H*)-dione (**7**, 160.0 mg, 0.4 mmol, 1.0 eq) and RuCl(*p*-cymene)[(*R*,*R*)-Ts-DPEN] (**9**, 2.73 mg, 0.004 mmol, 0.01 eq) and flushed with argon. A solution of sodium formate (1.21 g, 17.8 mmol) in deoxygenated H_2O (7 mL) was added, followed by deoxygenated ethyl acetate (1.76 mL). The resulting two-phase mixture was stirred overnight at room temperature. After 17 h, the reaction mixture was diluted with CH_2Cl_2. The organic layer was separated, and the aqueous layer was again extracted with CH_2Cl_2. The combined organic layers were dried over anhydrous Na_2SO_4, filtered, and concentrated under reduced pressure to get a crude solid. Purification of the crude mixture by silica gel chromatography eluting with 0-70% EtOAc/hexanes afforded **8** as a solid (120 mg, 0.2 mmol, 75% yield, 98:2 dr). ^{1}H NMR (400 MHz, $CDCl_3$) δ 9.14 (d, *J* = 4.7 Hz, 1H), 7.94 (d, *J* = 6.4 Hz, 1H), 6.22 (ddd, *J* = 7.8, 6.1, 1.6 Hz, 1H), 4.53 (dt, *J* = 5.6, 2.8 Hz, 1H), 4.11 (qd, *J* = 6.7, 2.6 Hz, 1H), 3.81 (t, *J* = 2.6 Hz, 1H), 2.34 (s, 1H), 2.31 – 2.05 (m, 2H), 1.28 (d, *J* = 6.7 Hz, 3H), 0.89 (s, 9H), 0.09 (s, 6H). ^{13}C NMR (101 MHz, $CDCl_3$) δ 157.0 (d, *J* = 26.9 Hz), 149.0, 140.6 (d, *J* = 236.7 Hz), 125.4 (d, *J* = 34.2 Hz), 91.5, 86.4, 77.4, 70.6, 67.9, 41.4, 25.8, 20.1, 17.9, -4.3, -4.6. ^{19}F NMR (376 MHz, $CDCl_3$) δ -164.5 (ddd, *J* = 6.4, 4.7, 1.6 Hz). HRMS (APCI) *m/z* calculated for $C_{16}H_{28}O_5N_2FSi$ $[M+H]^+$: 375.1746, found 375.17445.

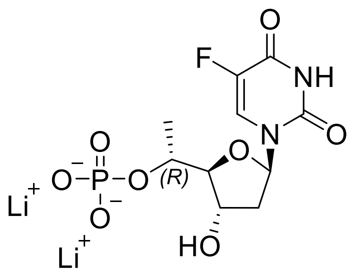

Lithium (R)-1-((2S,3S,5R)-5-(5-fluoro-2,4-dioxo-3,4-dihydropyrimidin-1(2H)-yl)-3-hydroxytetrahydrofuran-2-yl)ethyl phosphate (1)

To a solution of 1-((2*R*,4*S*,5*R*)-4-((tert-butyldimethylsilyl)oxy)-5-((*R*)-1-hydroxyethyl)tetrahydrofuran-2-yl)-5-fluoropyrimidine-2,4(1H,3H)-dione (**8**, 150 mg, 0.4 mmol, 1.0 eq) in anhydrous MeCN (0.9 mL) at 0 °C was added water (15.88 mg, 0.8 mmol, 2.2 eq) followed by pyridine (0.14 mL, 1.7 mmol, 4.4 eq). After 10 min, phosphorus oxychloride (0.17 mL, 1.7 mmol, 4.4 eq) was added dropwise and stirred overnight, allowing it to gradually warm to room temperature. After 19 h, TLC (1:1; iPrOH: iPrOH:NH_4OH:H_2O (7:2:1)) indicated trace SM and a slightly polar spot in the bottom. The reaction was cooled to 0 °C, quenched with ice cold water (0.5 mL), and stirred for 1 h. The contents were concentrated and co-concentrated with methanol (x2) to give a green-colored crude product.

TBS deprotection: The crude product was dissolved in methanol (2 mL) and transferred to a falcon tube (pH < 3). The solution was neutralized using 7N NH_3/MeOH (~600 uL). The precipitated solids were dissolved in water (2 mL), and to the resulting solution, ammonium fluoride (148.37 mg, 4.0 mmol) was added at room temperature and stirred overnight. Reaction progress was monitored by LC-MS. After 18h, The reaction mixture was concentrated and purified by RPC_{18} flash chromatography using 0-10% MeOH/H_2O to obtain the product as a 0.8 mol% Et_3N salt (15 mg, 0.034 mmol, 8% yield).

Li^+ salt: The monophosphate product with a triethylammonium counter ion was dissolved in DI water (1 mL) and slowly added to the freshly prepared Dowex-Li^+ resin and eluted with DI water (5-6 column volumes). The eluant was collected in small fractions and analyzed by nanodrop for UV absorption. The product fractions were pooled and lyophilized to obtain **1** as a white fluffy solid (10 mg, 0.028 mmol, 7.1% yield over three steps). ^{1}H NMR (600 MHz, D_2O) δ 8.07 (d, *J* = 6.2 Hz, 1H), 6.32 (ddd, *J* = 7.8, 6.0, 1.7 Hz, 1H), 4.70 – 4.56 (m, 1H), 4.42 – 4.17 (m, 1H), 3.87 (ddd, *J* = 5.4, 2.9, 1.3 Hz, 1H), 2.43 – 2.25 (m, 2H), 1.36 (d, *J* = 6.4 Hz, 3H). ^{13}C NMR (151 MHz, D_2O) δ 160.1 (d, *J* = 25.1 Hz), 150.7, 141.0 (d, *J* = 233.9 Hz), 125.8 (d, *J* = 34.2 Hz), 89.5 (d, *J* = 7.8 Hz), 85.1, 71.2 (d, *J* = 5.3 Hz), 70.6, 38.4, 17.7. ^{31}P NMR (243 MHz, D_2O) δ 1.2. HRMS (ESI) *m/z* calculated for $C_{10}H_{13}O_8N_2FP$ $[M-H]^-$: 339.0399, found 339.04038. LC-MS (ESI) 10 - 95% MeOH/H_2O (0.1% HCO_2H), 3 min, 1.00 mL/min, rt = 1.82 min, *m/z* = 339 $[M-H]^-$. LC-MS (ESI) 25-95% MeOH/H_2O (0.1% HCO_2H), 3 min, 1.00 mL/min, rt = 1.15 min, *m/z* = 339 $[M-H]^-$.

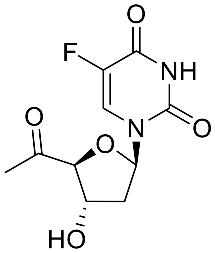

1-((2R,4S,5S)-5-acetyl-4-hydroxytetrahydrofuran-2-yl)-5-fluoropyrimidine-2,4(1H,3H)-dione (20)

To a solution of 1-((2*R*,4*S*,5*S*)-5-acetyl-4-((*tert*-butyldimethylsilyl)oxy)tetrahydrofuran-2-yl)-5-fluoropyrimidine-2,4(1*H*,3*H*)-dione (**7**, 100 mg, 0.2 mmol, 1.0 eq) in anhydrous THF (3 mL) at 0 ºC was added tetra-*n*-butylammonium fluoride (402.7 uL, 0.4 mmol, 1.5 eq) dropwise and stirred at 0 ºC for 1 h. The reaction mixture was concentrated and purified by silica gel chromatography eluting with 0-10% MeOH/CH_2Cl_2 to obtain **20** in 4-5% gradient as a solid (71 mg, 0.2 mmol, quant. yield). ^{1}H NMR (400 MHz, DMSO) δ 11.87 (s, 1H), 8.45 (d, *J* = 7.3 Hz, 1H), 6.22 (ddd, *J* = 7.8, 5.6, 1.8 Hz, 1H), 5.78 (d, *J* = 4.2 Hz, 1H), 4.54 – 4.49 (m, 1H), 4.49 – 4.44 (m, 1H), 2.20 (s, 3H), 2.13 (ddd, *J* = 13.6, 5.7, 2.3 Hz, 1H), 1.93 (ddd, *J* = 13.7, 8.4, 5.4 Hz, 1H). ^{13}C NMR (151 MHz, DMSO) δ 216.2, 166.5 (d, *J* = 26.4 Hz), 158.5, 149.4 (d, *J* = 230.1 Hz), 134.4 (d, *J* = 35.0 Hz), 100.1, 95.3, 81.0, 49.5, 36.2. ^{19}F NMR (376 MHz, DMSO) δ -167.5 (d, *J* = 7.3 Hz). HRMS (APCI) *m*/*z* calculated for $C_{10}H_{10}O_5N_2F$ $[M-H]^-$: 257.05792, found 257.05799.

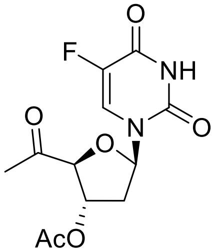

1-((2R,4S,5S)-5-acetyl-4-hydroxytetrahydrofuran-2-yl)-5-fluoropyrimidine-2,4(1H,3H)-dione (21)

To a solution of 1-((2*R*,4*S*,5*S*)-5-acetyl-4-hydroxytetrahydrofuran-2-yl)-5-fluoropyrimidine-2,4(1H,3H)-dione (**20**, 585 mg, 2.0 mmol, 1.0 eq) in anhydrous pyridine (12 mL) was added 4-dimethylaminopyridine (25.19 mg, 0.2 mmol, 0.1 eq) followed by acetic anhydride (389.79 uL, 4.1 mmol, 2.0 eq) and stirred at room temperature for 4 h. The mixture was then concentrated under reduced pressure and co-concentrated with toluene (x2) to obtain a residue. Purification by silica gel chromatography using 20-100% EtOAc/hexanes eluted **21** in 70-85% gradient as a white solid (290 mg, 0.9 mmol, 47% yield). ^{1}H NMR (600 MHz, $CDCl_3$) δ 9.26 (d, *J* = 4.7 Hz, 1H), 8.62 (d, *J* = 6.3 Hz, 1H), 6.47 (ddd, *J* = 9.5, 5.1, 1.7 Hz, 1H), 5.25 (d, *J* = 5.1 Hz, 1H), 4.72 (s, 1H), 2.54 (dd, *J* = 14.1, 5.1 Hz, 1H), 2.39 (s, 3H), 2.18 (s, 3H), 1.92 (ddd, *J* = 14.4, 9.4, 5.2 Hz, 1H). ^{13}C NMR (151 MHz, $CDCl_3$) δ 205.2, 170.7, 156.8 (d, J = 27.1 Hz), 149.2, 140.9 (d, *J* = 237.9 Hz), 124.7 (d, *J* = 35.3 Hz), 88.0, 86.6, 74.7, 35.6, 27.4, 21.0. ^{19}F NMR (565 MHz, $CDCl_3$) δ -162.8 – -162.9 (m). HRMS (ESI) *m*/*z* calculated for $C_{12}H_{14}O_6N_2F$ $[M+H]^+$: 301.08304, found 301.08264.

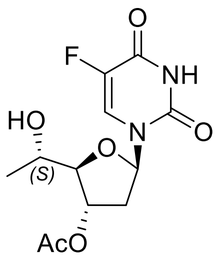

(2R,3S,5R)-5-(5-fluoro-2,4-dioxo-3,4-dihydropyrimidin-1(2H)-yl)-2-((S)-1-hydroxyethyl) tetrahydro furan-3-yl acetate (12)

An oven dried three neck flask was charged with 1-((2*R*,4*S*,5*S*)-5-acetyl-4-hydroxytetrahydrofuran-2-yl)-5-fluoropyrimidine-2,4(1H,3H)-dione (**21**, 350 mg, 1.1 mmol, 1.0 eq) and RuCl(*p*-cymene)[(*S*,*S*)-Ts-DPEN] (**10,** 7.42 mg, 0.01 mmol, 0.01 eq) and flushed with argon. A solution of sodium formate (3.30 g, 48.4 mmol, 42 eq) in deoxygenated H_2O (20 mL) was added, followed by deoxygenated ethyl acetate (4 mL). The resulting two-phase mixture was stirred overnight at room temperature. After 16 h, TLC analysis (5% MeOH/CH_2Cl_2) indicated complete conversion. The reaction mixture was diluted with EtOAc (15 mL). Organic layer was separated, and aqueous layer was reextracted with EtOAc (10 mL). The combined organic layers were dried over anhydrous Na_2SO_4, filtered, and concentrated under reduced to get a crude solid. Purification of the crude mixture by silica gel chromatography using 0-100% EtOAc/hexanes eluted **12** in 70-85% gradient as a solid (238 mg, 0.7 mmol, 67% yield). ^{1}H NMR indicated product with 99:1 dr at 5'(*S*/*R*)-carbon. ^{1}H NMR (600 MHz, $CDCl_3$) δ 8.20 (d, *J* = 6.5 Hz, 1H), 6.41 – 6.32 (m, 1H), 5.28 (d, *J* = 6.0, 1.7 Hz, 1H), 4.10 (qd, *J* = 6.5, 1.9 Hz, 1H), 3.89 (t, *J* = 1.7 Hz, 1H), 2.41 – 2.33 (m, 1H), 2.27 (ddd, *J* = 14.3, 8.7, 6.1 Hz, 1H), 2.08 (s, 3H), 1.28 (d, *J* = 6.4 Hz, 3H). ^{13}C NMR (151 MHz, $CDCl_3$+few drops MeOD) δ 170.9, 157.3 (d, *J* = 26.5 Hz), 149.1, 140.8 (d, *J* = 236.6 Hz),124.8 (d, *J* = 34.7 Hz), 88.3, 85.5, 76.3, 67.3, 37.2, 21.1, 20.2. ^{19}F NMR (565 MHz, $CDCl_3$) δ -164.2 (m). HRMS (APCI) *m*/*z* calculated for $C_{12}H_{14}O_6N_2F$ [M-H]$^-$: 301.08414, found 301.08407.

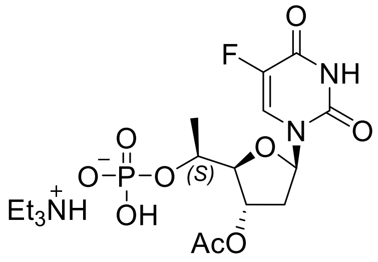

Triethylammonium (S)-1-((2S,3S,5R)-3-acetoxy-5-(5-fluoro-2,4-dioxo-3,4-dihydropyrimidin-1(2H)-yl)tetrahydrofuran-2-yl)ethyl hydrogen phosphate (22)

To a solution of [(2R,3S,5R)-5-(5-fluoro-2,4-dioxo-pyrimidin-1-yl)-2-[(1S)-1-hydroxyethyl]tetra hydrofuran-3-yl] acetate (**12**, 125 mg, 0.4 mmol, 1.0 eq) in anhydrous MeCN (1.5 mL) at 0 °C was added pyridine (0.15 mL, 1.8 mmol, 4.4 eq) followed by water (0.02 mL, 0.9 mmol, 2.2 eq). After 10 min, phosphorus oxychloride (0.17 mL, 1.8 mmol, 4.4 eq) was added dropwise and stirred at 0 °C overnight. After 17 h, the reaction was cooled to 0 °C and quenched with water (1 mL) followed by 0.5 M TEAB (10 mL) and then 1 M TEAB (10 mL) until pH >7. The contents were concentrated and co-concentrated with methanol (x2) to give a crude solid. The reaction mixture was purified by RPC_{18} flash chromatography using 0-100% MeOH/H_2O to elute the product in 25-30% gradient. The product fractions were pooled and concentrated and lyophilized to get **22** as a solid (40 mg, 0.08 mmol, 20% yield). ^{1}H NMR (400 MHz, D_2O) δ 8.27 (d, *J* = 6.2 Hz, 1H), 6.58 – 6.23 (m, 1H), 5.43 (d, *J* = 5.2 Hz, 1H), 4.52 (s, 1H), 4.19 (s, 1H), 2.69 –

2.31 (m, 2H), 2.14 (s, 3H), 1.37 (d, *J* = 5.3 Hz, 3H). ^{19}F NMR (376 MHz, D_2O) δ -164.3 (d, *J* = 6.1 Hz). HRMS (ESI) *m/z* calculated for $C_{12}H_{15}FN_2O_9P$ $[M-H]^-$: 381.05047, found 381.0503.

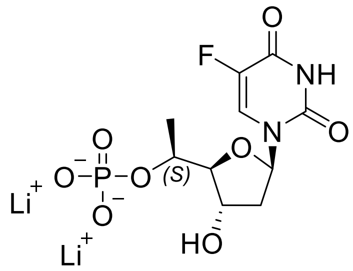

Lithium (S)-1-((2S,3S,5R)-5-(5-fluoro-2,4-dioxo-3,4-dihydropyrimidin-1(2H)-yl)-3-hydroxytetrahydrofuran-2-yl)ethyl phosphate (2)

To a solution of triethylammonium (*S*)-1-((2*S*,3*S*,5*R*)-3-acetoxy-5-(5-fluoro-2,4-dioxo-3,4-dihydropyrimidin-1(2H)-yl)tetrahydrofuran-2-yl)ethyl hydrogen phosphate (**22**, 40 mg, 0.08 mmol) in water (2 mL) was added aqueous ammonia (0.5 mL, 5.7 mmol) at room temperature and stirred overnight. Reaction progress was monitored by LC-MS (50-95% MeOH/H_2O, 3 min). After 22 h, the reaction mixture was purified by RPC_{18} flash chromatography eluting with 100% H_2O. Product fractions were pooled and lyophilized to get the product (in acid form) as a white solid (29 mg, 103% yield).

NH_4^+ salt: To the above product in methanol (2 mL) was added ammonia solution (7N in methanol) and stirred for 1 h. The mixture was concentrated and lyophilized to obtain the product with ammonium counter ion as a solid. ^1H NMR (600 MHz, D_2O) δ 8.24 (d, *J* = 6.5 Hz, 1H), 6.39 (ddd, *J* = 8.0, 6.2, 1.9 Hz, 1H), 4.60 (dt, *J* = 5.7, 2.8 Hz, 1H), 4.51 - 4.44 (m, 1H), 4.00 (q, *J* = 2.3 Hz, 1H), 2.45 – 2.25 (m, 2H), 1.39 (d, *J* = 6.5 Hz, 3H). ^{19}F NMR (376 MHz, D_2O) δ -164.9 (dd, *J* = 6.4, 1.9 Hz). ^{31}P NMR (162 MHz, D_2O) δ -0.2. ^{13}C NMR (151 MHz, D_2O) δ 159.5 (d, *J* = 26.1 Hz), 150.2, 140.8 (d, *J* = 233.1 Hz), 125.8, 125.7 (d, *J* = 34.8 Hz), 89.7 (d, *J* = 7.5 Hz), 85.2, 71.9, 71.6 (d, *J* = 5.3 Hz), 38.7, 17.8. HRMS (ESI) *m/z* calculated for $C_{10}H_{13}O_8N_2FP$ $[M-H]^-$: 339.0399, found 339.03984.

Li^+ salt: The monophosphate product with ammonium counter ions was dissolved in DI water (1 mL) and slowly added to the freshly prepared Dowex-Li^+ resin and eluted with DI water (5-6 column volumes). The eluant was collected in small fractions and analyzed by nanodrop for UV absorption. The product fractions were pooled and lyophilized to obtain **2** as a white fluffy solid (8 mg, 0.02 mmol, 27% yield). ^1H NMR (600 MHz, D_2O) δ 8.26 (d, *J* = 6.4 Hz, 1H), 6.39 (t, *J* = 7.1 Hz, 1H), 4.64 – 4.53 (m, 1H), 4.47 (t, *J* = 7.8 Hz, 1H), 4.00 (t, *J* = 2.4 Hz, 1H), 2.62 – 1.93 (m, 2H), 1.39 (d, *J* = 6.5 Hz, 3H).^{13}C NMR (151 MHz, D_2O) δ159.6 (d, *J* = 25.9 Hz),150.3, 140.9 (d, *J* = 233.1 Hz),125.9 (d, *J* = 34.6 Hz), 89.9 (d, *J* = 7.4 Hz), 85.3, 72.0,71.5 (d, *J* = 5.4 Hz), 38.7, 17.9. ^{31}P NMR (243 MHz, D_2O) δ -0.07. HRMS (ESI) *m/z* calculated for $C_{10}H_{13}O_8N_2FP$ $[M -H]^-$: 339.0399, found 339.03967. LC-MS (ESI) 25 - 95% MeOH/H_2O (0.1% HCO_2H), 3 min, 1.00 mL/min, rt = 1.43 min, *m/z* = 339 $[M-H]^-$. LC-MS (ESI) 50 - 95% MeOH/H_2O (0.1% HCO_2H), 3 min, 1.00 mL/min, rt = 1.27 min, *m/z* = 339 $[M-H]^-$.

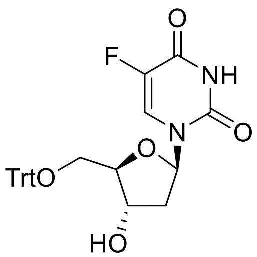

5-Fluoro-1-((2R,4S,5R)-4-hydroxy-5-((trityloxy)methyl)tetrahydrofuran-2-yl)pyrimidine-2,4(1H,3H)-dione (23)

In a microwave vial, a mixture of **FdU** (2.0 g, 8.1 mmol, 1.0 eq), trityl chloride (2.29 g, 8.2 mmol, 1.01) in anhydrous pyridine (20 mL) was subjected to MW irradiation at 100 ^{o}C for 10 mins. The mixture was then poured into 1N HCl (100 mL) and extracted with $CHCl_3$ (100 mL). The organic layer was again washed with water, followed by saturated $NaHCO_3$ solution. The organic layer was dried over anhydrous Na_2SO_4, filtered, and concentrated to give a crude solid. Purification of the crude mixture by silica gel chromatography using 20-100% EtOAc/hexanes eluted **23** in 80% gradient as a solid (2.9 g, 5.9 mmol, 73% yield). ^{1}H NMR (600 MHz, $CDCl_3$) δ 8.52 – 8.48 (m, 1H), 7.80 (d, *J* = 6.0 Hz, 1H), 7.44 – 7.40 (m, 6H), 7.35 – 7.26 (m, 6H), 7.26 (d, *J* = 3.4 Hz, 3H), 6.27 (td, *J* = 6.5, 1.6 Hz, 1H), 4.54 (dq, *J* = 6.8, 3.3 Hz, 1H), 4.04 (q, *J* = 3.4 Hz, 1H), 3.49 – 3.41 (m, 2H), 2.47 (ddd, *J* = 13.8, 6.1, 3.7 Hz, 1H), 2.26 (dt, *J* = 13.4, 6.5 Hz, 1H), 1.96 (d, *J* = 3.9 Hz, 1H). ^{19}F NMR (565 MHz, $CDCl_3$) δ -164.3. ^{13}C NMR (151 MHz, $CDCl_3$) δ 156.6 (d, *J* = 27.1 Hz), 148.5, 143.2, 140.6 (d, *J* = 238.2 Hz), 128.6, 128.2, 128.0, 127.6, 124.1 (d, *J* = 33.9 Hz), 87.9, 86.2, 85.5, 71.8, 63.3, 41.1.

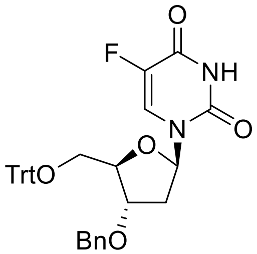

1-((2R,4S,5R)-4-(benzyloxy)-5-((trityloxy)methyl)tetrahydrofuran-2-yl)-5-fluoropyrimidine-2,4(1H,3H)-dione (24)

To a suspension of sodium hydride (614.09 mg, 15.3 mmol, 3.0 eq, 60% dispersion in mineral oil) in anhydrous THF (5 mL) was added a solution of 5-fluoro-1-[4-hydroxy-5-(trityloxymethyl) tetrahydrofuran-2-yl]pyrimidine-2,4-dione (**23**, 2.50 g, 5.12 mmol, 1.0 eq) in anhydrous THF (12 mL) dropwise at room temperature. After 1h, benzyl bromide (0.91 mL, 7.6 mmol, 1.5 eq) was added and stirred overnight. After 17 h, the reaction mixture was cooled to 0 ^{o}C and quenched with ice cold water slowly. The mixture was extracted with EtOAc (x2). The combined organic layers were washed with water followed by brine solution. The organic layer was dried over anhydrous Na_2SO_4, filtered, and concentrated to give a crude mixture. Purification by silica gel chromatography using 0-80% EtOAc/hexanes eluted **24** in 40-70% gradient as a solid. (2.0 g, 3.4 mmol, 67% yield). ^{1}H NMR (600 MHz, cdcl$_3$) δ 8.16 (d, *J* = 4.6 Hz, 1H), 7.82 (d, *J* = 6.0 Hz, 1H), 7.38-7.24 (m, 20H), 6.26 (ddd, *J* = 7.6, 5.7, 1.7 Hz, 1H), 4.58 – 4.43 (m, 2H), 4.28 (dt, *J* = 5.9, 2.8 Hz, 1H), 4.20 (q, *J* = 3.1 Hz, 1H), 3.39 (qd, *J* = 10.8, 3.2 Hz, 2H), 2.60 (ddd, *J* = 13.7, 5.9, 2.7 Hz, 1H), 2.15 (ddd, *J* = 13.6, 7.6, 6.2 Hz, 1H). ^{19}F NMR (565 MHz, $CDCl_3$) δ -164.3, -164.3. ^{13}C NMR (151 MHz, $CDCl_3$) δ 156.8 (d, *J* = 26.7 Hz), 148.7, 147.0, 143.2, 140.6 (d, *J* = 238.1 Hz), 137.3,

128.7, 128.6, 128.2, 128.1, 128.0, 127.8, 127.5, 127.3, 124.1 (d, J = 34.1 Hz), 87.8 85.7, 84.4, 78.35 71.5, 63.6, 38.4.

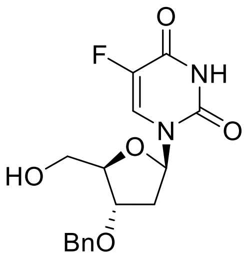

1-((2R,4S,5R)-4-(benzyloxy)-5-(hydroxymethyl)tetrahydrofuran-2-yl)-5-fluoropyrimidine-2,4(1H,3H)-dione (13)

A solution of 1-((2*R*,4*S*,5*R*)-4-(benzyloxy)-5-((trityloxy)methyl)tetrahydrofuran-2-yl)-5-fluoropyrimidine-2,4(1*H*,3i)-dione (**24,** 1.90 g, 3.2 mmol) in 80% AcOH in H_2O (15 mL) was heated at 60 ^oC for 4h. The reaction mixture was concentrated and purified by silica gel chromatography eluting with 20-100% EtOAc/hexanes to obtain **13** in ~80% gradient as a solid (0.9 g, 2.6 mmol, 81% yield). ^1H NMR (600 MHz, $CDCl_3$) δ 8.03 (d, J = 6.4 Hz, 1H), 7.35 – 7.23 (m, 5H), 6.22 (ddd, J = 7.6, 5.9, 1.6 Hz, 1H), 4.57 – 4.43 (m, 2H), 4.22 (dt, J = 5.9, 2.7 Hz, 1H), 4.13 (q, J = 2.7 Hz, 1H), 3.84 (dd, J = 11.9, 2.7 Hz, 1H), 3.69 (dd, J = 11.9, 2.6 Hz, 1H), 2.45 (ddd, J = 13.7, 6.0, 2.7 Hz, 1H), 2.10 (ddd, J = 13.7, 7.6, 6.2 Hz, 1H). ^{19}F NMR (565 MHz, $CDCl_3$) δ -165.3 (d, J = 6.6 Hz). ^{13}C NMR (151 MHz, $CDCl_3$) δ 157.6 (d, J = 26.4 Hz), 149.1, 140.5 (d, J = 235.5 Hz), 137.4, 128.6, 128.0, 127.7, 127.7, 124.9 (d, J = 34.6 Hz), 86.1, 85.4, 78.8, 71.5, 62.2, 38.0.

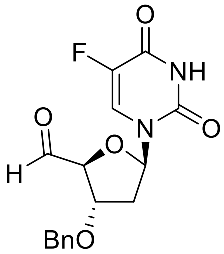

(2S,3S,5R)-3-(benzyloxy)-5-(5-fluoro-2,4-dioxo-3,4-dihydropyrimidin-1(2H)-yl)tetrahydrofuran-2-carbaldehyde (14)

To a turbid solution of 1-((2*R*,4*S*,5*R*)-4-(benzyloxy)-5-(hydroxymethyl)tetrahydrofuran-2-yl)-5-fluoropyrimidine-2,4(1*H*,3*H*)-dione (**13**, 0.65 g, 1.9 mmol, 1.0 eq) in anhydrous DCM (25 mL) was added Dess-Martin periodinane (2.45 g, 5.7 mmol, 3.0 eq) at room temperature and stirred for 3.5 h. The reaction mixture was then filtered through celite pad and rinsed thoroughly with DCM. The filtrate appeared cloudy. The filtrate was again filtered through celite pad and rinsed with DCM. The clear filtrate was concentrated and vacuum dried to obtain a crude white solid. Crude **14** was used in the next reaction without further purification (1.51 g, 1.3 mmol, 70% yield).

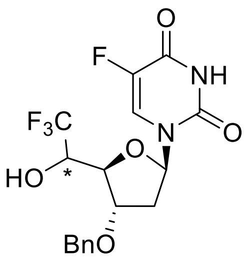

1-((2R,4S,5R)-4-(benzyloxy)-5-(2,2,2-trifluoro-1-hydroxyethyl)tetrahydrofuran-2-yl)-5-fluoropyrimidine-2,4(1H,3H)-dione (15 & 16)

To a white suspension of crude (2*S*,3*S*,5*R*)-3-(benzyloxy)-5-(5-fluoro-2,4-dioxo-3,4-dihydropyrimidin-1(2*H*)-yl)tetrahydrofuran-2-carbaldehyde (**14**, 1.50 g, 4.4 mmol, 1.0 eq, 30% pure) and (trifluoromethyl)trimethylsilane (3.31 mL, 22.4 mmol, 17 eq) in anhydrous THF (20 mL) was added tetra-*n*-butylammonium fluoride (117 mg, 0.4 mmol, 0.3 eq) at 0 ^{o}C. After addition, ice bath was removed, and the reaction was stirred at room temperature for 1h. Reaction progress was monitored by LC-MS indicated two diastereomers of the product as TMS adducts $[M-H]^-$= 475. To the reaction mixture, 0.5 N HCl (30 mL) was added and stirred overnight. LC-MS analysis indicated TMS hydrolyzed product $[M-H]^-$ = 403. The reaction mixture was extracted with EtOAc, washed with saturated $NaHCO_3$ solution followed by brine solution. The organic layer was dried over anhydrous Na_2SO_4, filtered, and concentrated to a solid. Purification by silica gel chromatography using 0-80% EtOAc/hexanes eluted the two isomers of the product consecutively in 50-60% gradient. The fractions corresponding to each isomer were concentrated separately to obtain individual isomers as solids. **15** (5′(*S*)-CF_3 isomer, fast eluting isomer): 1-[4-benzyloxy-5-[(1*S*)-2,2,2-trifluoro-1-hydroxy-ethyl]tetrahydrofuran-2-yl]-5-fluoro-pyrimidine-2,4-dione (150 mg, 0.37 mmol, 27% yield). **16** (5′(*R*)-CF_3 isomer, slow eluting isomer): 1-[4-benzyloxy-5-[(1*R*)-2,2,2-trifluoro-1-hydroxy-ethyl]tetrahydrofuran-2-yl]-5-fluoro-pyrimidine-2,4-dione (100 mg, 0.24 mmol, 18% yield). **15**:^{1}H NMR (400 MHz, $CDCl_3$) δ 7.80 (dd, *J* = 6.3 1H), 7.40 – 7.26 (m, 5H), 6.31 (ddd, *J* = 9.2, 5.6, 1.7 Hz, 1H), 4.50 (d, *J* = 2.3 Hz, 2H), 4.46 – 4.44 (m, 1H), 4.38 (t, *J* = 1.5 Hz, 1H), 4.21 (qd, *J* = 7.8, 1.9 Hz, 1H), 2.46 (dd, *J* = 13.6, 5.7, 1H), 2.16 – 2.07 (m, 1H), 0.88 – 0.77 (m, 1H). ^{19}F NMR (376 MHz, $CDCl_3$) δ -76.5 (d, *J* = 7.7 Hz), -164.6 (dd, *J* = 6.3, 1.8 Hz).). LC-MS (ESI) 50-95% MeOH/H_2O (0.1% HCO_2H), 6 min, 1.00 mL/min, rt = 2.10 min, *m/z* = 427 $[M+Na]^+$, 403.0 $[M-H]^-$. LC-MS (ESI) 75-95% MeOH/H_2O (0.1% HCO_2H), 3 min, 1.00 mL/min, rt = 1.33 min, *m/z* = 427 $[M+Na]^+$, 403.0 $[M-H]^-$. **16**: ^{1}H NMR (400 MHz, $CDCl_3$) δ 8.08 (d, *J* = 6.5 Hz, 1H), 7.39 – 7.25 (m, 5H), 6.21 (ddd, *J* = 7.7, 5.9, 1.5 Hz, 1H), 4.59 – 4.44 (m, 2H), 4.34 (dd, *J* = 2.6, 1.4 Hz, 1H), 4.22 (dt, *J* = 5.5, 2.6 Hz, 1H), 3.98 (qd, *J* = 7.3, 1.5 Hz, 1H), 2.48 – 2.39 (m, 1H), 2.17 (ddd, *J* = 13.8, 7.8, 6.0 Hz, 1H). ^{19}F NMR (376 MHz, $CDCl_3$) δ -76.3 (d, *J* = 7.5 Hz), -165.3 – -165.37 (m). ^{13}C NMR (151 MHz, $CDCl_3$) δ 157.7 (d, *J* = 26.1 Hz), 149.1, 140.5 (d, *J* = 235.4 Hz), 137.1, 128.6, 128.1, 127.7, 125.1 (d, *J* = 34.9 Hz), 123.3, 87.0, 82.2, 80.0, 71.6, 69.5 (q, *J* = 31.0, 30.4 Hz), 37.2. LC-MS (ESI) 50-95% MeOH/H_2O (0.1% HCO_2H), 6 min, 1.00 mL/min, rt = 3.55 min, *m/z* = 427 $[M+Na]^+$, 403.0 $[M-H]^-$. LC-MS (ESI) 75-95% MeOH/H_2O (0.1% HCO_2H), 6 min, 1.00 mL/min, rt = 1.17 min, *m/z* = 427 $[M+Na]^+$, 403.0 $[M-H]^-$.

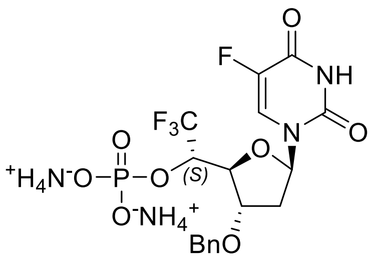

Ammonium (S)-1-((2S,3S,5R)-3-(benzyloxy)-5-(5-fluoro-2,4-dioxo-3,4-dihydropyrimidin-1(2H)-yl)tetrahydrofuran-2-yl)-2,2,2-trifluoroethyl phosphate (25)

To a solution of phosphorus oxychloride (87.25 uL, 0.93 mmol, 15 eq) in anhydrous MeCN (1.5 mL) at 0 °C was added pyridine (74.71 uL, 0.93 mmol, 15 eq) and water (8.38 uL, 0.46 mmol, 7.5 eq). After 10 min, 1-[(2*R*,4*S*,5*R*)-4-benzyloxy-5-[(1*S*)-2,2,2-trifluoro-1-hydroxy-ethyl]tetrahydrofuran-2-yl]-5-fluoro-pyrimidine-2,4-dione (**15**, 25 mg, 0.06 mmol, 1.0 eq) was added (in THF, 1 mL) and stirred at room temperature. The reaction progress was monitored by LC-MS. After 4.5 h, the reaction was cooled to 0 °C, quenched with water (15 mL), and stirred for 30 min at 0 °C. The mixture was then slowly quenched with ammonium bicarbonate solid until pH >7 and stirred at room temperature overnight. After 17 h, the mixture was concentrated in vacuo and reconstituted in methanol. The precipitated solids were filtered off and rinsed with methanol. The filtrate was concentrated to a crude solid and purified by RPC18 flash chromatography using 0-50% MeOH/H_2O to afford the product in 30% gradient. The product fractions were pooled, concentrated, and freeze dried to obtain **25** as a white solid (11.5 mg, 0.02 mmol, 35% yield). ^{1}H NMR (400 MHz, MeOD) δ 8.35 (d, *J* = 6.8 Hz, 0H), 7.60 – 7.10 (m, 2H), 6.26 (dt, *J* = 10.3, 3.6 Hz, 1H), 4.92 – 4.88 (m, 1H), 4.60 (dp, *J* = 16.7, 5.3 Hz, 1H), 4.28 (d, *J* = 3.9 Hz, 0H), 2.40 (dd, *J* = 13.5, 4.9 Hz, 0H), 2.27 – 1.90 (m, 1H). ^{19}F NMR (376 MHz, MeOD) δ -76.7 (d, *J* = 7.2 Hz), -168.5 (d, *J* = 6.5 Hz). ^{31}P NMR (162 MHz, MeOD) δ -1.2. ^{13}C NMR (151 MHz, MeOD) δ 159.4 (d, *J* = 26.5 Hz), 150.9, 142.0 (d, *J* = 233.7 Hz), 139.2, 129.3, 128.9, 128.7, 126.7 (d, *J* = 34.8 Hz), 125.2 (d, *J* = 282.3 Hz), 86.1, 83.9 (d, *J* = 3.2 Hz), 79.7, 74.3-73.9 (m), 72.4, 37.2.

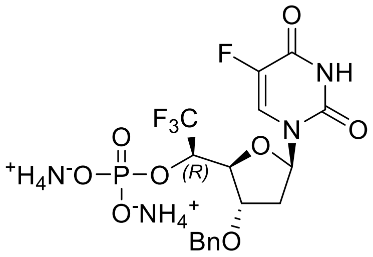

Ammonium (R)-1-((2S,3S,5R)-3-(benzyloxy)-5-(5-fluoro-2,4-dioxo-3,4-dihydropyrimidin-1(2H)-yl)tetrahydrofuran-2-yl)-2,2,2-trifluoroethyl phosphate (26)

To a solution of phosphorus oxychloride (0.05 mL, 0.54 mmol, 4.4 eq) in anhydrous MeCN (0.5 mL) at 0 °C was added pyridine (0.04 mL, 0.54 mmol, 4.4 eq) and water (0.004 mL, 0.27 mmol, 2.2 eq). After 10 min, 1-[(2*R*,4*S*,5*R*)-4-benzyloxy-5-[(1*R*)-2,2,2-trifluoro-1-hydroxy-ethyl]tetrahydrofuran-2-yl]-5-fluoro-pyrimidine-2,4-dione (**16**, 50 mg, 0.12 mmol, 1.0 eq) in anhydrous MeCN (0.7 mL) was added. After the addition, the mixture was stirred at room temperature overnight. After 15 h, LC-MS analysis didn't show any conversion. Additional pyridine (0.04 mL, 0.54 mmol, 4.4 eq) and phosphorus oxychloride (0.05 mL, 0.54 mmol, 4.4 eq) were added 0 °C and continued to stir at room temperature. Reaction progress was monitored by LC-MS. After 5 h, the reaction was cooled to 0 °C, quenched with water (15 mL), and stirred overnight at 0 °C. The mixture was then neutralized with ammonium bicarbonate solid until pH>7. The contents were concentrated in vacuo and resuspended in methanol to precipitate the inorganic salts. The

solids were removed by filtration, and the filtrate was concentrated. The process of precipitation and filtration was repeated one more time. Then, the filtrate was concentrated and purified by RPC18 flash chromatography eluting with 0-50% MeOH/H_2O. Product fractions were pooled, concentrated, and freeze fried to obtain **26** as a solid (23 mg, 0.04 mmol, 36% yield). ^{1}H NMR (400 MHz, MeOD) δ 8.41 – 8.03 (m, 1H), 7.59 – 7.09 (m, 5H), 6.55 – 6.10 (m, 1H), 4.87 (m, 1H), 4.70 (d, *J* = 5.4 Hz, 1H), 4.67 – 4.41 (m, 2H), 4.36 (m, 1H), 2.51 – 2.37 (m, 1H), 2.31 (tt, *J* = 9.4, 5.4 Hz, 1H). ^{19}F NMR (376 MHz, MeOD) δ -75.8 (d, *J* = 7.3 Hz), -168.3 (d, *J* = 6.7 Hz). ^{31}P NMR (162 MHz, MeOD) δ -1.3. ^{13}C NMR (151 MHz, MeOD) δ 159.5 (d, *J* = 26.4 Hz), 150.8, 141.9 (d, *J* = 233.4 Hz), 139.4, 129.4, 128.9, 128.8, 126.1 (d, *J* = 35.3 Hz), 125.0 (d, *J* = 279.7), 87.3, 83.9 – 83.7 (m), 82.4, 74.3-72.7 (m), 37.8.

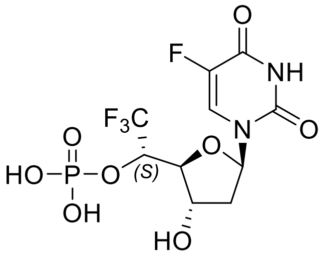

(S)-2,2,2-trifluoro-1-((2S,3S,5R)-5-(5-fluoro-2,4-dioxo-3,4-dihydropyrimidin-1(2H)-yl)-3-hydroxytetrahydrofuran-2-yl)ethyl dihydrogen phosphate (4)

A solution of ammonium (*S*)-1-((2*S*,3*S*,5*R*)-3-(benzyloxy)-5-(5-fluoro-2,4-dioxo-3,4-dihydropyrimidin-1(2*H*)-yl)tetrahydrofuran-2-yl)-2,2,2-trifluoroethyl phosphate (**25**, 14.0 mg, 0.03 mmol, 1.0 eq) in methanol (1.5 mL) was purged with argon and added palladium hydroxide on carbon (7.15 mg, 0.14 mmol, 20 wt.%, 5.3 eq) and stirred at room temperature under H_2 ballon. The reaction progress was monitored by LC-MS. After 8 h, the mixture was filtered through celite pad, and rinsed with methanol. The fiiltrate was concentrated and purified by RPC18 chromatography eluting with 100% H_2O. The product fractions were pooled and freeze dried to obtain **4** as a white fluffy solid (3.7 mg, 0.009 mmol, 35% yield). ^{1}H NMR (400 MHz, MeOD) δ 8.25 (d, *J* = 6.8 Hz, 1H), 6.29 (td, *J* = 7.9, 7.3, 1.8 Hz, 1H), 4.75 (dt, *J* = 4.9, 2.8 Hz, 2H), 4.07 (dd, *J* = 4.7, 1.9 Hz, 1H), 2.23 – 2.14 (m, 2H). ^{19}F NMR (376 MHz, MeOD) δ -76.94 (d, *J* = 7.0 Hz), -168.7 (m). ^{31}P NMR (162 MHz, MeOD) δ -1.16. LC-MS (ESI) 25-95% MeOH/H_2O (0.1% HCO_2H), 3 min, 1.00 mL/min, rt = 0.854 min, *m/z* = 393.0 $[M-H]^-$; LC-MS (ESI) 10-95% MeOH/H_2O (0.1% HCO_2H), 6 min, rt = 1.379 min, *m/z* = 393.0 $[M-H]^-$.

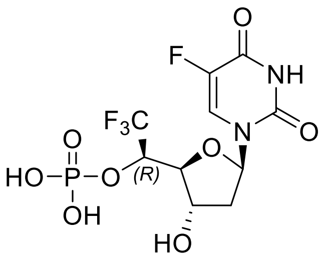

(R)-2,2,2-trifluoro-1-((2S,3S,5R)-5-(5-fluoro-2,4-dioxo-3,4-dihydropyrimidin-1(2H)-yl)-3-hydroxytetrahydrofuran-2-yl)ethyl dihydrogen phosphate (5)

A solution of ammonium (*R*)-1-((2*S*,3*S*,5*R*)-3-(benzyloxy)-5-(5-fluoro-2,4-dioxo-3,4-dihydropyrimidin-1(2*H*)-yl)tetrahydrofuran-2-yl)-2,2,2-trifluoroethyl phosphate (**26**, 15.00 mg, 0.03 mmol, 1.0 eq) in methanol (1 mL) was purged with argon and added palladium hydroxide on carbon (4.50 mg, 0.09 mmol, 20 wt.%) and stirred at room temperature under hydrogen balloon. The reaction progress was monitored by LC-MS. After 5 h, the reaction mixture was filtered through celite pad and rinsed with

methanol. The filtrate was concentrated and purified by RPC18 flash chromatography eluting with 100% H_2O. The product fractions were freeze dried to obtain **5** as a fluffy solid (1.9 mg, 0.0048 mmol, 16% yield). ^{1}H NMR (400 MHz, MeOD) δ 8.21 (d, *J* = 6.7 Hz, 1H), 6.36 (ddd, *J* = 8.1, 5.7, 1.9 Hz, 1H), 4.83 – 4.74 (m, 2H), 4.20 (s, 1H), 2.35 (ddd, *J* = 13.9, 8.5, 5.6 Hz, 1H), 2.23 (ddd, *J* = 13.6, 5.8, 2.2 Hz, 1H). ^{19}F NMR (376 MHz, MeOD) δ -75.9 (d, *J* = 7.1 Hz), -168.4 (m). ^{31}P NMR (162 MHz, MeOD) δ -1.2. LC-MS (ESI) 50-95% MeOH/H_2O (0.1% HCO_2H), 3 min, 1.00 mL/min, rt = 1.027 min, *m/z* = 393.0 $[M-H]^-$; LC-MS (ESI) 25-95% MeOH/H_2O (0.1% HCO_2H), 6 min, rt = 1.42 min, *m/z* = 393.0 $[M-H]^-$.

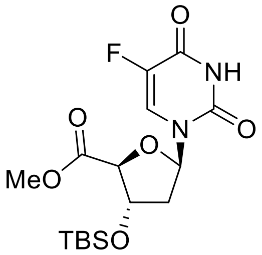

Methyl (2S,3S,5R)-3-((tert-butyldimethylsilyl)oxy)-5-(5-fluoro-2,4-dioxo-3,4-dihydropyrimidin-1(2H)-yl)tetrahydrofuran-2-carboxylate (27)

To a stirred solution of (2*S*,3*S*,5*R*)-3-((*tert*-butyldimethylsilyl)oxy)-5-(5-fluoro-2,4-dioxo-3,4-dihydropyrimidin-1(2*H*)-yl)tetrahydrofuran-2-carboxylic acid (**6**, 0.73 g, 1.9 mmol, 1.0 eq) in anhydrous methanol (4 mL) and anhydrous toluene (6 mL) was added trimethylsilyldiazomethane (3.56 mL, 3.1 mmol, 1.6 eq) dropwise at room temperature for 1 h. The reaction mixture was quenched with acetic acid and concentrated in vacuo. The crude mixture was purified by silica gel chromatography using 0-100% EtOAc/hexanes to elute **27** in ~45-50% gradient as a solid (0.65 g, 1.7 mmol, 86% yield). ^{1}H NMR (400 MHz, $CDCl_3$) δ 9.30 (d, *J* = 4.7 Hz, 1H), 8.53 (d, *J* = 6.5 Hz, 1H), 6.48 (ddd, *J* = 9.1, 5.2, 1.8 Hz, 1H), 4.50 (d, *J* = 4.4 Hz, 1H), 4.47 (s, 1H), 3.82 (s, 3H), 2.37 (ddd, *J* = 13.3, 5.2, 1.4 Hz, 1H), 1.91 (ddd, *J* = 13.5, 9.0, 4.6 Hz, 1H), 0.90 (s, 9H), 0.12 (s, 3H), 0.12 (s, 3H). ^{19}F NMR (376 MHz, $CDCl_3$) δ -163.6 (ddd, *J* = 6.5, 4.6, 1.8 Hz). ^{13}C NMR (151 MHz, $CDCl_3$) δ 171.9, 157.0 (d, *J* = 26.9 Hz), 149.1, 140.8 (d, *J* = 237.1 Hz), 125.0 (d, *J* = 35.1 Hz), 87.4, 85.5, 75.7, 52.8, 39.9, 25.7, 18.1, -4.8. HRMS (APCI) *m/z* calculated for $C_{16}H_{24}O_6N_2FSi$ $[M - H]^-$: 387.13931, found 387.13974. LC-MS (ESI): 75-95% MeOH/H_2O (0.1% HCO_2H), 3 min, rt = 2.27 min, *m/z* = 411 $[M+Na]^+$; 387 $[M-H]^-$.

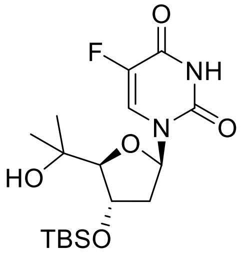

1-((2R,4S,5S)-4-((tert-butyldimethylsilyl)oxy)-5-(2-hydroxypropan-2-yl)tetrahydrofuran-2-yl)-5-fluoropyrimidine-2,4(1H,3H)-dione (28)

To a solution of methyl 3-[tert-butyl(dimethyl)silyl]oxy-5-(5-fluoro-2,4-dioxo-pyrimidin-1-yl)tetrahydrofuran-2-carboxylate (**27**, 0.84 g, 2.1 mmol, 1.0 eq) in anhydrous THF (15 mL) at 0 °C was added methylmagnesium bromide (3.60 mL, 10.8 mmol, 5. 0 eq, 3.0 M in diethylether) dropwise. After 15 min, ice bath was removed, and the reaction was allowed to warm to room temperature and stirred overnight. After total 20 h, TLC analysis (50% EtOAc/hexanes) indicated product with very close Rf to the starting material. The reaction mixture was cooled to 0 °C and quenched with saturated aqueous NH_4Cl and

extracted with EtOAc (x3). The combined organic layers were dried over anhydrous Na_2SO_4, filtered, and concentrated under reduced pressure, purified by silica gel chromatography using 20-100% EtOAc/hexanes to elute **28** in 40-50% gradient as a white solid (620 mg, 1.5 mmol, 72% yield). ^{1}H NMR (400 MHz, $CDCl_3$) δ ^{1}H NMR (400 MHz, $CDCl_3$) δ 9.16 (d, *J* = 4.9 Hz, 1H), 8.01 (d, *J* = 6.4 Hz, 1H), 6.24 (td, *J* = 6.9, 1.6 Hz, 1H), 4.51 (td, *J* = 4.8, 4.4, 2.6 Hz, 1H), 3.67 (d, *J* = 2.7 Hz, 1H), 2.25 – 2.15 (m, 2H), 2.01 (s, 1H), 1.32 (s, 3H), 1.30 (s, 3H), 0.89 (s, 9H), 0.09 (s, 6H).^{19}F NMR (376 MHz, $CDCl_3$) δ -164.4 (ddd, *J* = 6.5, 4.8, 1.6 Hz). ^{13}C NMR (151 MHz, $CDCl_3$) δ 172.6, 157.0 (d, *J* = 26.7 Hz), 149.0, 140.6 (d, *J* = 236.8 Hz), 125.5 (d, *J* = 34.5 Hz), 93.7, 86.2, 71.7, 71.5, 41.1, 27.7, 27.1, 25.8, 17.9, -4.1, -4.6. HRMS (APCI) *m/z* calculated for $C_{17}H_{28}O_5N_2FSi$ [M - H]$^-$: 387.1757, found 387.17616. LC-MS (ESI) 85% MeOH/H_2O (0.1% HCO_2H), 3 min, 1.00 mL/min, rt = 0.72 min, *m/z* = 387 [M-H]$^-$.

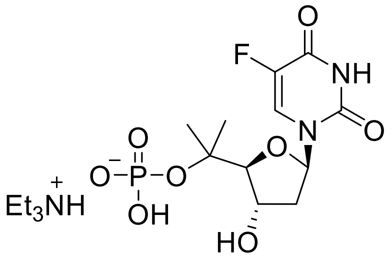

Triethylammonium 2-((2S,3S,5R)-5-(5-fluoro-2,4-dioxo-3,4-dihydropyrimidin-1(2H)-yl)-3-hydroxytetrahydrofuran-2-yl)propan-2-yl hydrogen phosphate (29)

A solution of 1-[(2R,4S,5S)-4-[tert-butyl(dimethyl)silyl]oxy-5-(1-hydroxy-1-methyl-ethyl)tetrahydrofuran-2-yl]-5-fluoro-pyrimidine-2,4-dione (**28**, 50 mg, 0.13 mmol, 1.0 eq) in trimethyl phosphate (0.5 mL, stored over activated molecular sieves overnight) was heated at 50 °C for 15 mins and then cooled to 0 °C and then added phosphorus oxychloride (0.02 mL, 0.2 mmol, 2.0 eq) dropwise and stirred overnight at 0 °C. After 4 days, the reaction was quenched with 100 mM TEAB (40 mL) at 0 °C and stirred at room temperature for 1 h. The mixture was extracted with ether (3 x 7 mL). The aqueous layer was concentrated to a crude mixture. LC-MS analysis (25-95% MeOH/H_2O, 3 min) indicated TBS deprotected product. The mixture was purified by RPC_{18} flash chromatography using 100% H_2O to elute TBS deprotected product. The product fractions were pooled and lyophilized to get a fluffy white solid. ^{1}H-NMR indicated product **29** as a triethylammonium salt (8.1 mg, 0.0178 mmol, 14% yield). ^{1}H NMR (600 MHz, D_2O) δ 8.14 (d, *J* = 6.8 Hz, 1H), 6.34 (t, *J* = 6.9 Hz, 1H), 4.87 (m, 1H), 3.95 (d, *J* = 2.7 Hz, 1H), 3.22 (q, *J* = 7.3 Hz, 6H), 2.58 (dd, *J* = 14.5, 6.4 Hz, 1H), 2.33 (dt, *J* = 14.3, 7.1 Hz, 1H), 1.35 (s, 3H), 1.32 – 1.27 (m, 12H). ^{13}C NMR (151 MHz, D_2O) δ 159.6 (d, *J* = 25.9 Hz), 150.3, 140.8 (d, *J* = 233.3 Hz), 126.0 (d, *J* = 34.4 Hz), 91.6 (d, *J* = 6.9 Hz), 84.9, 74.0, 71.1, 46.7, 38.1, 25.4, 25.0, 8.2. ^{31}P NMR (243 MHz, D_2O) δ -0.03. ^{19}F NMR (376 MHz, D_2O) δ -165.5 (d, *J* = 6.3 Hz). HRMS [ESI] m/z [M- H]$^-$ calculated for $C_{11}H_{15}O_8N_2FP$ 353.05555; found: 353.05539.

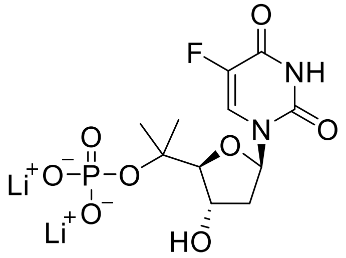

Lithium 2-((2S,3S,5R)-5-(5-fluoro-2,4-dioxo-3,4-dihydropyrimidin-1(2H)-yl)-3-hydroxytetrahydrofuran-2-yl)propan-2-yl phosphate (3)

A solution of triethylammonium salt of **29** in DI water (1 mL) was slowly added to the freshly prepared Dowex-H^+ resin and eluted with DI water (5-6 column volumes). The eluant was collected in small fractions and analyzed by nanodrop for UV absorption. The product fractions were pooled and lyophilized to obtain **3** as a white fluffy solid (5.8 mg, 0.01 mmol, 12% yield). ^{1}H NMR (600 MHz, D_2O) δ 8.14 (d, *J* = 6.4 Hz, 1H), 6.34 (t, *J* = 7.1 Hz, 1H), 4.84 (t, *J* = 3.5 Hz, 1H), 3.93 (d, *J* = 3.2 Hz, 1H), 2.58 (ddd, *J* = 14.2, 6.2, 2.9 Hz, 1H), 2.33 (dt, *J* = 14.4, 7.3 Hz, 1H), 1.35 (s, 3H), 1.31 (s, 3H). ^{13}C NMR (151 MHz, D_2O) δ 159.8 (d, *J* = 25.6 Hz), 150.5, 140.8 (d, *J* = 233.3 Hz), 126.0 (d, *J* = 34.4 Hz), 91.6 (d, *J* = 7.3 Hz), 84.9, 73.5 (d, *J* = 4.8 Hz), 38.1, 71.1, 38.1 (d, *J* = 2.2 Hz), 25.3, 25.0. ^{31}P NMR (243 MHz, D_2O) δ 0.95. HRMS [ESI] m/z $[M- H]^-$ calculated for $C_{11}H_{15}O_8N_2FP$ 353.05555; found: 353.05584. LC-MS (ESI) 25 - 95% MeOH/H_2O (0.1% HCO_2H), 3 min, 1.00 mL/min, rt = 1.79 min, *m/z* = 353 $[M-H]^-$. LC-MS (ESI) 50 - 95% MeOH/H_2O (0.1% HCO_2H), 3 min, 1.00 mL/min, rt = 1.35 min, *m/z* = 353 $[M-H]^-$.

Human Thymidylate Synthase Inhibition Assay

Recombinant human thymidylate synthase (hTS) was purchased from Abcam (ab95378). The protein was His-tagged (N-terminal) and >95% pure by SDS-PAGE according to the vendor. dUMP (disodium salt) was purchase from Sigma Aldrich (D3876), FdUMP (disodium salt) was purchased from Sigma Aldrich (F3503), 5,10-methylenetetrahydrofolate (mTHF, calcium salt, folitixorin calcium) was purchased from Boc Sciences (B0084-007873), and all were used without further purification. The cell-free functional hTS assay protocol was modified from literature procedures:

- Lovelace *et al*., Cooperative Inhibition of Human TS by Mixtures of Active Site Binding and Allosteric Inhibitors, *Biochemistry* **2007**, *46*, 2823-2830.
- Cardinale *et al*., Protein-Protein Interface-Binding Peptides Inhibit the Cancer Therapy Target Human TS, *Proc. Nat. Acad. Sci.* **2011**, *108(34)*, e542-e549.
- Islam *et al*., Concerted versus Stepwise Mechanism in TS, *J. Am. Chem. Soc.* **2014**, *136*, 9850-9853.
- Salo-Ahen *et al*., Hotspots in an Obligate Homodimeric Anticancer Target, Structural and Functional Effects of Interfacial Mutations in Human TS, *J. Med. Chem.* **2015**, *58*, 3572-3581.

Inhibition assays were performed using a BioTek Synergy Neo2 plate reader equipped with a Xenon flash lamp and a photodiode array detector. The hTS reaction was conducted at room temperature in a buffer solution (pH 7.5) containing 50 mM Tris-HCl, 1 mM EDTA, 25 mM $MgCl_2$, and 5 mM HCHO. The reaction system (175 μL total volume, 1 cm optical path length) contained 50 μM dUMP (primary substrate), 250 μM mTHF (co-substrate and enzyme cofactor), 0.84 μM hTS (enzyme), and decreasing concentrations of inhibitor (FdUMP or a 5′-substitued analog thereof). mTHF was added last to initiate the reaction. Formation of the co-product, dihydrofolate, was monitored at 340 nm. Reaction rates were calculated by plotting the absorbance at 340 nm against the reaction time and performing linear regression analysis to determine the slope. The reaction rates at different inhibitor concentrations were normalized to the reaction rate determined in the absence of inhibitor (100% enzymatic activity). Concentration-response curves were constructed by plotting the resulting mean % enzymatic activity values (from at least three independent experiments) against log[inhibitor]. IC_{50} values were then calculated using non-linear regression (four-parameter logistic equation, *see* Equation 1 below) in the GraphPad Prism v.9 software.

Equation 1: $$Y = Y_{min} + (Y_{max} - Y_{min}) / [1 + 10^{((\log IC50 - X) \times \text{Hill Slope})}]$$

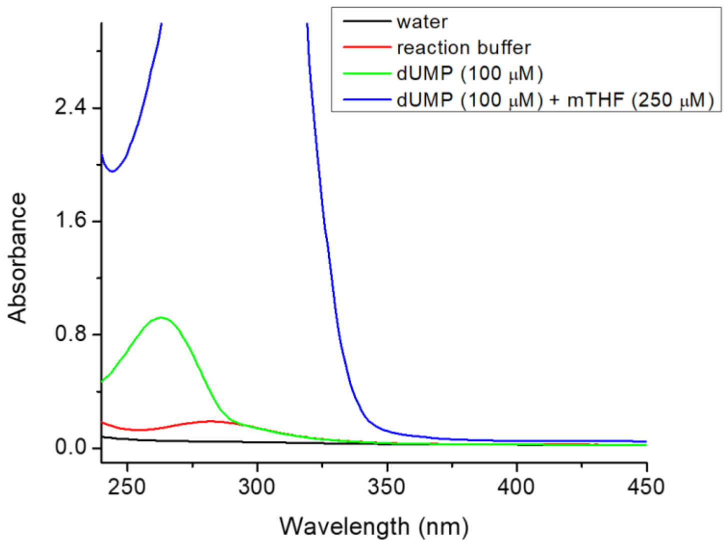

Figure S1: Overlaid uv absorption specta for water, reaction buffer, dUMP, and dUMP + mTHF. Although mTHF is highly uv active, the absorbance at 340 nm is relatively low. In contrast, absorbance of the byproduct DHF at 340 nm is quite intense, which allows selective monitoring of product formation at this wavelength.

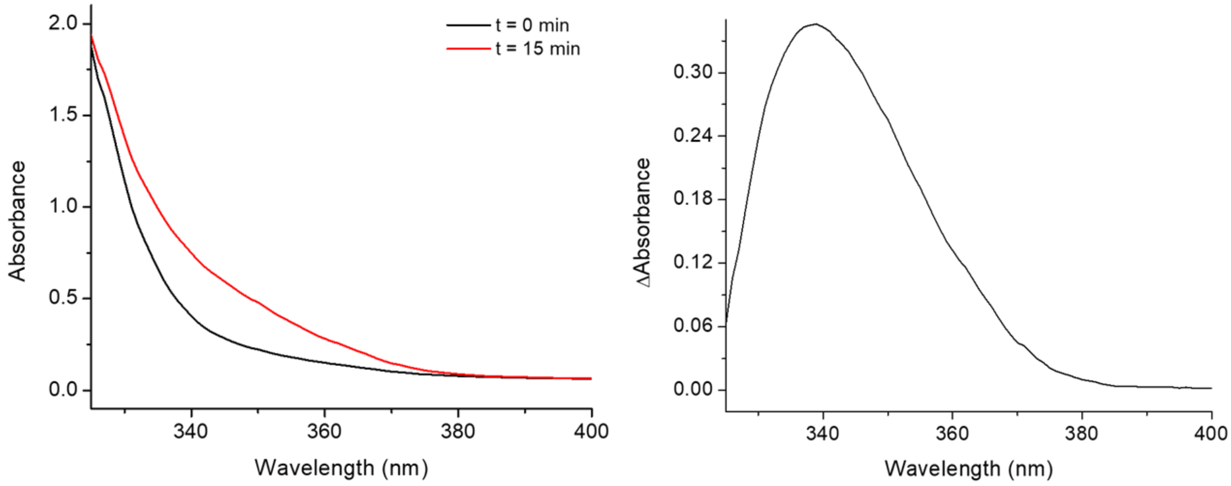

Figure S2: After reaction initiation, an increase in absorbance at 340 nm is measured and indicative of TS-catalyzed conversion of mTHF and dUMP to DHF and dTMP. Subtracting the uv absorbance spectrum measured prior to reaction initiation from the spectrum measured after 15 min creates a Δabsorbance plot that indicates maximum Δabsorbance at 340 nm.

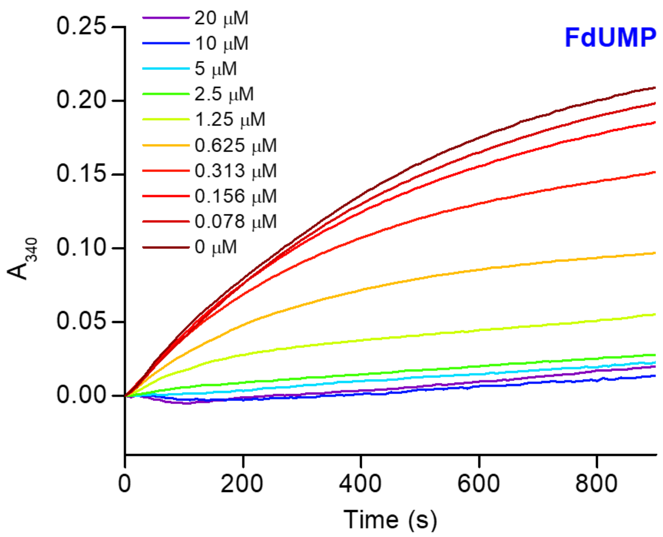

Figure S3: Representative kinetic plot demonstrating concentration-dependent inhibition of TS by FdUMP, as measured by absorbance at 340 nm after reaction initiation.

Compound	hTS Region	FEP ΔΔG (kcal/mol)	hTS IC_{50} (μM)
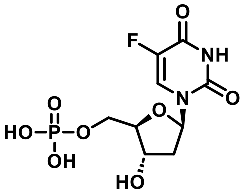 FdUMP	n/a	Reference (0.00)	1.13
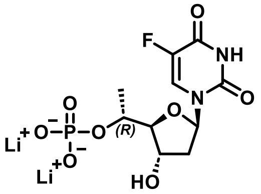 5'-(*R*)-CH_3 FdUMP (**1**)	Green	-0.62	1.21
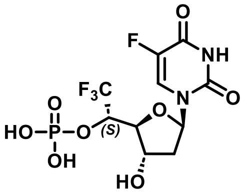 5'-(*S*)-CF_3 FdUMP (**4**)	Green	-5.24	1.24
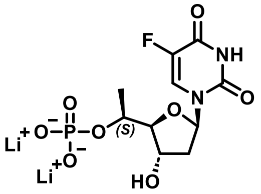 5'-(*S*)-CH_3 FdUMP (**2**)	Red	+4.46	>20
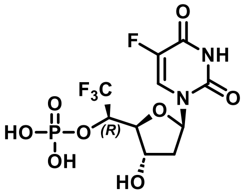 5'-(*R*)-CF_3-FdUMP	Red	not determined	>20
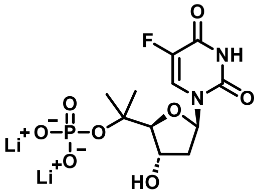 5'-*gem*-$(CH_3)_2$-FdUMP (**3**)	Green and Red	+3.60	>20

Table S6: Summary of FEP relative binding energy predictions and biochemical assay potencies.

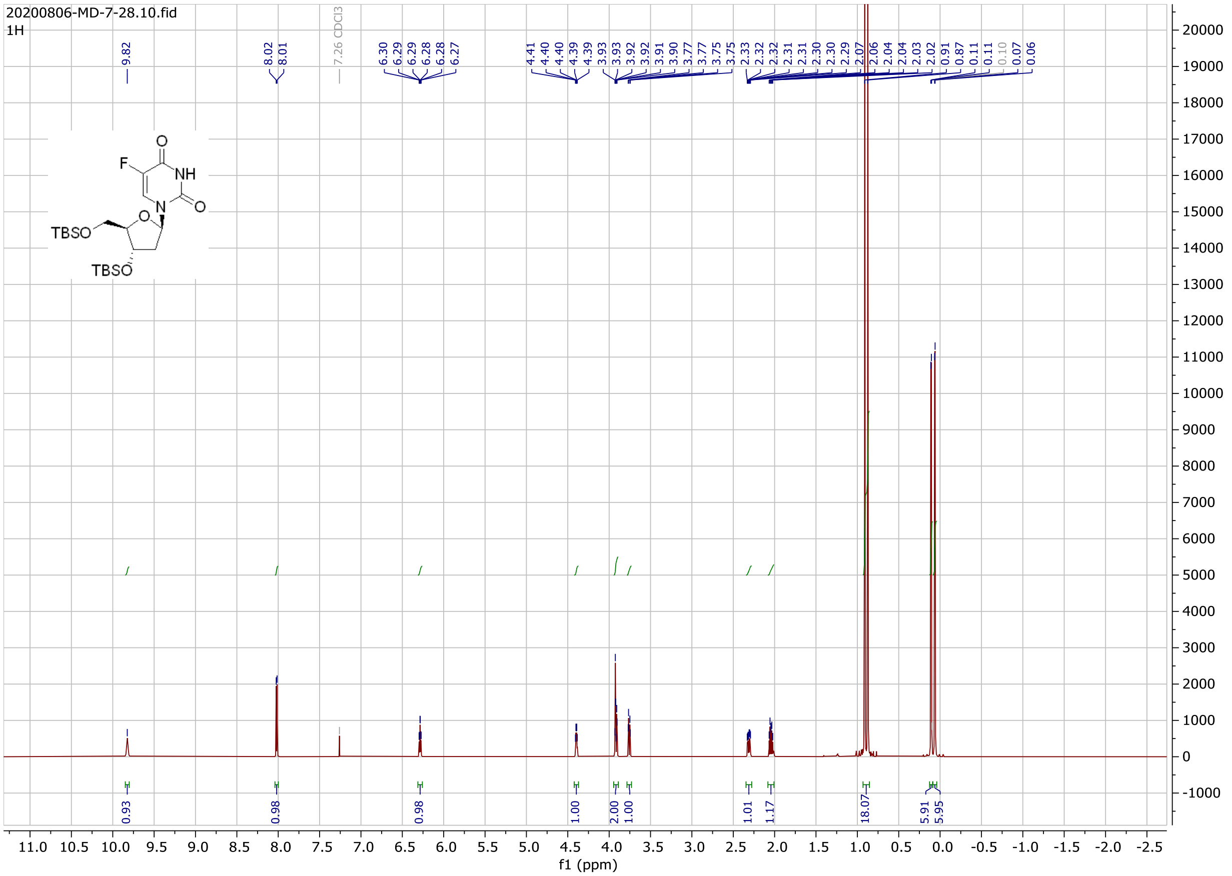

20200806-MD-7-28.10.fid
1H
f1 (ppm)

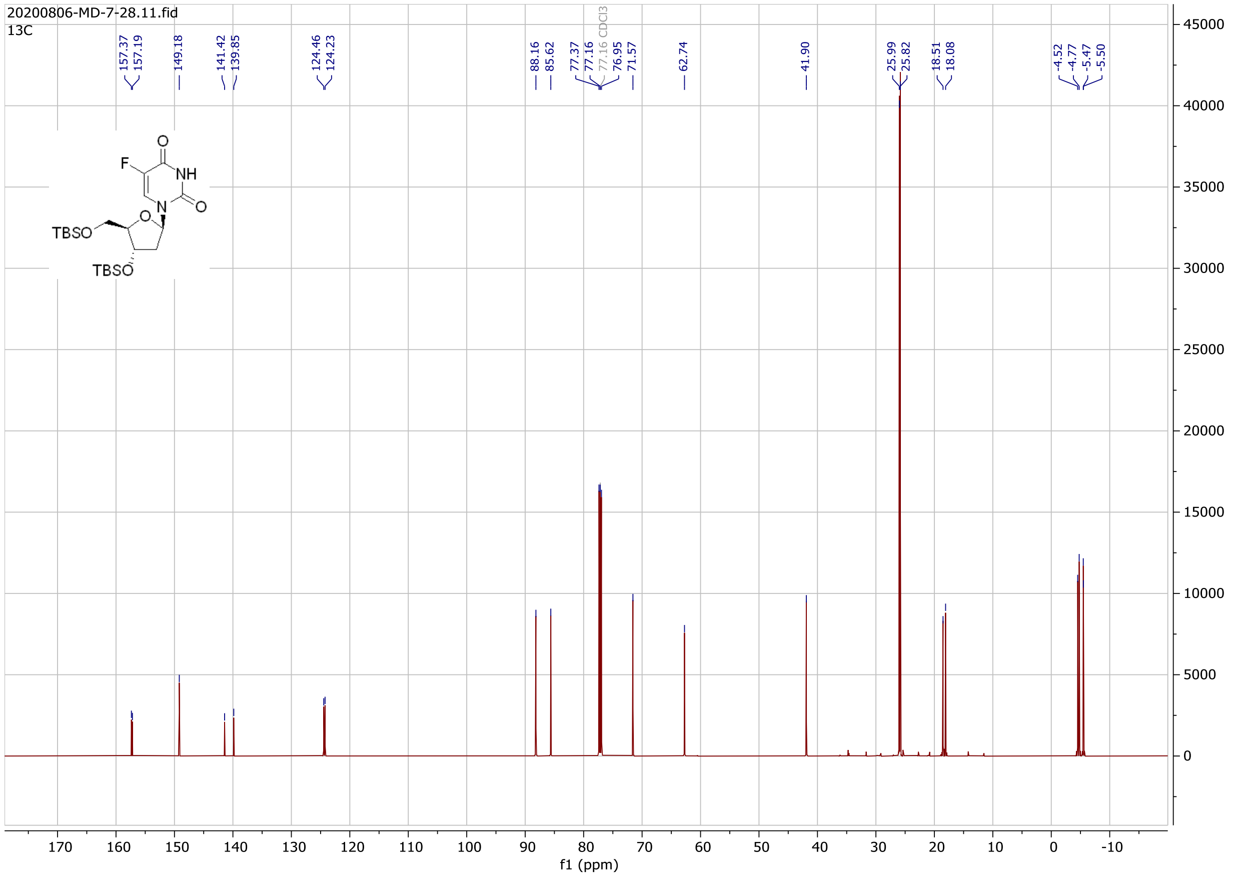
20200806-MD-7-28.11.fid
13C
157.37
157.19
149.18
141.42
139.85
124.46
124.23
88.16
85.62
77.37
77.16
77.16 CDCl3
76.95
71.57
62.74
41.90
25.99
25.82
18.51
18.08
-4.52
-4.77
-5.47
-5.50
F
O
NH
N
O
O
TBSO
TBSO
f1 (ppm)

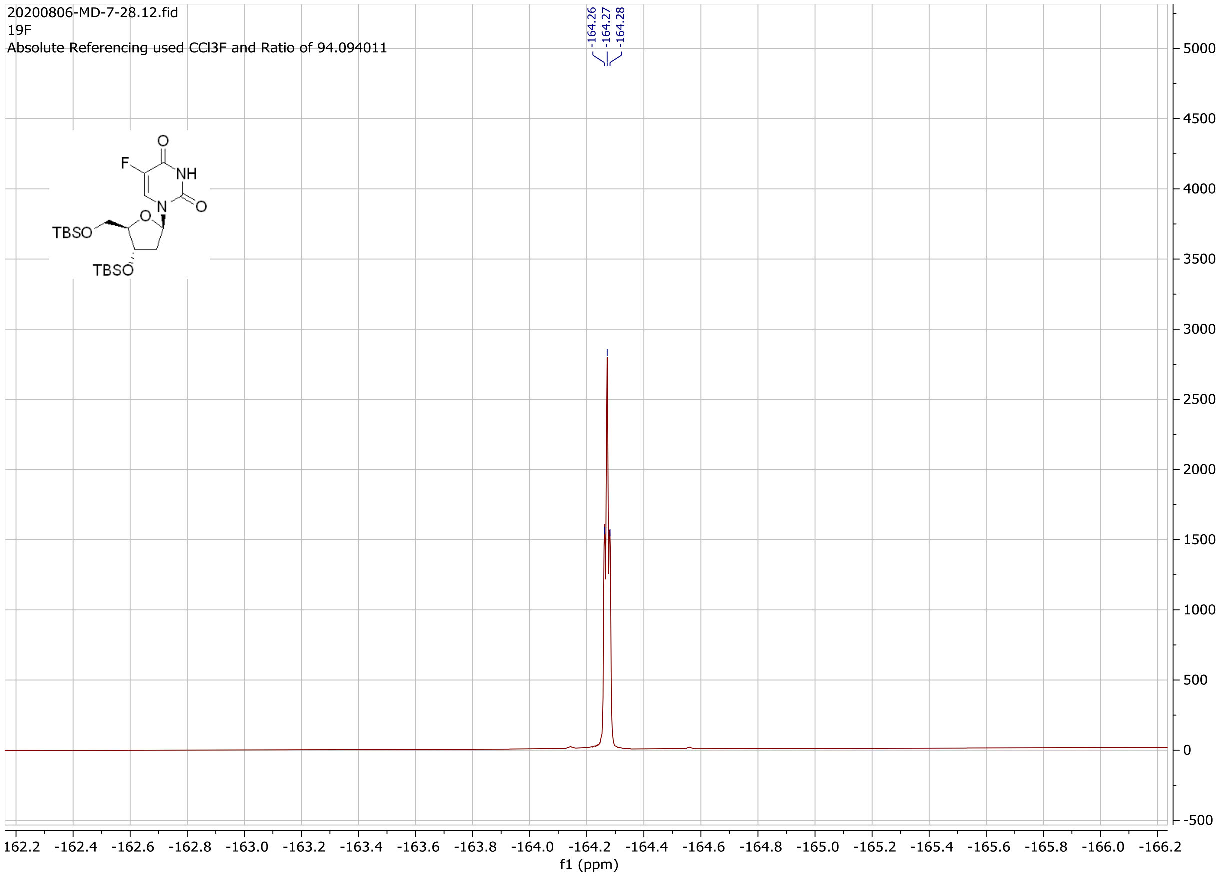
20200806-MD-7-28.12.fid
19F
Absolute Referencing used CCl3F and Ratio of 94.094011
-164.26
-164.27
-164.28
F
O
NH
N
O
O
TBSO
TBSO
f1 (ppm)

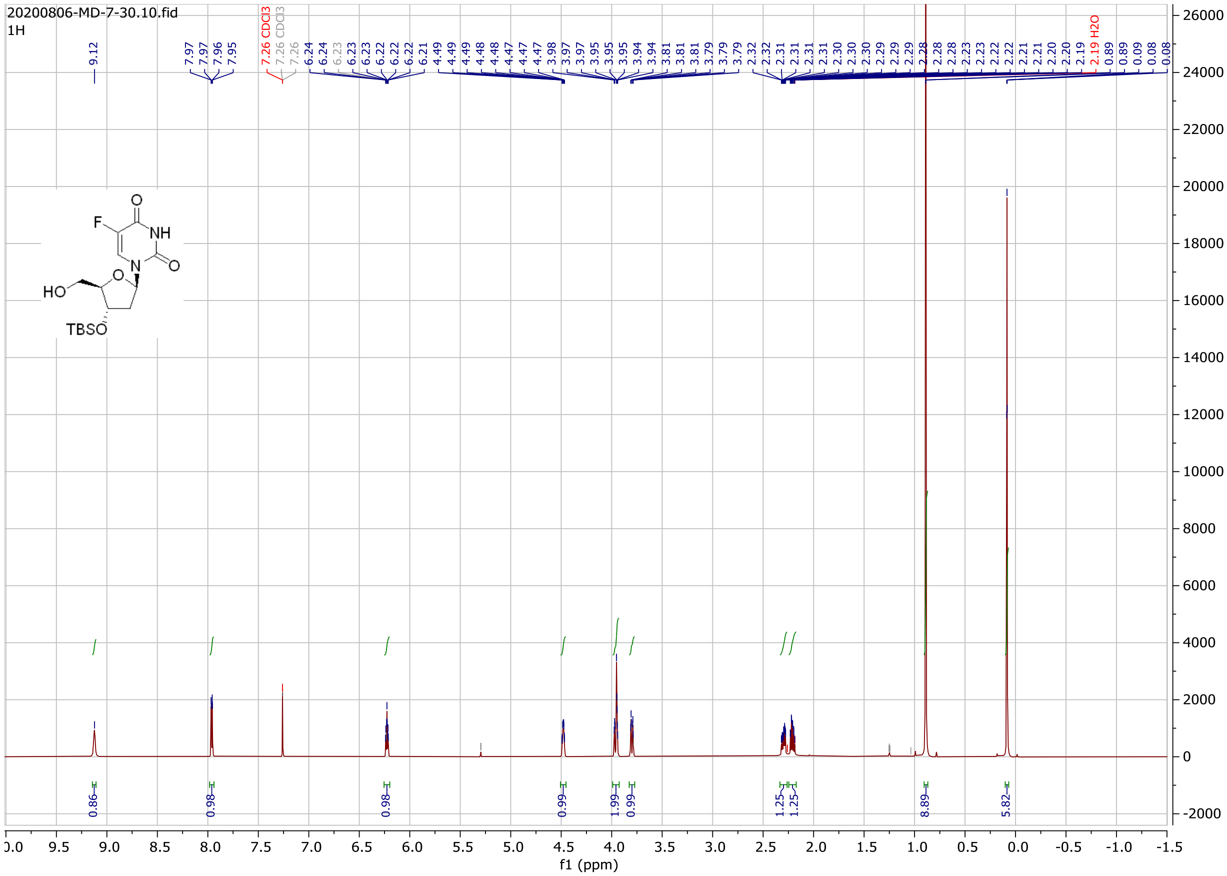
20200806-MD-7-30.10.fid
1H
f1 (ppm)
HO
TBSO
F
NH
N
O

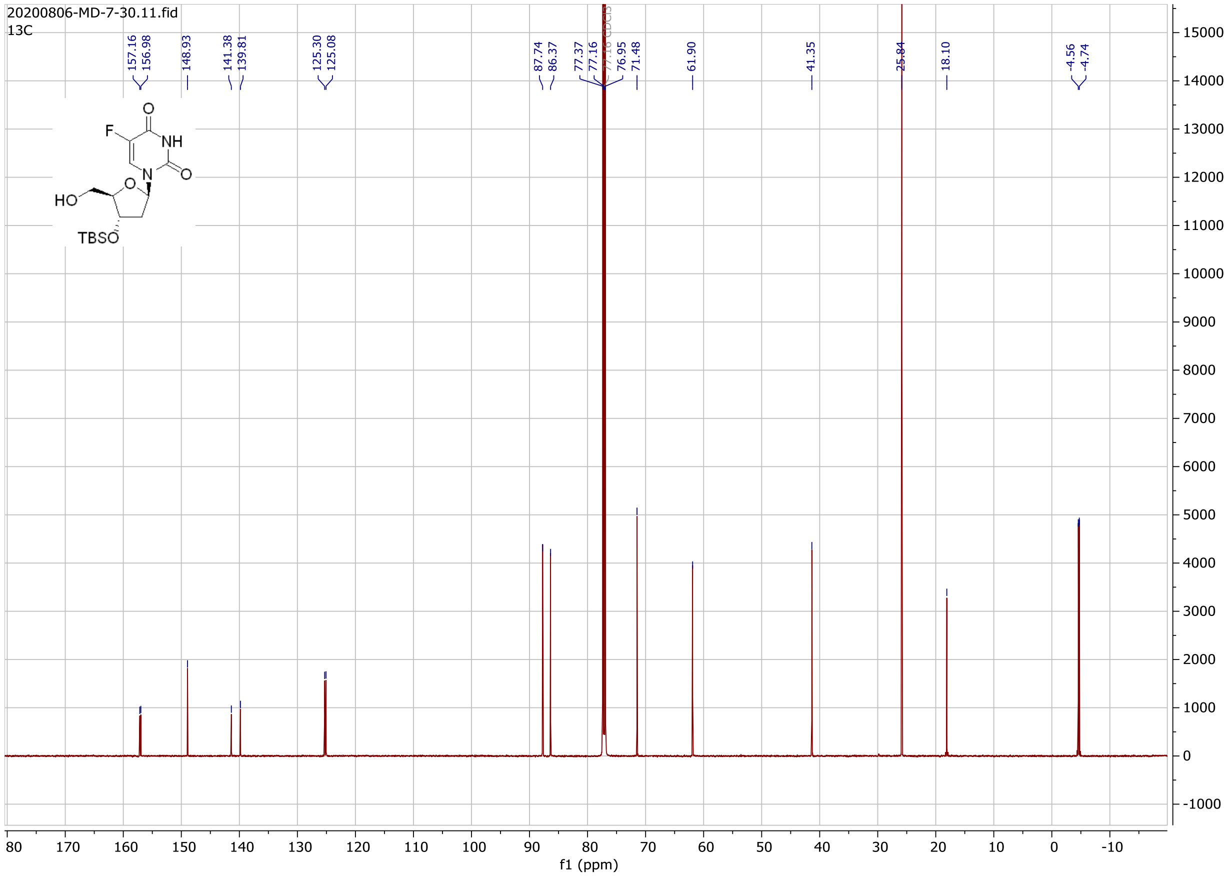
20200806-MD-7-30.11.fid
13C
157.16
156.98
148.93
141.38
139.81
125.30
125.08
87.74
86.37
77.37
77.16
76.95
71.48
61.90
41.35
25.84
18.10
-4.56
-4.74
F
O
NH
N
O
HO
TBSO
f1 (ppm)

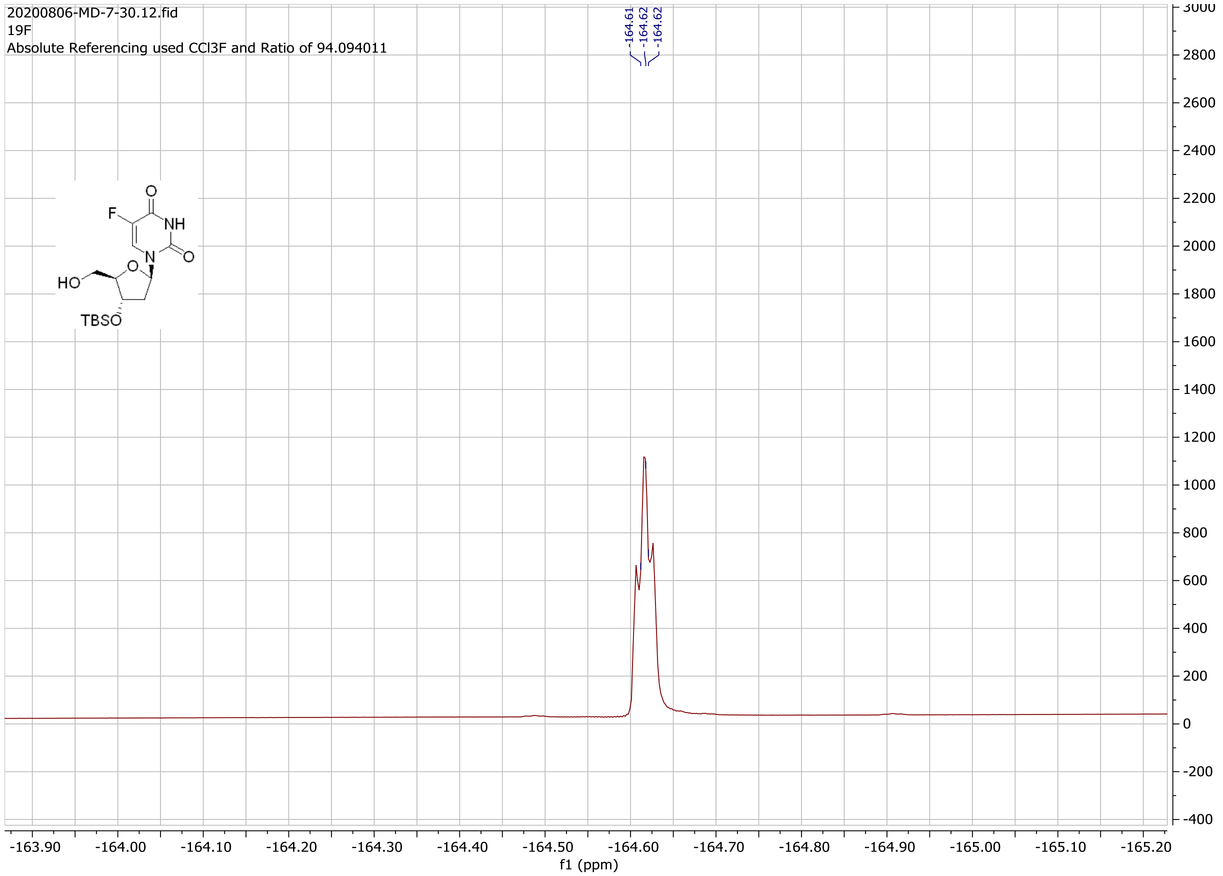
20200806-MD-7-30.12.fid
19F
Absolute Referencing used CCl3F and Ratio of 94.094011
-164.61
-164.62
-164.62
F
O
NH
N
O
HO
O
TBSO
f1 (ppm)

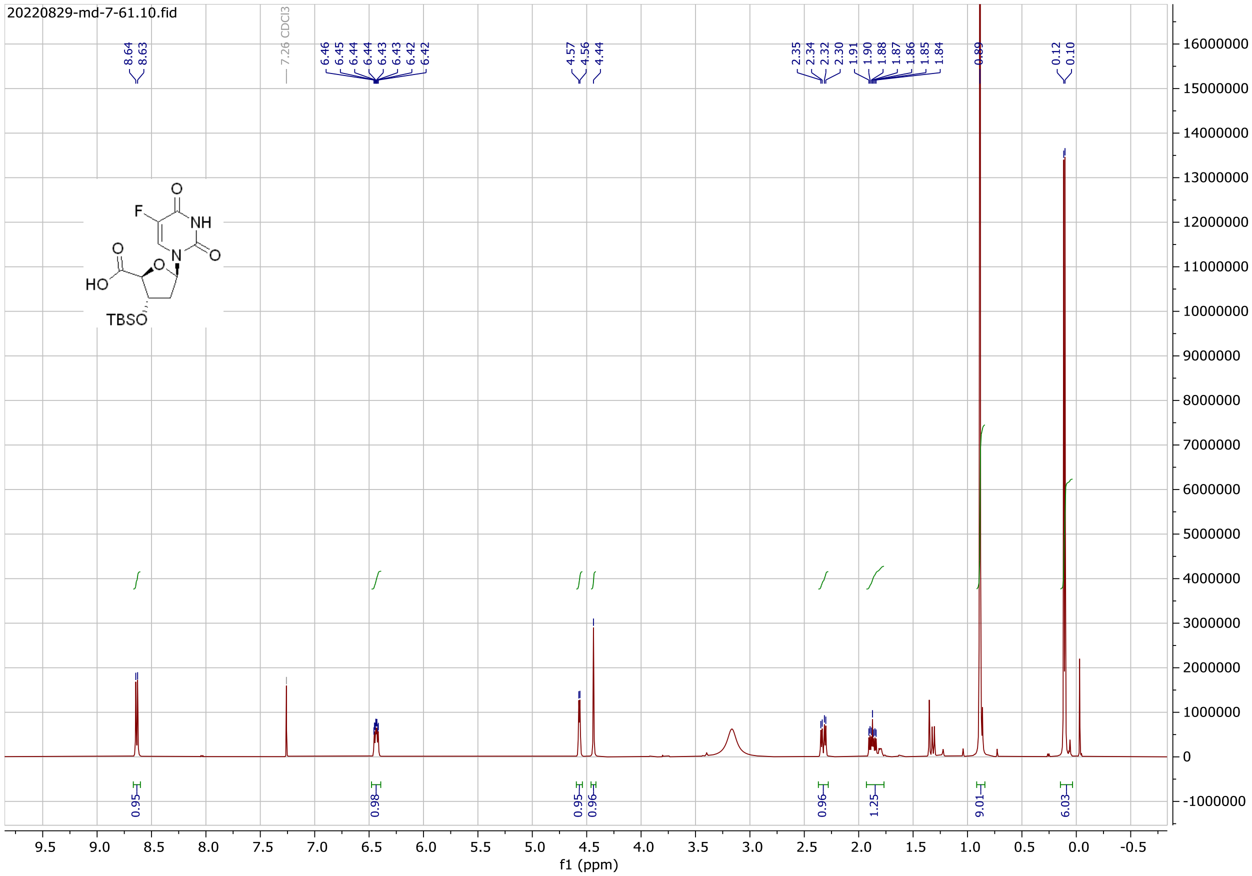
20220829-md-7-61.10.fid
f1 (ppm)

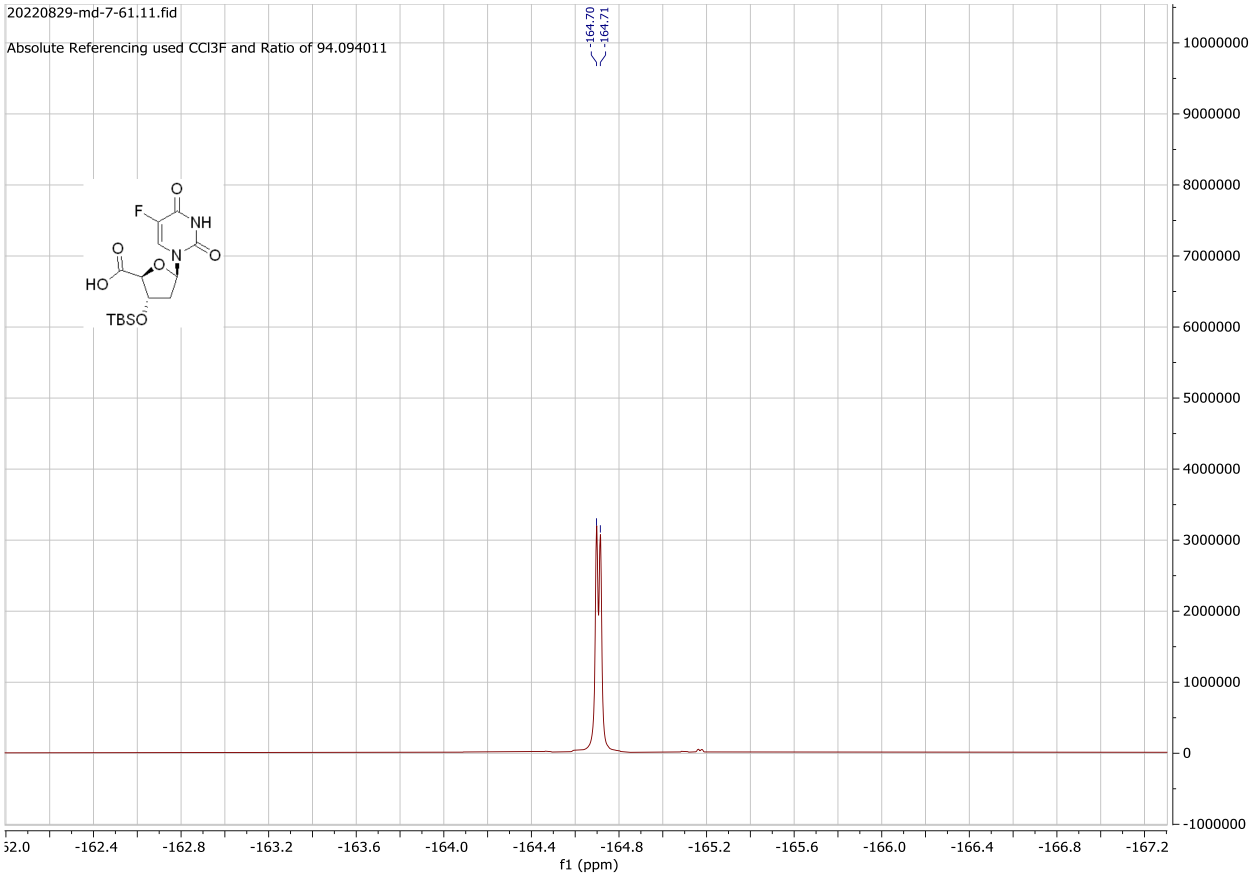

20220829-md-7-61.11.fid
Absolute Referencing used CCl3F and Ratio of 94.094011
-164.70
-164.71
F
O
NH
O
N
O
O
HO
TBSO
10000000
9000000
8000000
7000000
6000000
5000000
4000000
3000000
2000000
1000000
0
-1000000
-162.4
-162.8
-163.2
-163.6
-164.0
-164.4
-164.8
-165.2
-165.6
-166.0
-166.4
-166.8
-167.2
f1 (ppm)

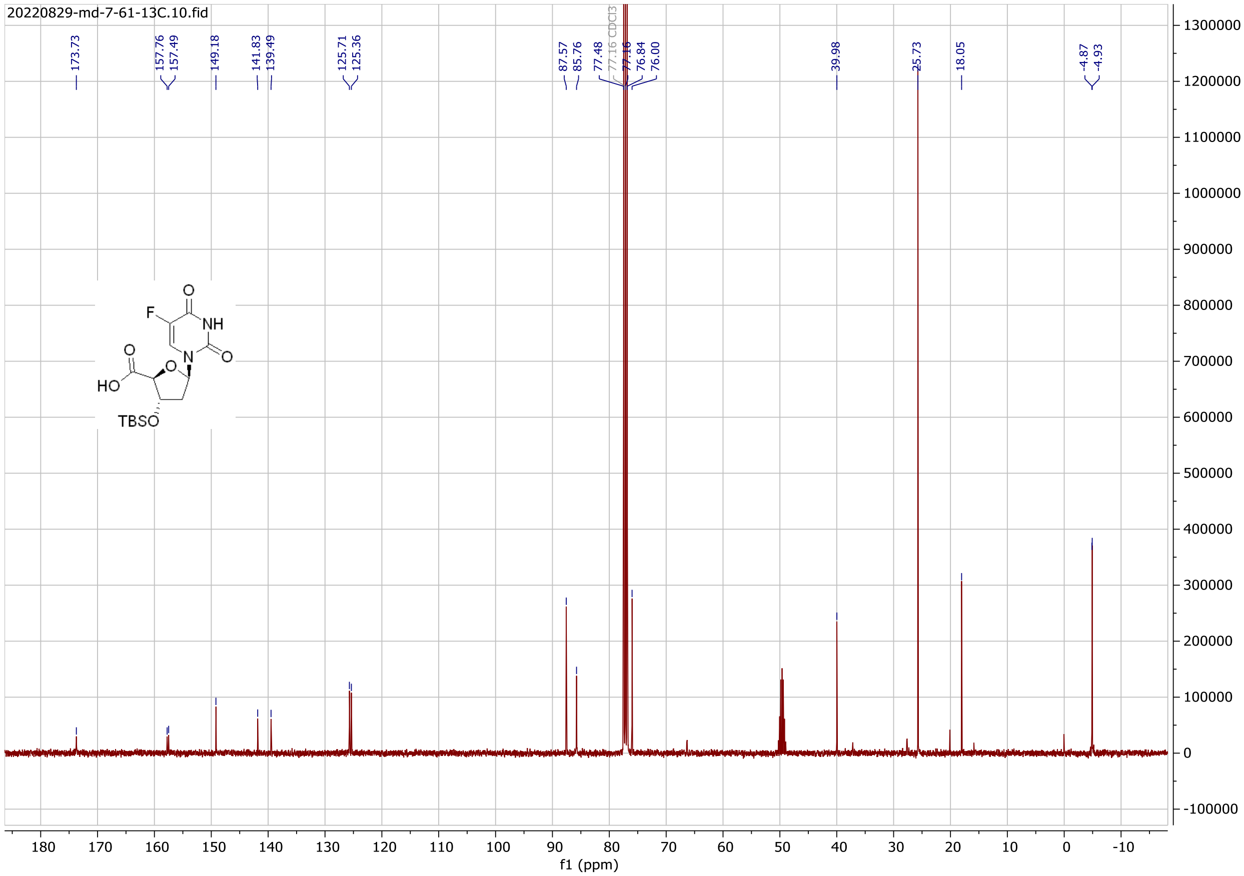
20220829-md-7-61-13C.10.fid
173.73
157.76
157.49
149.18
141.83
139.49
125.71
125.36
87.57
85.76
77.48
77.16 CDCl3
77.16
76.84
76.00
39.98
25.73
18.05
-4.87
-4.93
F
O
NH
O
N
O
O
HO
TBSO
f1 (ppm)

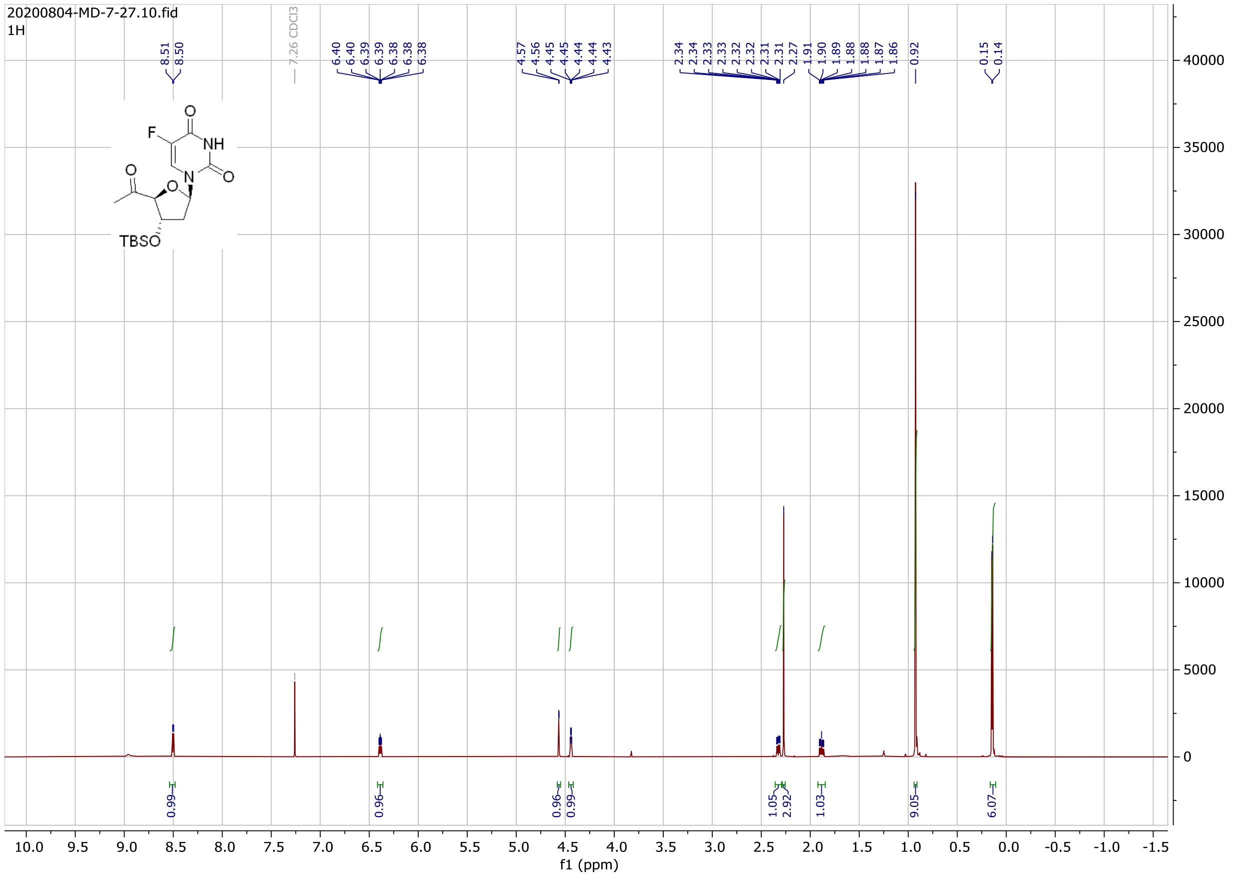
20200804-MD-7-27.10.fid
1H
8.51
8.50
7.26 CDCl3
6.40
6.40
6.39
6.39
6.38
6.38
6.38
4.57
4.56
4.45
4.45
4.44
4.44
4.43
2.34
2.34
2.33
2.33
2.32
2.32
2.31
2.31
2.27
1.91
1.90
1.89
1.88
1.88
1.87
1.86
0.92
0.15
0.14
F
O
NH
N
O
O
O
TBSO
0.99
0.96
0.96
0.99
1.05
2.92
1.03
9.05
6.07
f1 (ppm)

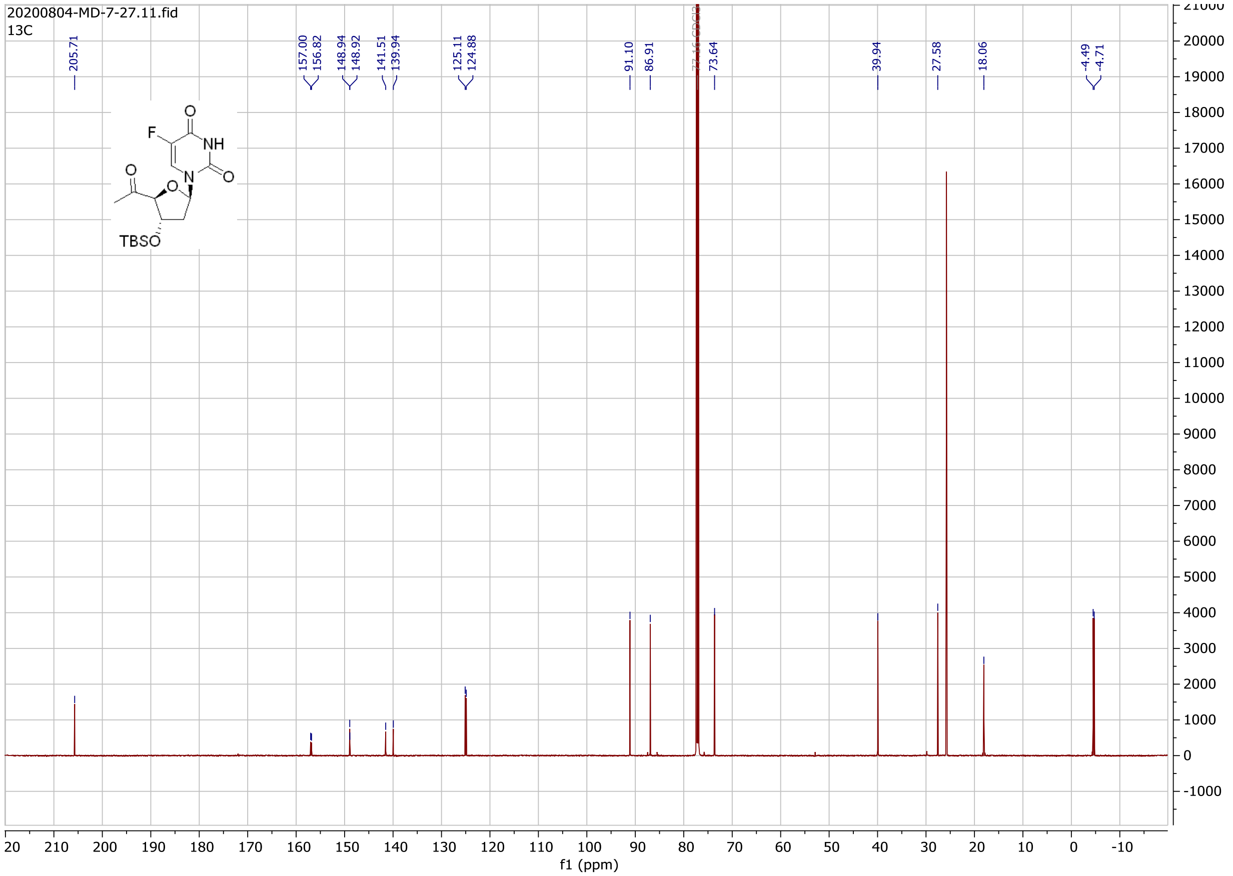
20200804-MD-7-27.11.fid
13C
205.71
157.00
156.82
148.94
148.92
141.51
139.94
125.11
124.88
91.10
86.91
73.64
39.94
27.58
18.06
-4.49
-4.71
F
O
NH
N
O
O
O
TBSO
f1 (ppm)

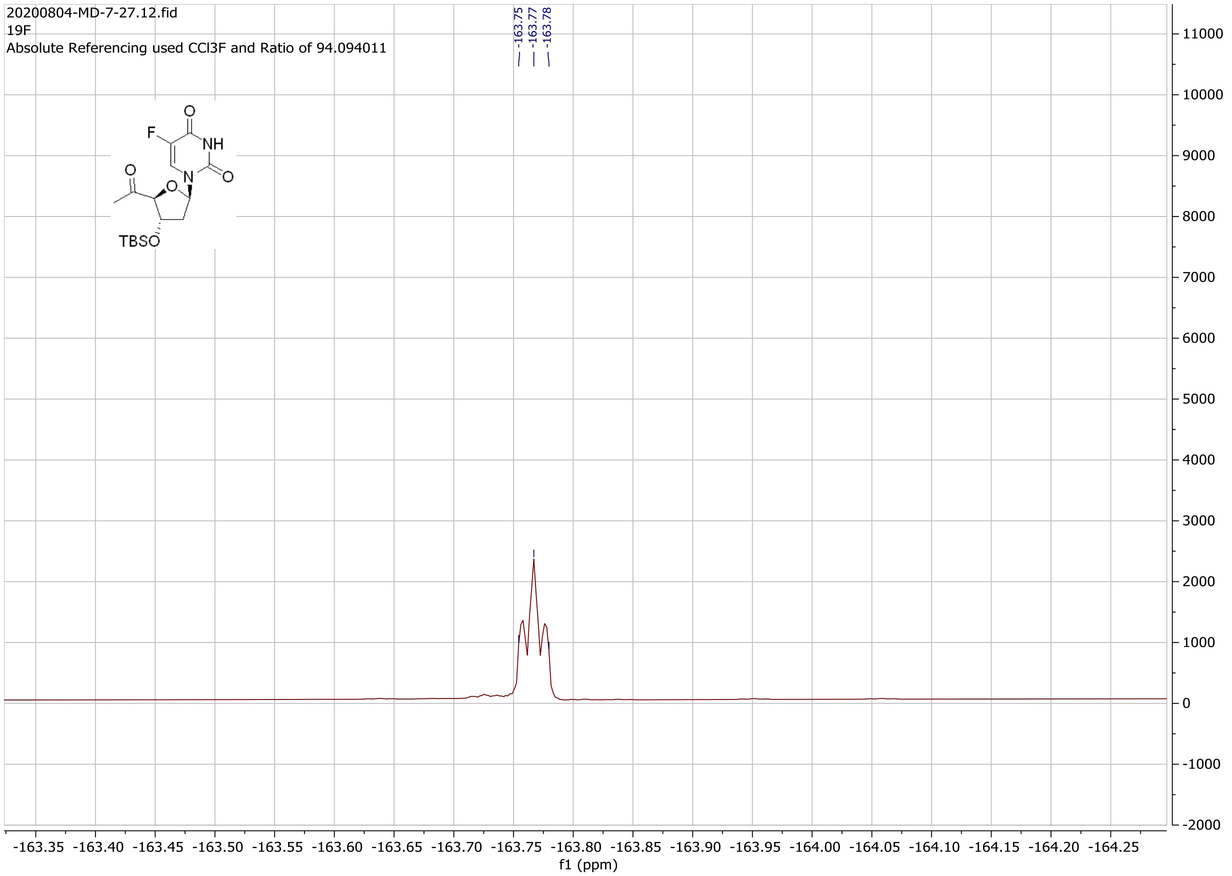
20200804-MD-7-27.12.fid
19F
Absolute Referencing used CCl3F and Ratio of 94.094011
-163.75
-163.77
-163.78
F
O
NH
N
O
O
O
TBSO
f1 (ppm)

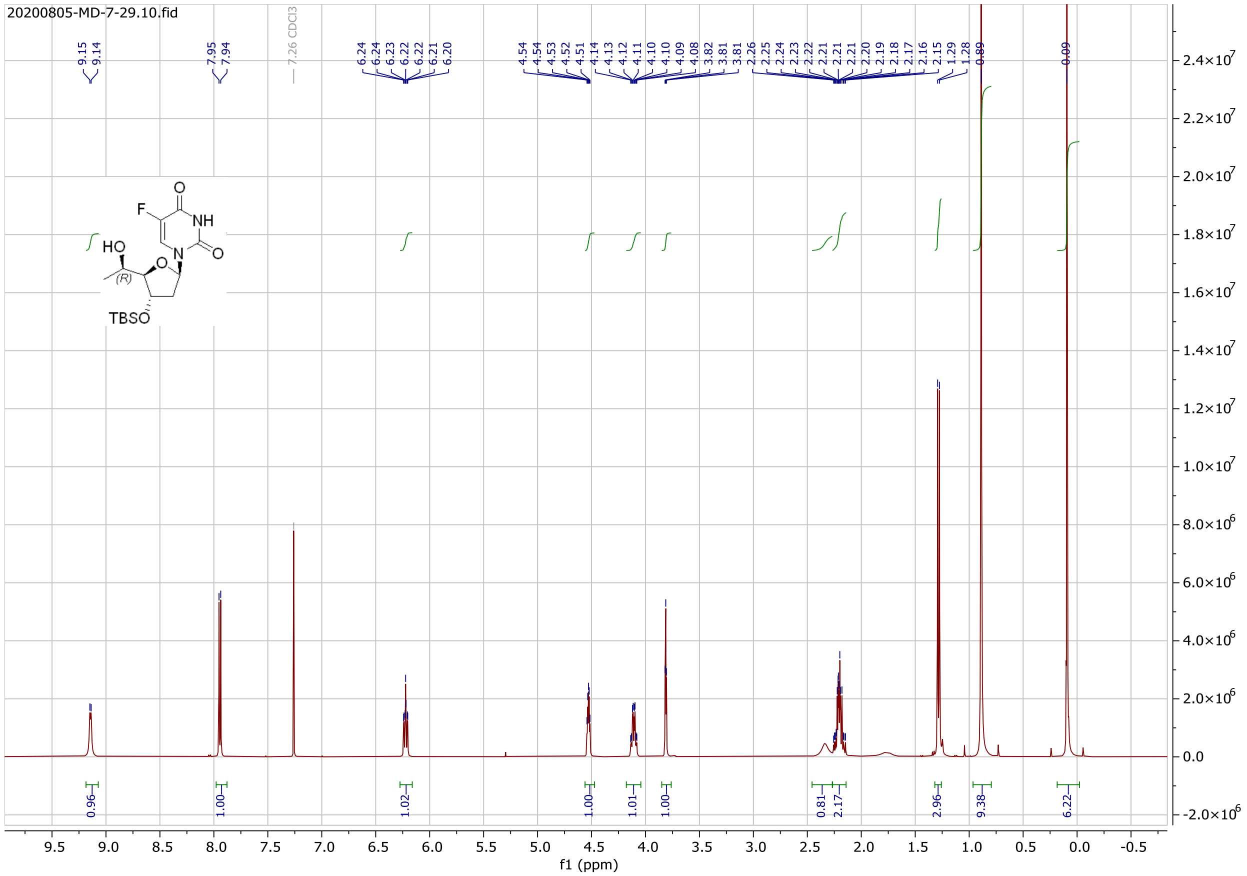

20200805-MD-7-29.10.fid
7.26 CDCl3
f1 (ppm)

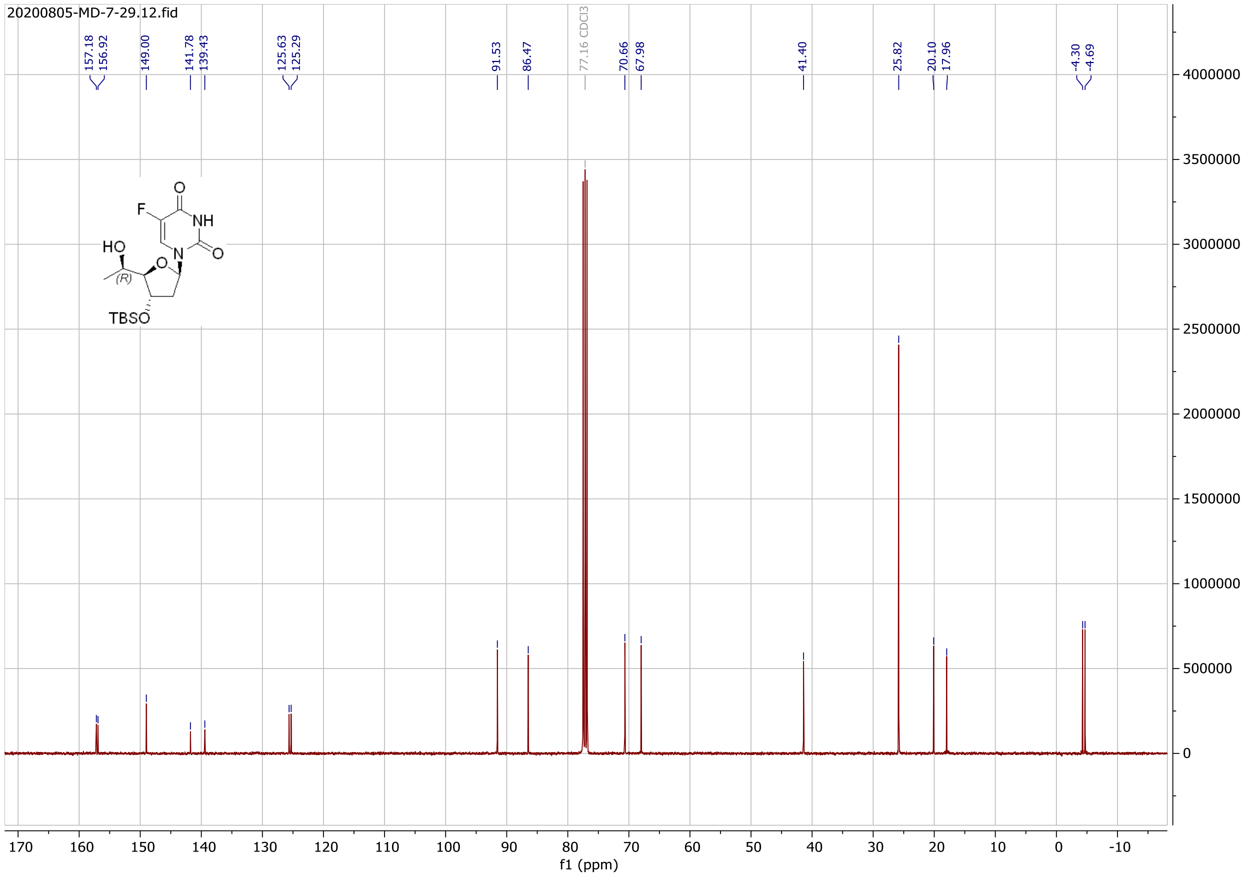

20200805-MD-7-29.12.fid
157.18
156.92
149.00
141.78
139.43
125.63
125.29
91.53
86.47
77.16 CDCl3
70.66
67.98
41.40
25.82
20.10
17.96
-4.30
-4.69
F
O
NH
N
O
HO
O
(R)
TBSO
f1 (ppm)

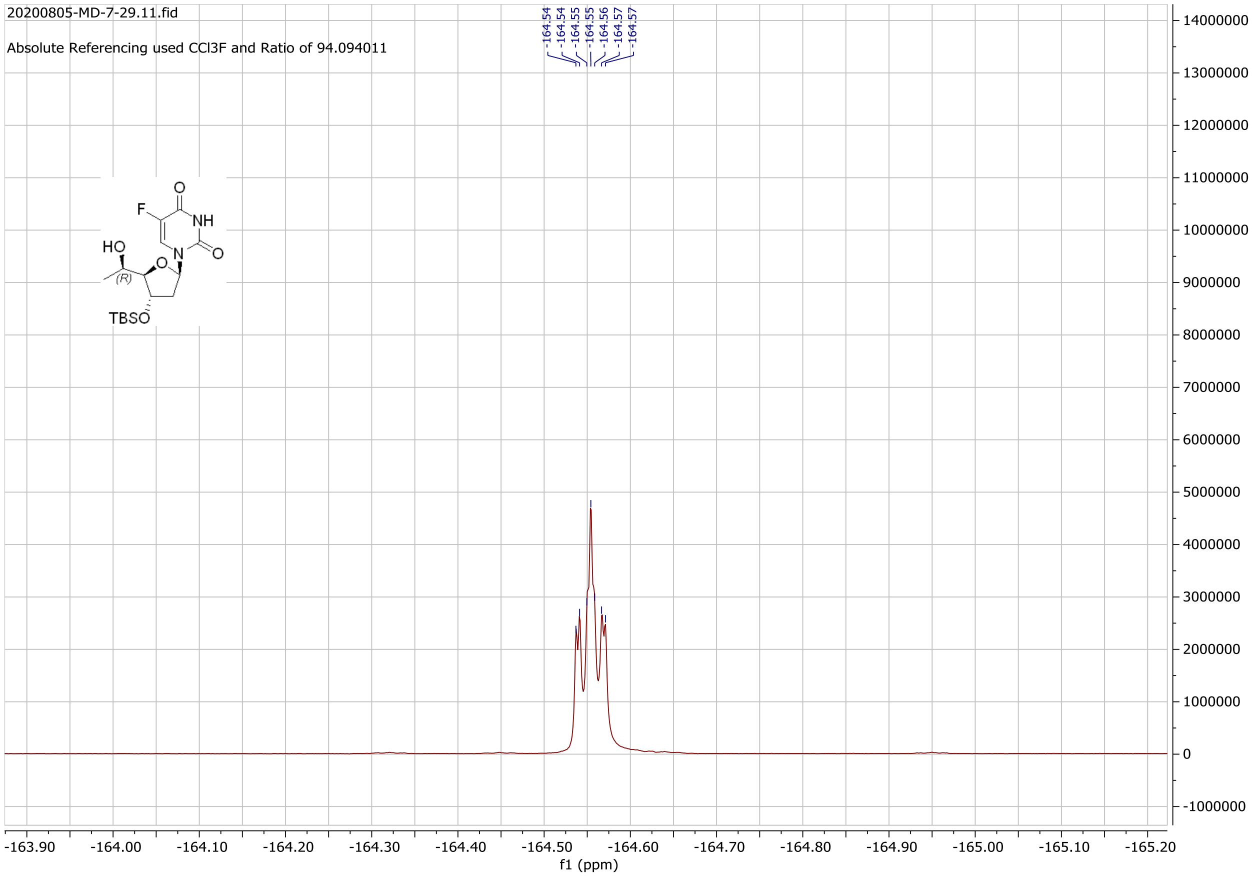
20200805-MD-7-29.11.fid
Absolute Referencing used CCl3F and Ratio of 94.094011
-164.54
-164.54
-164.55
-164.55
-164.56
-164.57
-164.57
f1 (ppm)

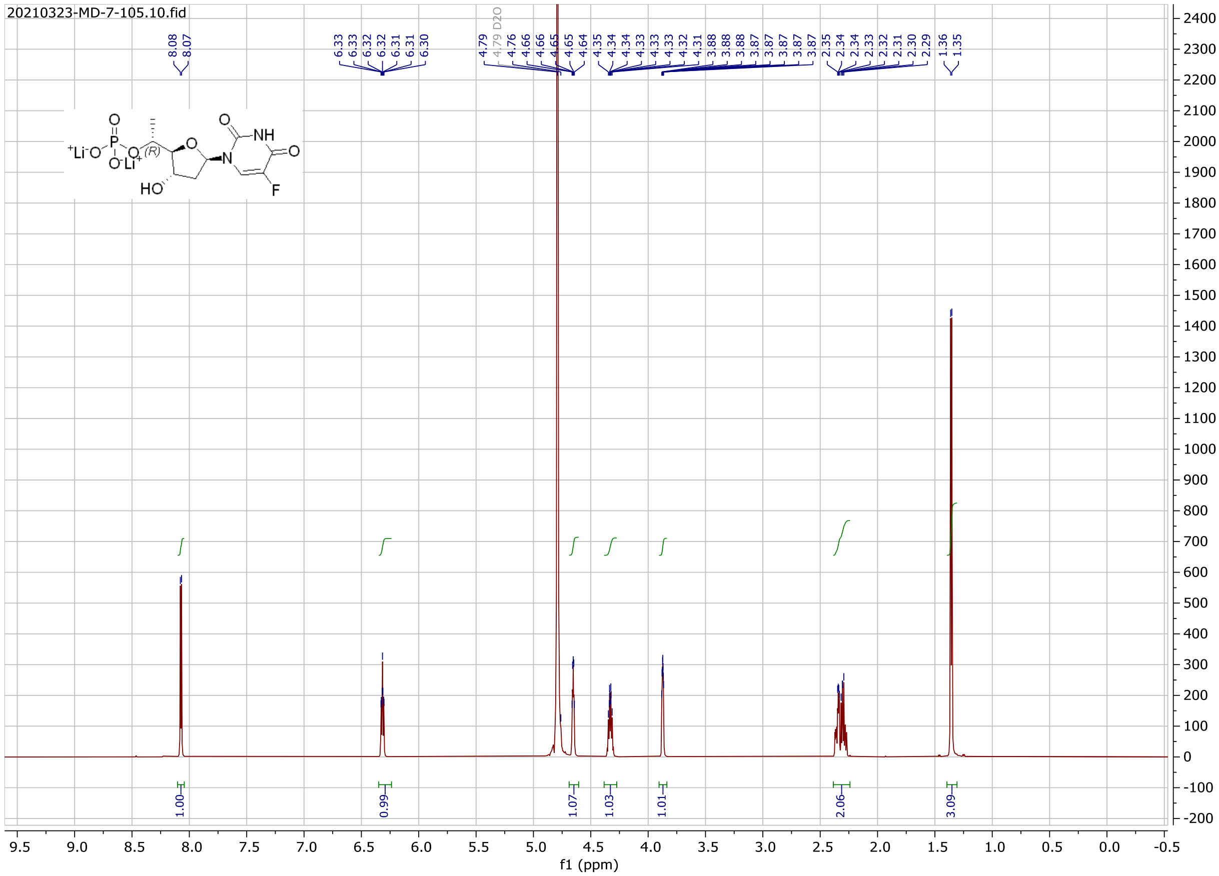
20210323-MD-7-105.10.fid
8.08
8.07
6.33
6.33
6.32
6.32
6.31
6.31
6.30
4.79
4.79 D2O
4.76
4.66
4.66
4.65
4.65
4.64
4.35
4.34
4.34
4.33
4.33
4.33
4.32
4.31
3.88
3.88
3.88
3.87
3.87
3.87
3.87
2.35
2.34
2.34
2.33
2.32
2.31
2.30
2.29
1.36
1.35
1.00
0.99
1.07
1.03
1.01
2.06
3.09
f1 (ppm)

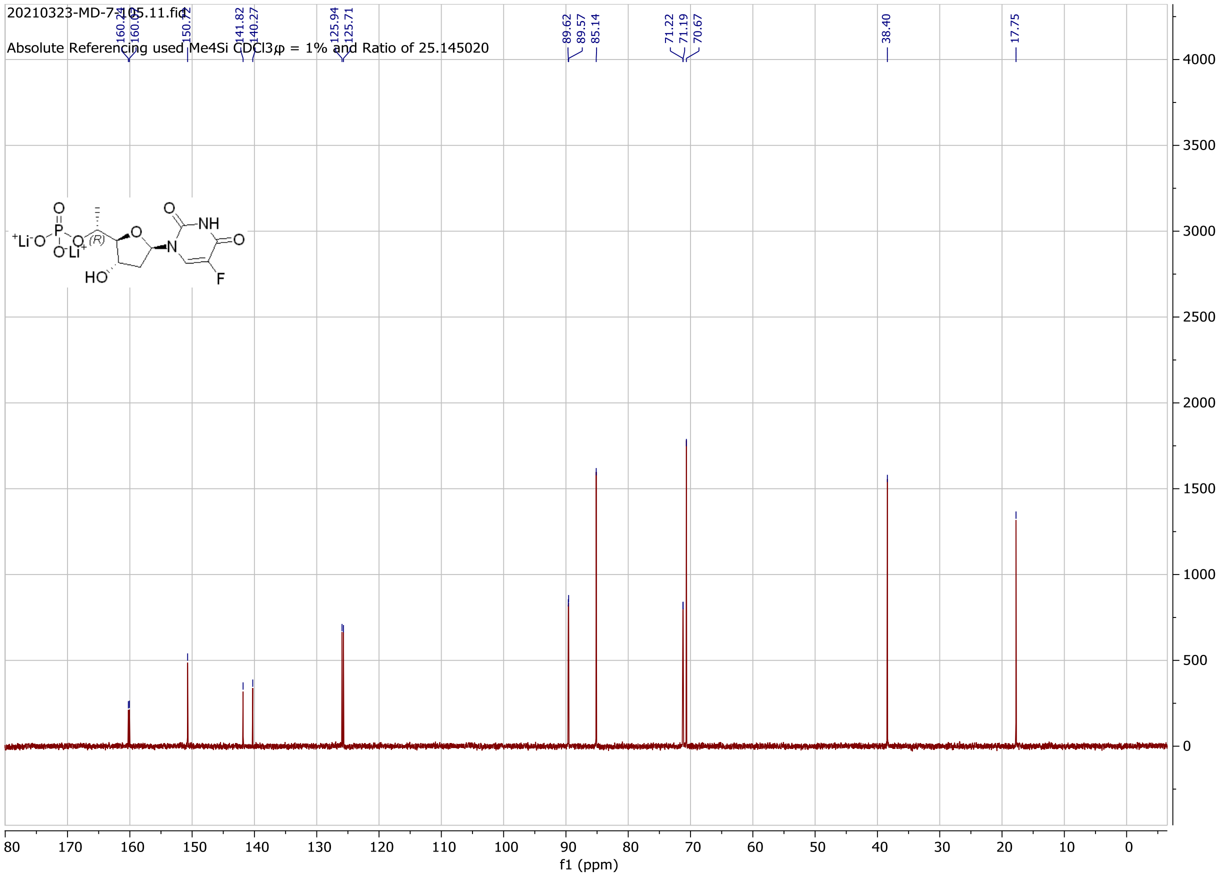
20210323-MD-7-105.11.fid
Absolute Referencing used Me4Si CDCl3,φ = 1% and Ratio of 25.145020
160.24
160.05
150.72
141.82
140.27
125.94
125.71
89.62
89.57
85.14
71.22
71.19
70.67
38.40
17.75
f1 (ppm)

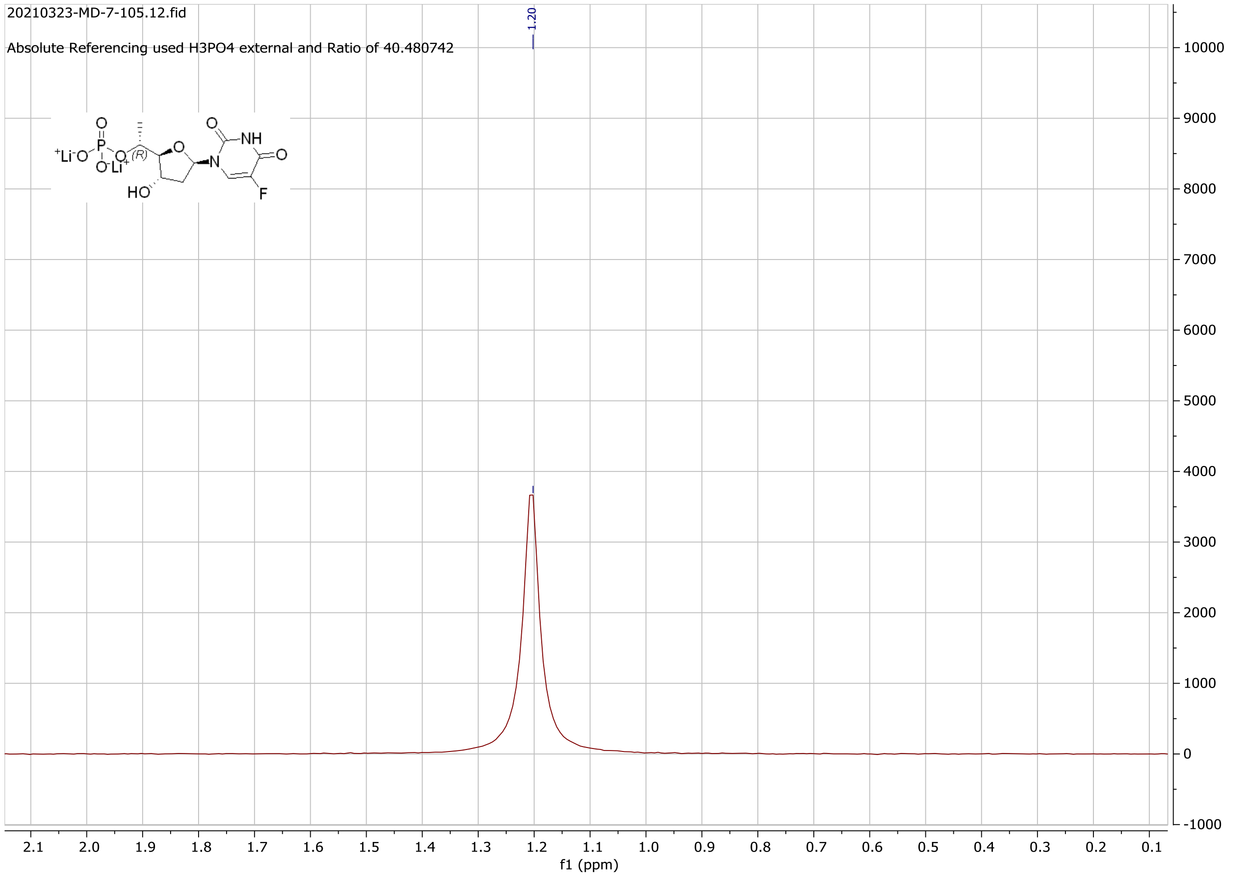
20210323-MD-7-105.12.fid
Absolute Referencing used H3PO4 external and Ratio of 40.480742
1.20
f1 (ppm)

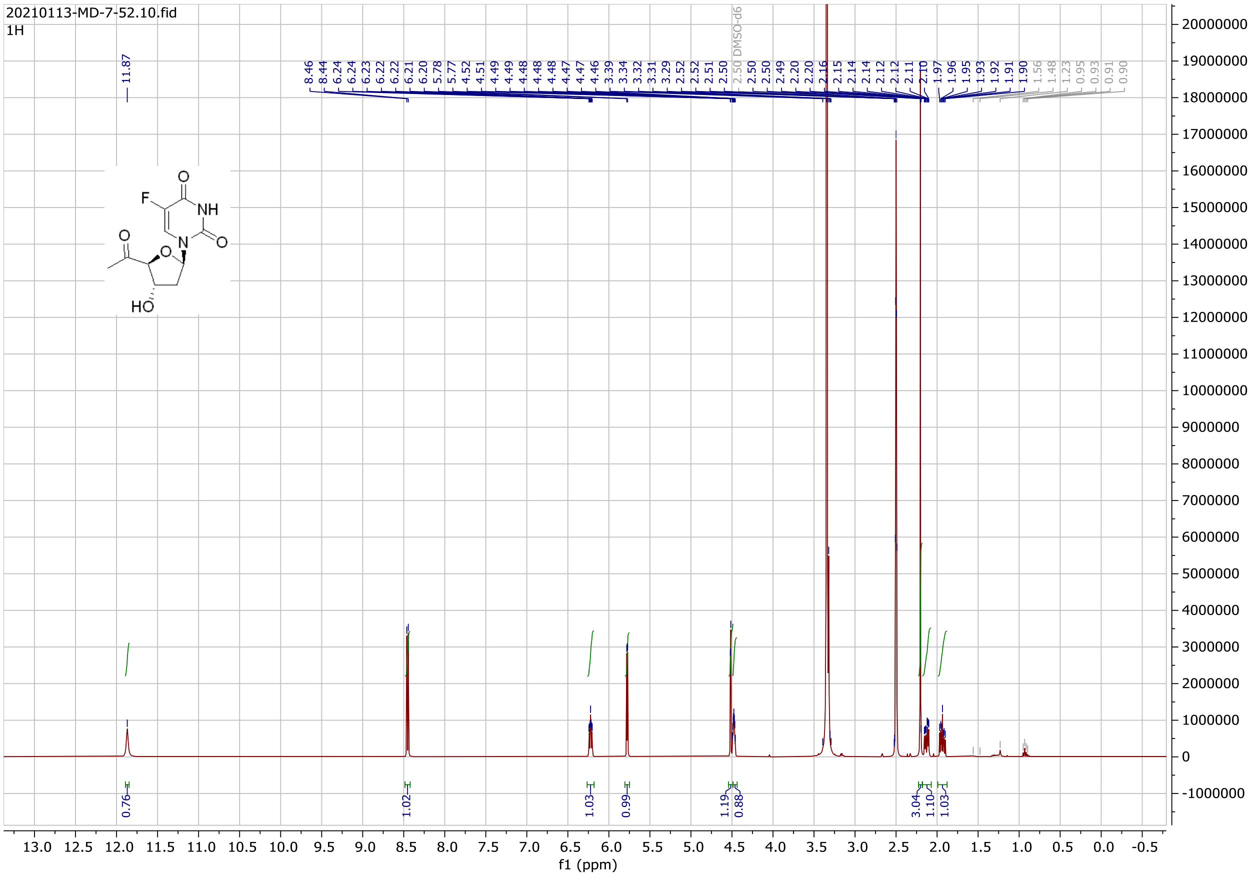
20210113-MD-7-52.10.fid
1H
f1 (ppm)

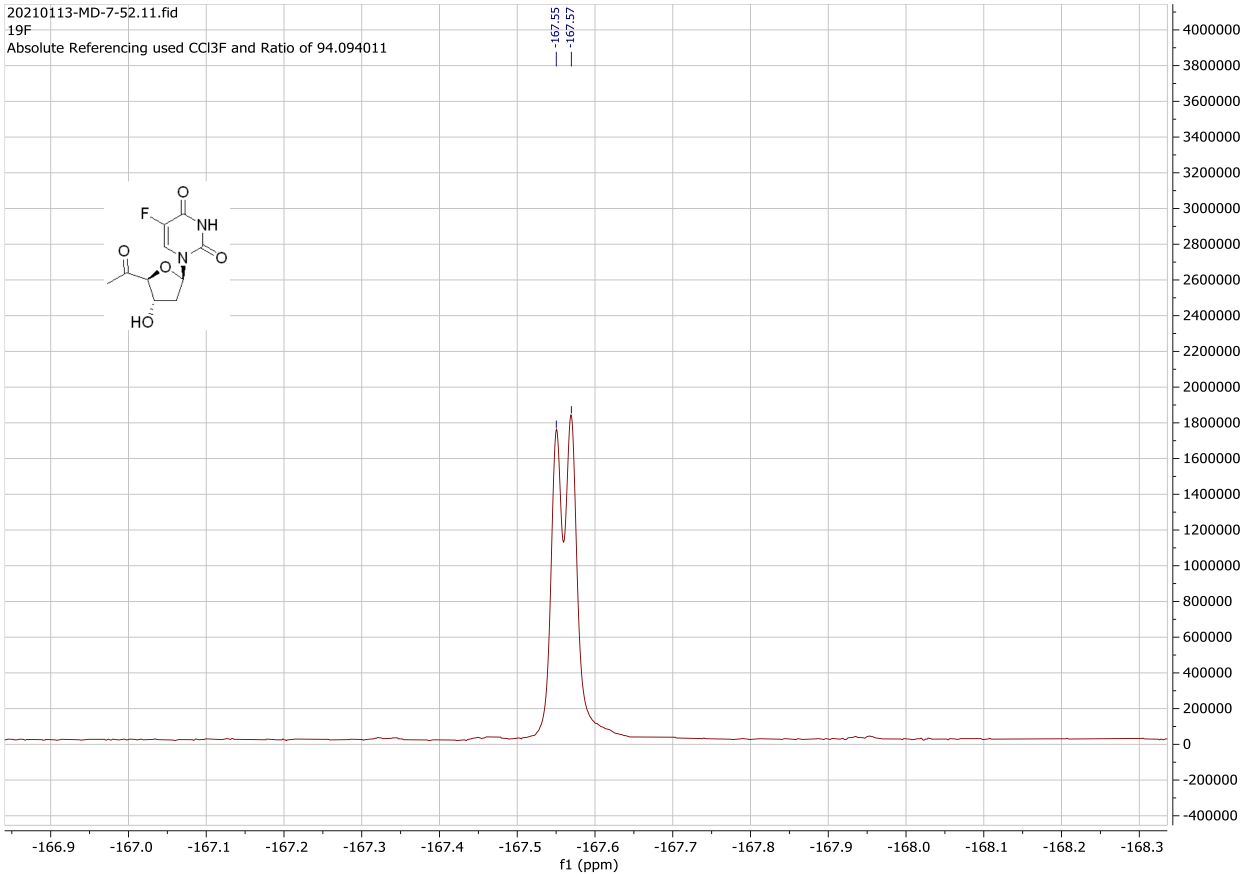
20210113-MD-7-52.11.fid
19F
Absolute Referencing used CCl3F and Ratio of 94.094011
-167.55
-167.57
f1 (ppm)

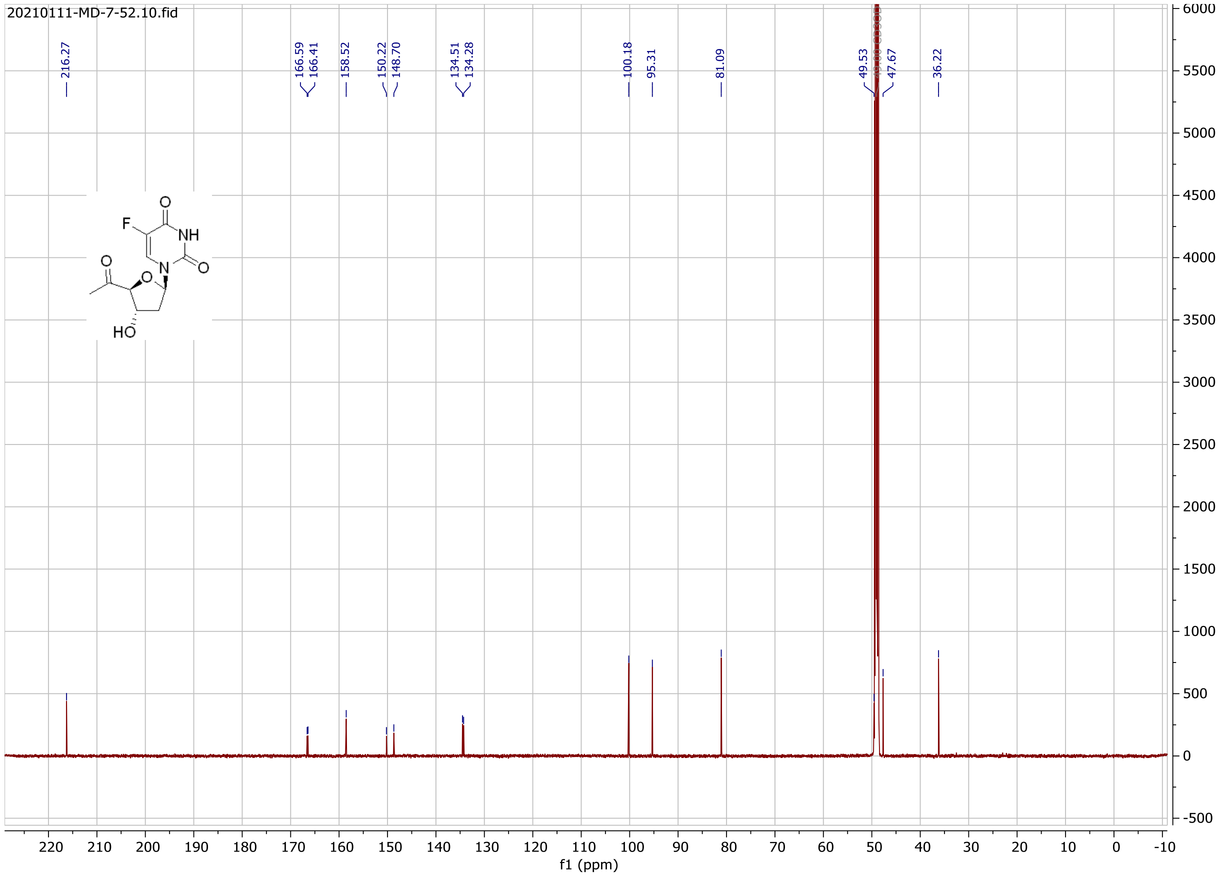
20210111-MD-7-52.10.fid
216.27
166.59
166.41
158.52
150.22
148.70
134.51
134.28
100.18
95.31
81.09
49.53
47.67
36.22
F
NH
O
N
HO
f1 (ppm)

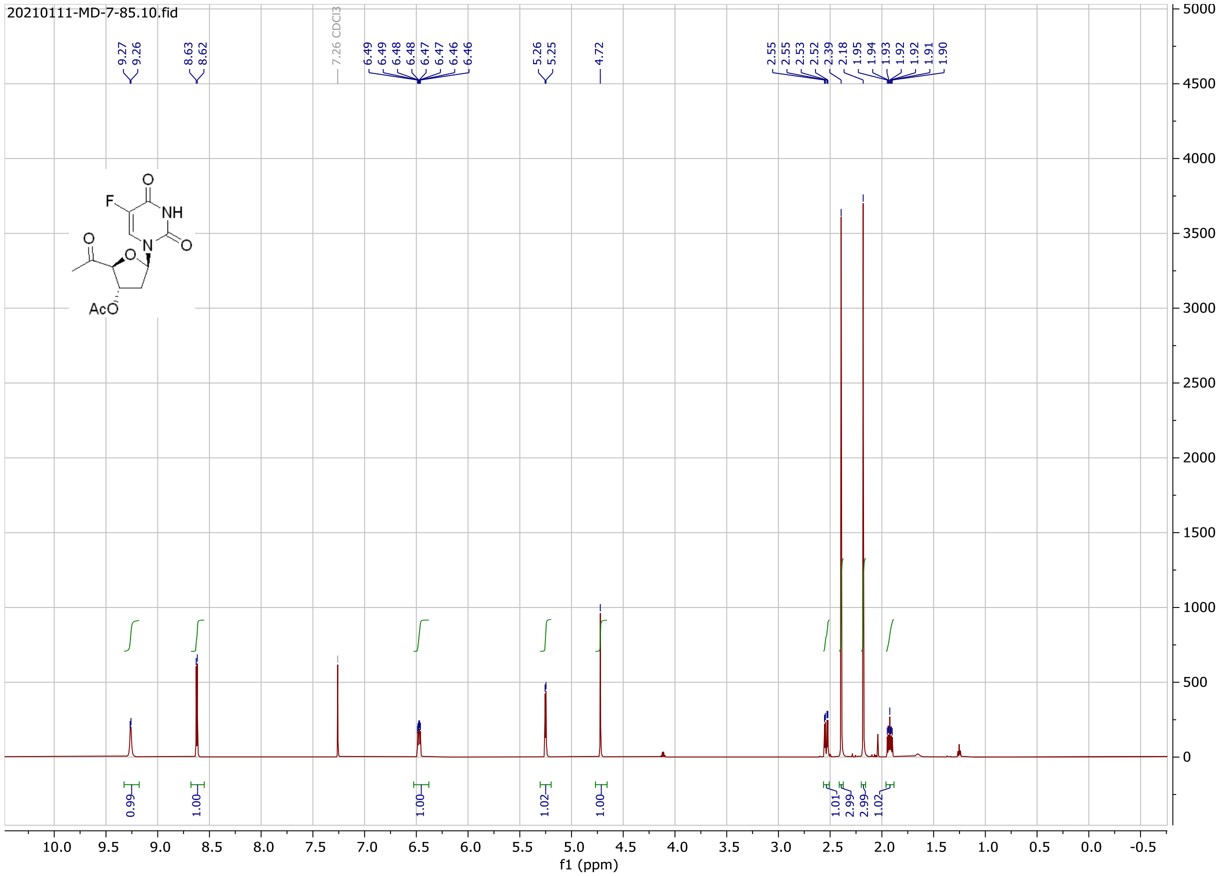

20210111-MD-7-85.10.fid
9.27
9.26
8.63
8.62
7.26 CDCl3
6.49
6.49
6.48
6.48
6.47
6.47
6.46
6.46
5.26
5.25
4.72
2.55
2.55
2.53
2.52
2.39
2.18
1.95
1.94
1.93
1.92
1.92
1.91
1.90
F
O
NH
N
O
O
O
AcO
0.99
1.00
1.00
1.02
1.00
1.01
2.99
2.99
1.02
f1 (ppm)

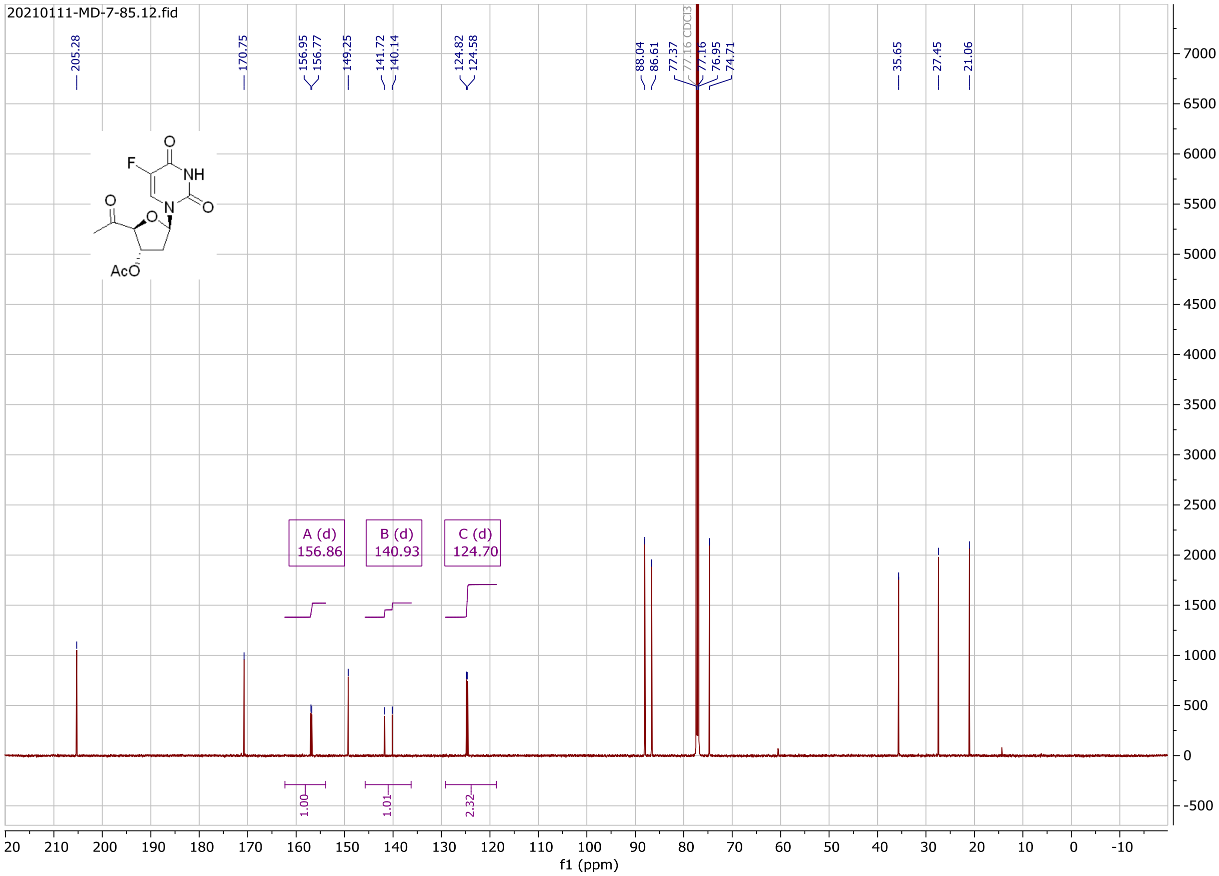
20210111-MD-7-85.12.fid
205.28
170.75
156.95
156.77
149.25
141.72
140.14
124.82
124.58
88.04
86.61
77.37
77.16 CDCl3
77.16
76.95
74.71
35.65
27.45
21.06
F
O
NH
N
O
O
O
AcO
A (d)
156.86
B (d)
140.93
C (d)
124.70
1.00
1.01
2.32
f1 (ppm)

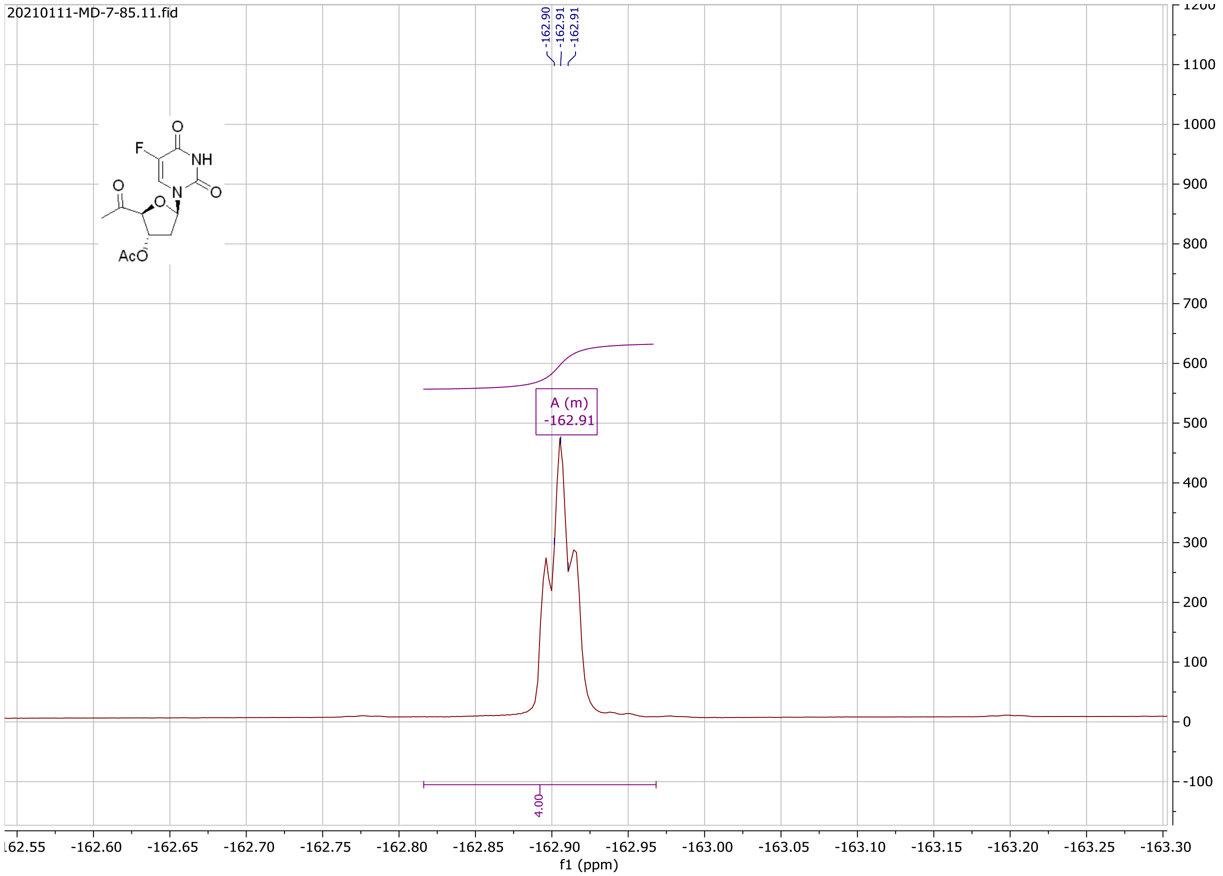

20210111-MD-7-85.11.fid
-162.90
-162.91
-162.91
F
O
NH
N
O
O
O
AcO
A (m)
-162.91
4.00
f1 (ppm)

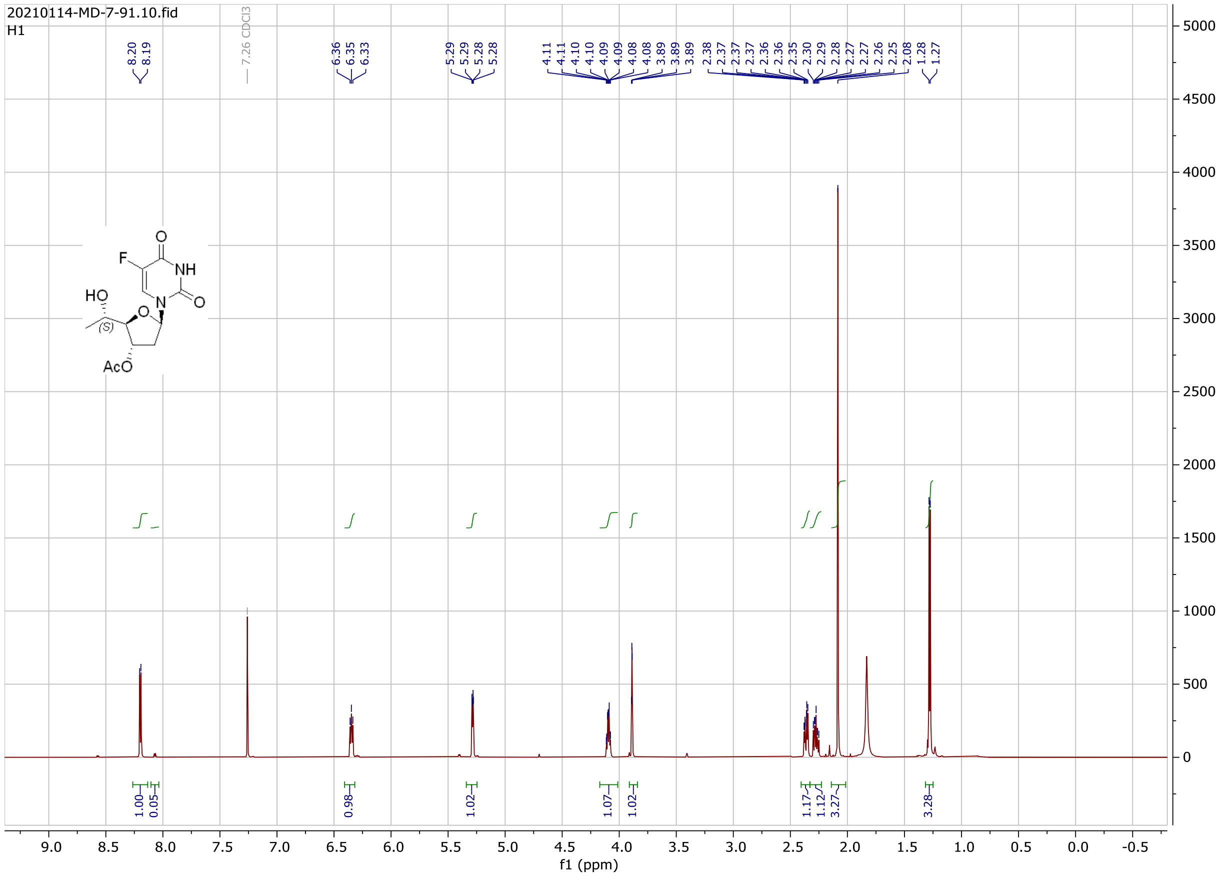
20210114-MD-7-91.10.fid
H1
8.20
8.19
7.26 CDCl3
6.36
6.35
6.33
5.29
5.29
5.28
5.28
4.11
4.11
4.10
4.10
4.09
4.09
4.08
4.08
3.89
3.89
3.89
2.38
2.37
2.37
2.37
2.36
2.36
2.35
2.30
2.29
2.28
2.28
2.27
2.27
2.26
2.25
2.08
1.28
1.27
F
O
NH
O
N
HO
O
(S)
AcO
1.00
0.05
0.98
1.02
1.07
1.02
1.17
1.12
3.27
3.28
f1 (ppm)

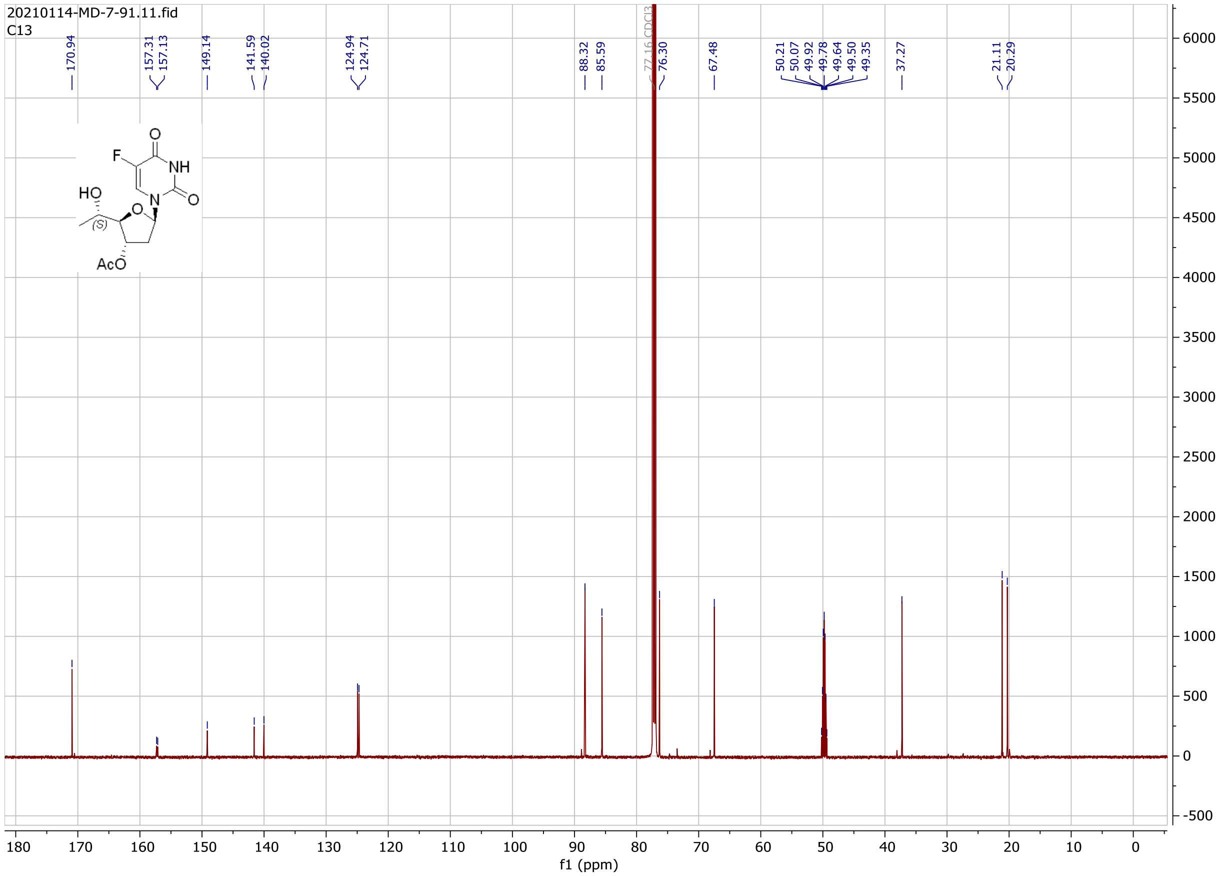
20210114-MD-7-91.11.fid
C13
170.94
157.31
157.13
149.14
141.59
140.02
124.94
124.71
88.32
85.59
77.16 CDCl3
76.30
67.48
50.21
50.07
49.92
49.78
49.64
49.50
49.35
37.27
21.11
20.29
O
F
NH
HO
N
O
O
(S)
AcO
f1 (ppm)

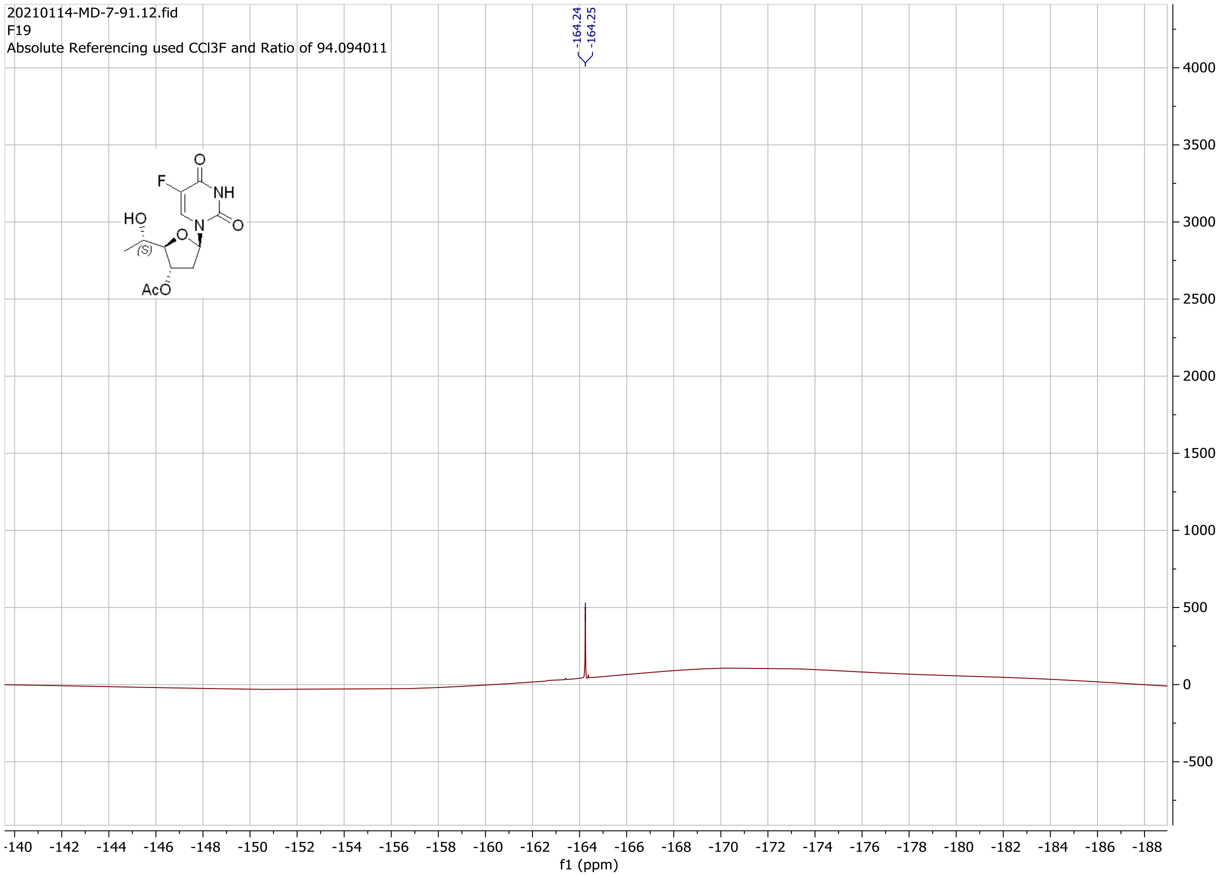
20210114-MD-7-91.12.fid
F19
Absolute Referencing used CCl3F and Ratio of 94.094011
-164.24
-164.25
F
O
NH
O
N
O
HO
(S)
AcO
f1 (ppm)

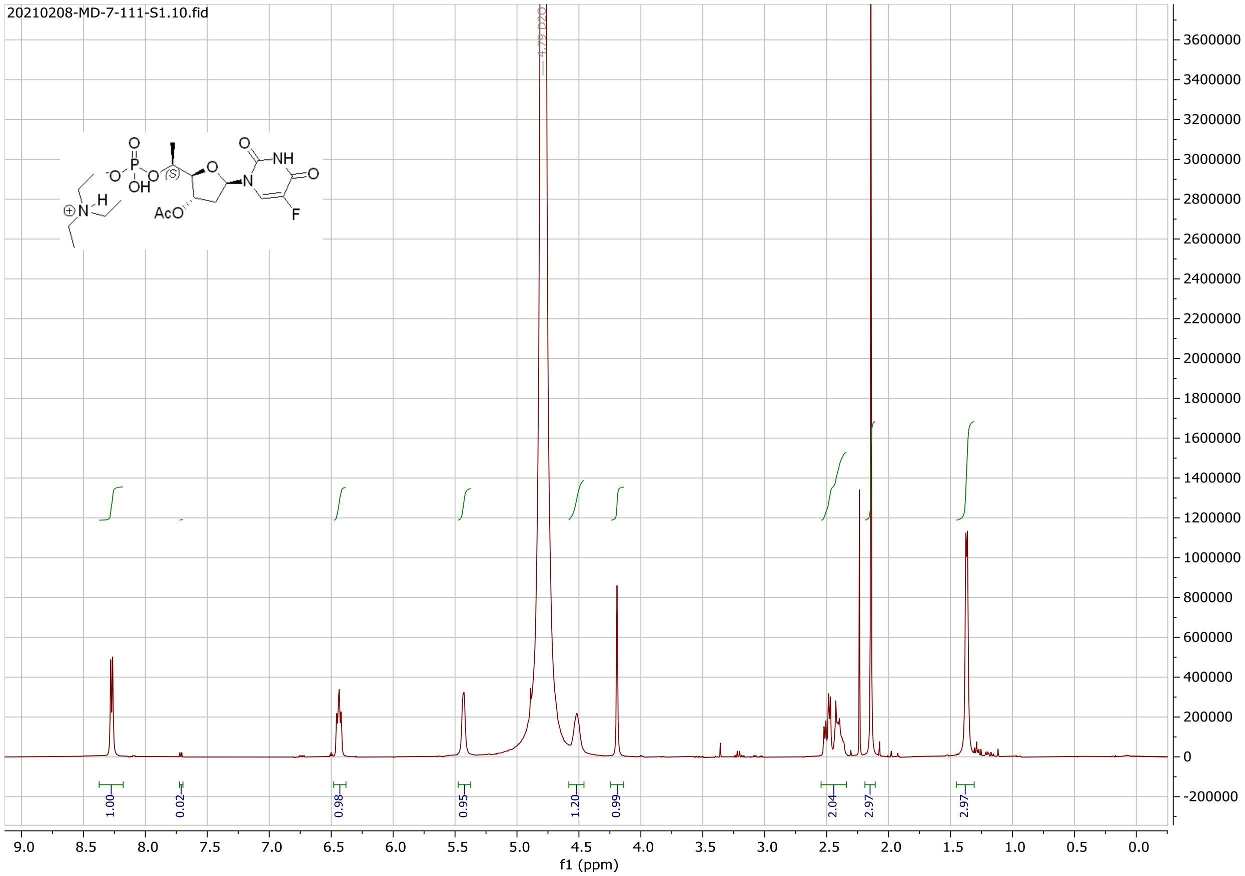

20210208-MD-7-111-S1.10.fid
4.79 D2O
AcO
F
NH
OH
H
1.00
0.02
0.98
0.95
1.20
0.99
2.04
2.97
2.97
f1 (ppm)

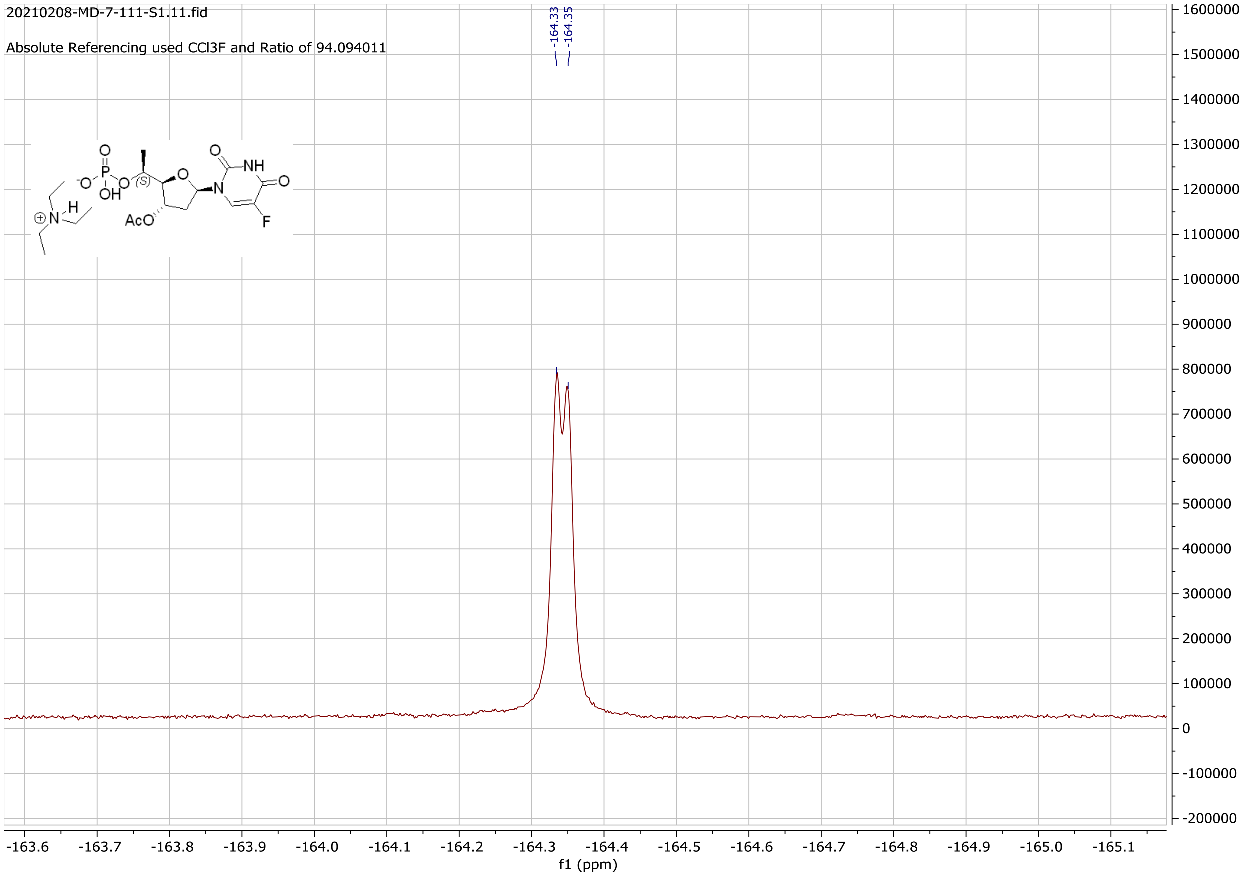

20210208-MD-7-111-S1.11.fid
Absolute Referencing used CCl3F and Ratio of 94.094011
-164.33
-164.35
f1 (ppm)

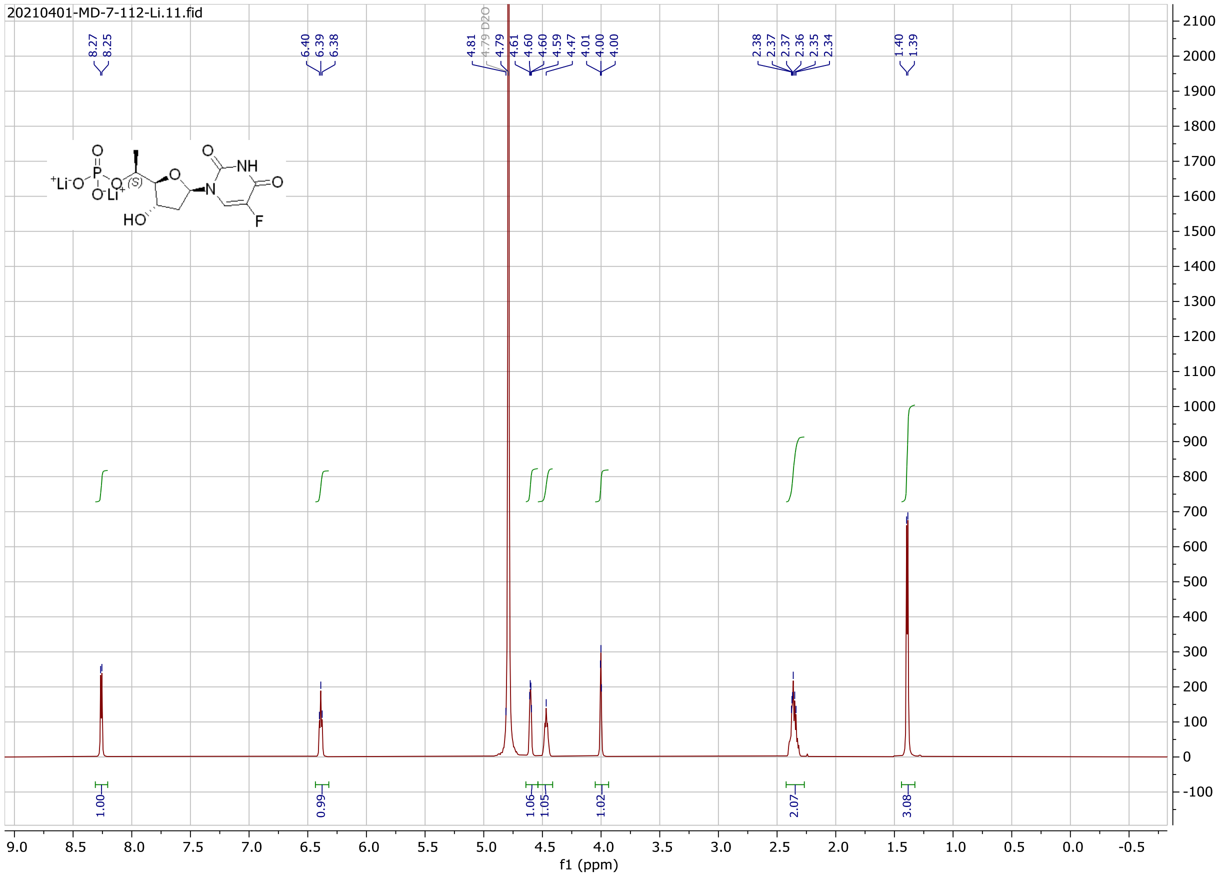
20210401-MD-7-112-Li.11.fid
8.27
8.25
6.40
6.39
6.38
4.81
4.79 D2O
4.79
4.61
4.60
4.60
4.59
4.47
4.01
4.00
4.00
2.38
2.37
2.37
2.36
2.35
2.34
1.40
1.39
1.00
0.99
1.06
1.05
1.02
2.07
3.08
f1 (ppm)
(S)
HO
F
NH
O
N
P
$^{+}$Li $^{-}$O
O^{-} Li^{+}

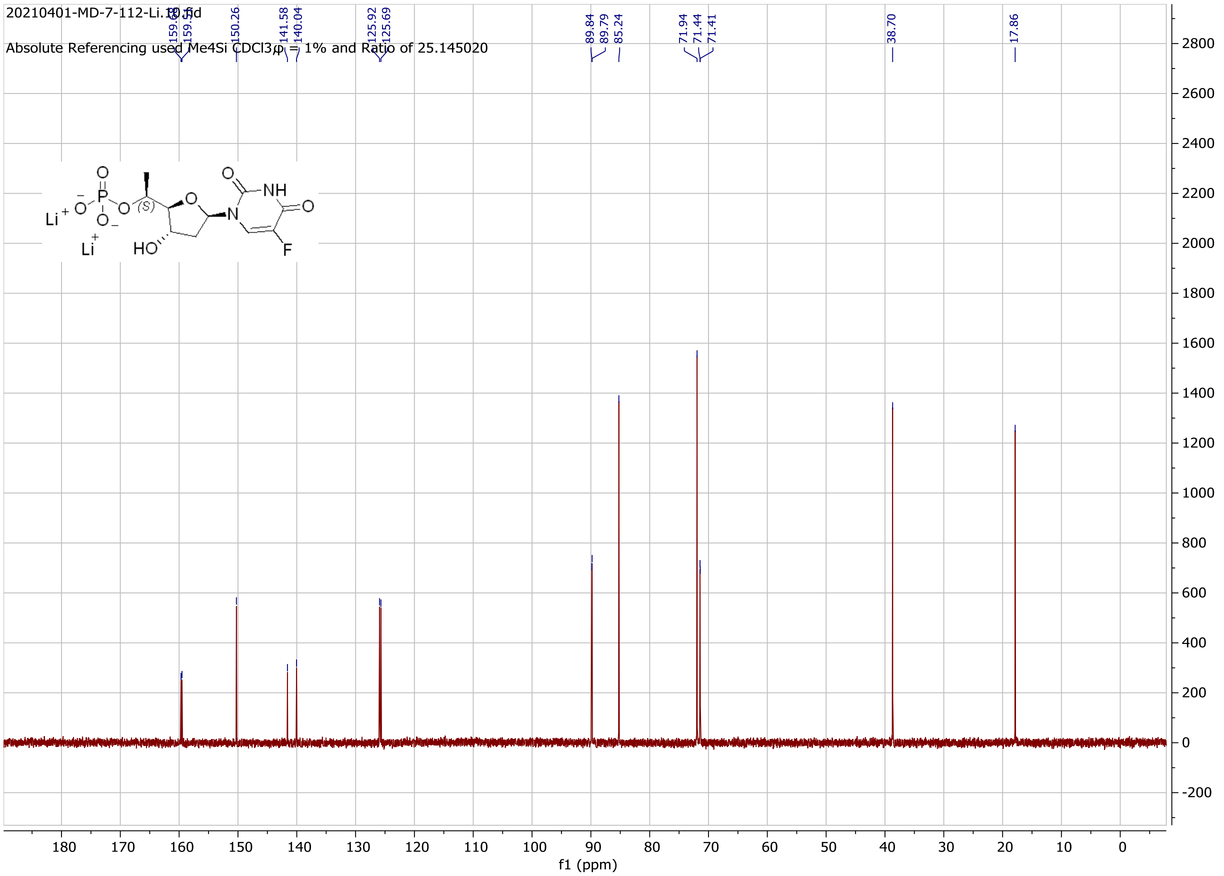
Absolute Referencing used Me4Si CDCl3 p = 1% and Ratio of 25.145020
150.26
141.58
140.04
125.92
125.69
89.84
89.79
85.24
71.94
71.44
71.41
38.70
17.86
f1 (ppm)

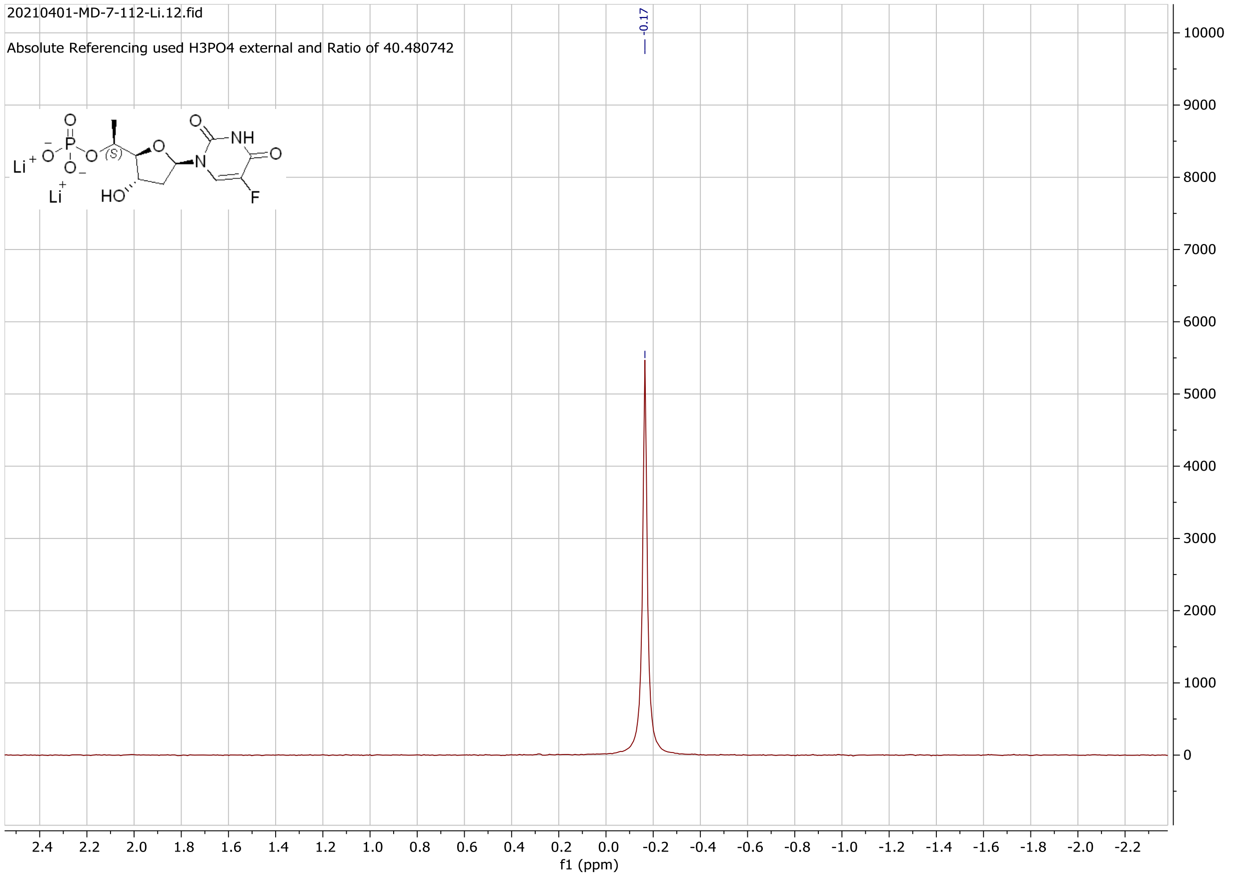

20210401-MD-7-112-Li.12.fid
Absolute Referencing used H3PO4 external and Ratio of 40.480742
-0.17
Li+
Li+
HO
NH
F
(S)
f1 (ppm)

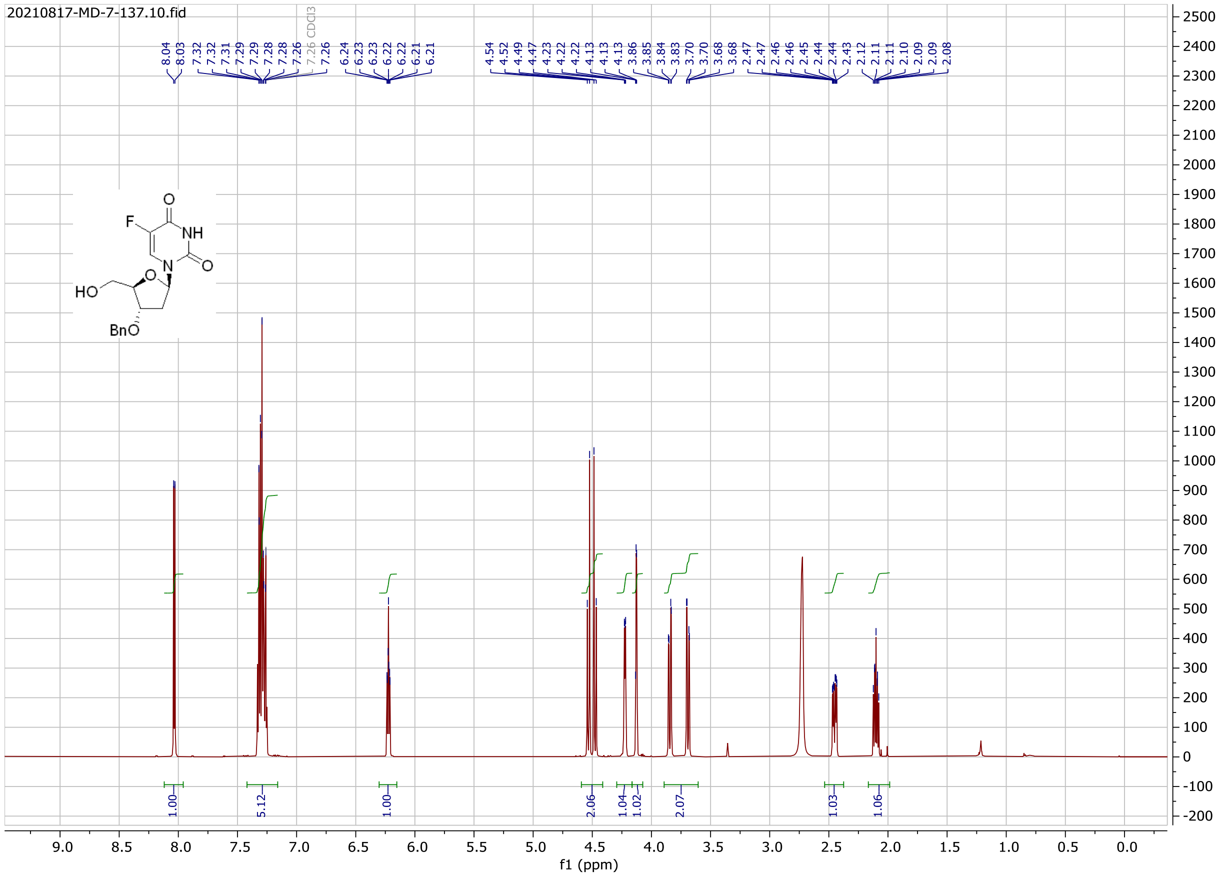
20210817-MD-7-137.10.fid
8.04
8.03
7.32
7.32
7.31
7.29
7.29
7.28
7.28
7.26 CDCl3
7.26
6.24
6.23
6.23
6.22
6.22
6.21
6.21
4.54
4.52
4.49
4.47
4.23
4.22
4.22
4.13
4.13
4.13
3.86
3.85
3.84
3.83
3.70
3.70
3.68
3.68
2.47
2.46
2.46
2.45
2.44
2.44
2.43
2.12
2.11
2.11
2.10
2.09
2.09
2.08
F
O
NH
N
O
HO
O
BnO
1.00
5.12
1.00
2.06
1.04
1.02
2.07
1.03
1.06
f1 (ppm)

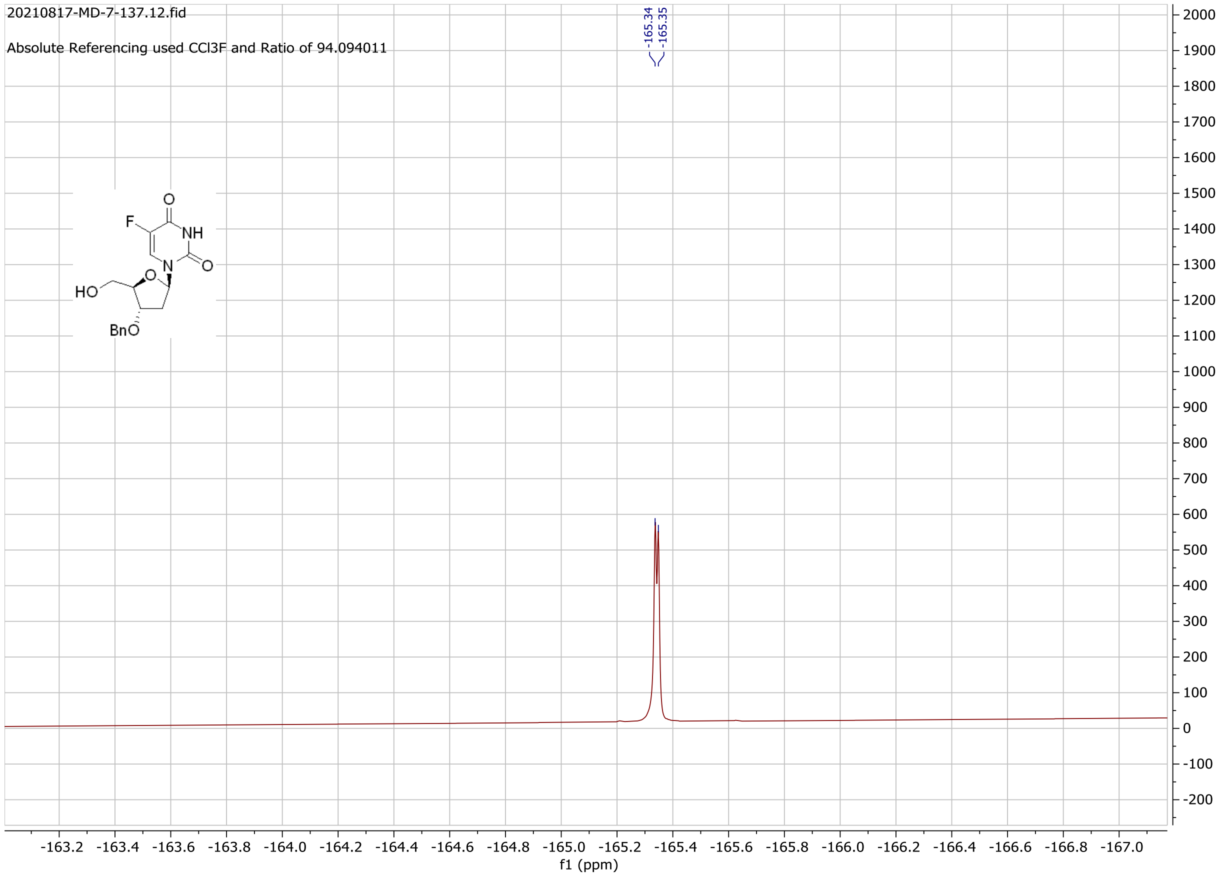

20210817-MD-7-137.12.fid
Absolute Referencing used CCl3F and Ratio of 94.094011
-165.34
-165.35
F
O
NH
N
O
HO
O
BnO
2000
1900
1800
1700
1600
1500
1400
1300
1200
1100
1000
900
800
700
600
500
400
300
200
100
0
-100
-200
-163.2 -163.4 -163.6 -163.8 -164.0 -164.2 -164.4 -164.6 -164.8 -165.0 -165.2 -165.4 -165.6 -165.8 -166.0 -166.2 -166.4 -166.6 -166.8 -167.0
f1 (ppm)

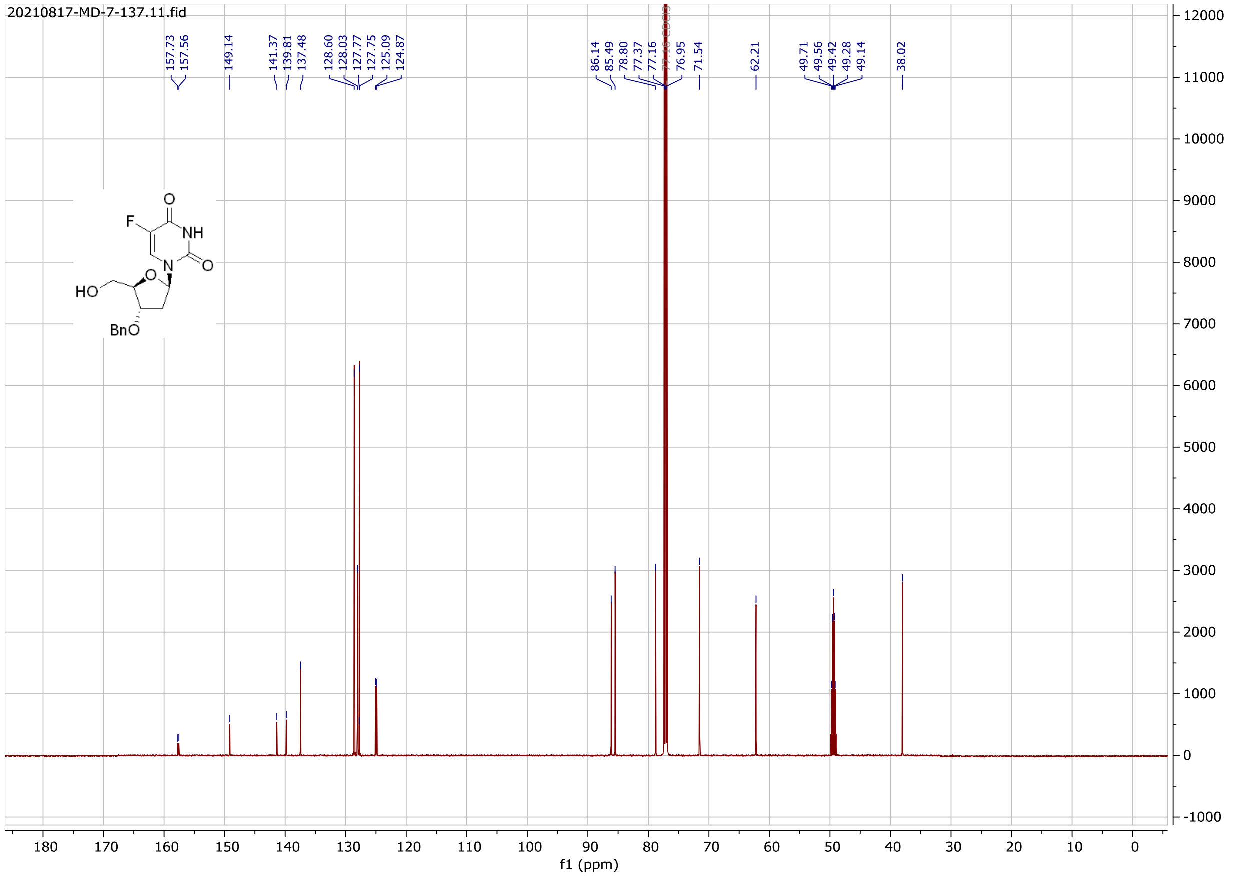
20210817-MD-7-137.11.fid
157.73
157.56
149.14
141.37
139.81
137.48
128.60
128.03
127.77
127.75
125.09
124.87
86.14
85.49
78.80
77.37
77.16
76.95
71.54
62.21
49.71
49.56
49.42
49.28
49.14
38.02
F
O
NH
N
O
HO
O
BnO
f1 (ppm)

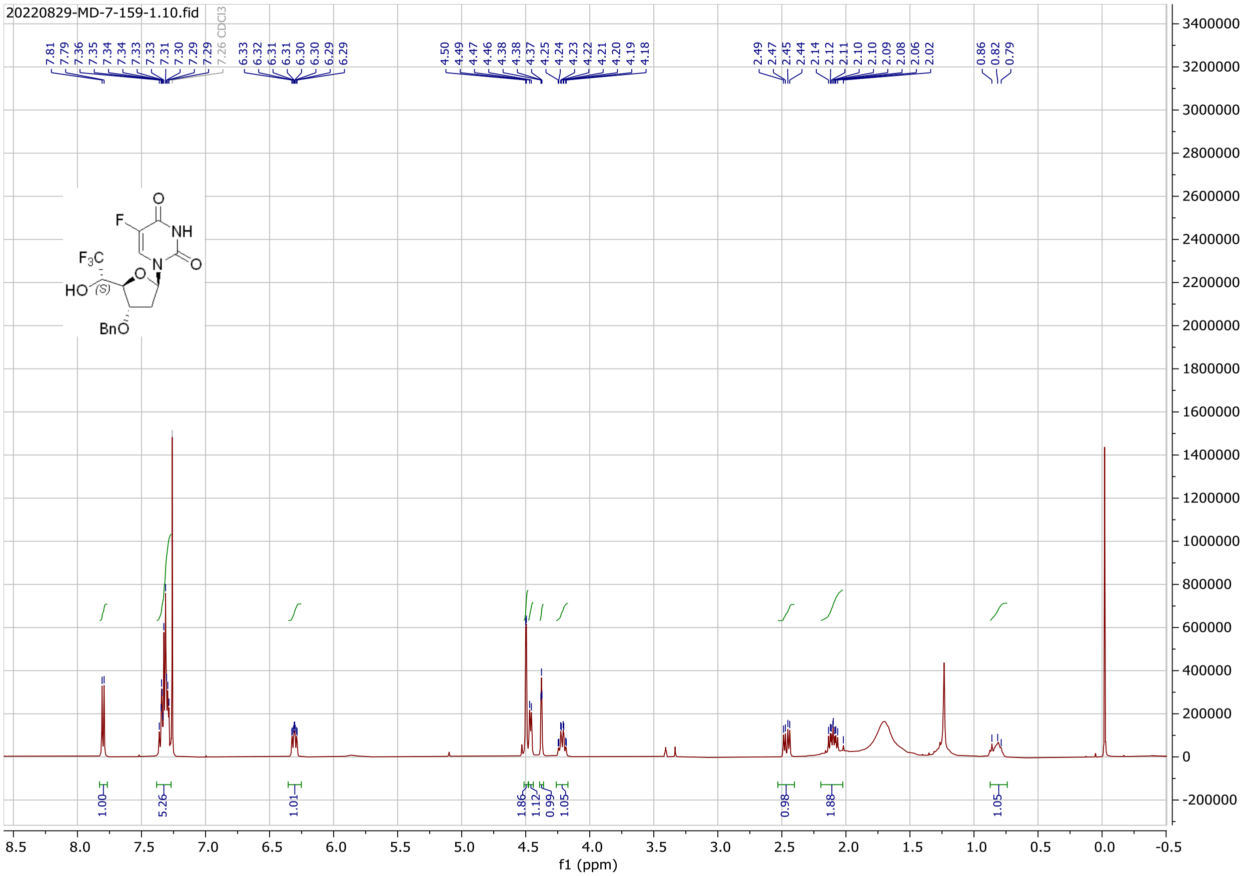
20220829-MD-7-159-1.10.fid
f1 (ppm)
F
NH
O
N
O
F3C
HO
(S)
BnO
7.26 CDCl3

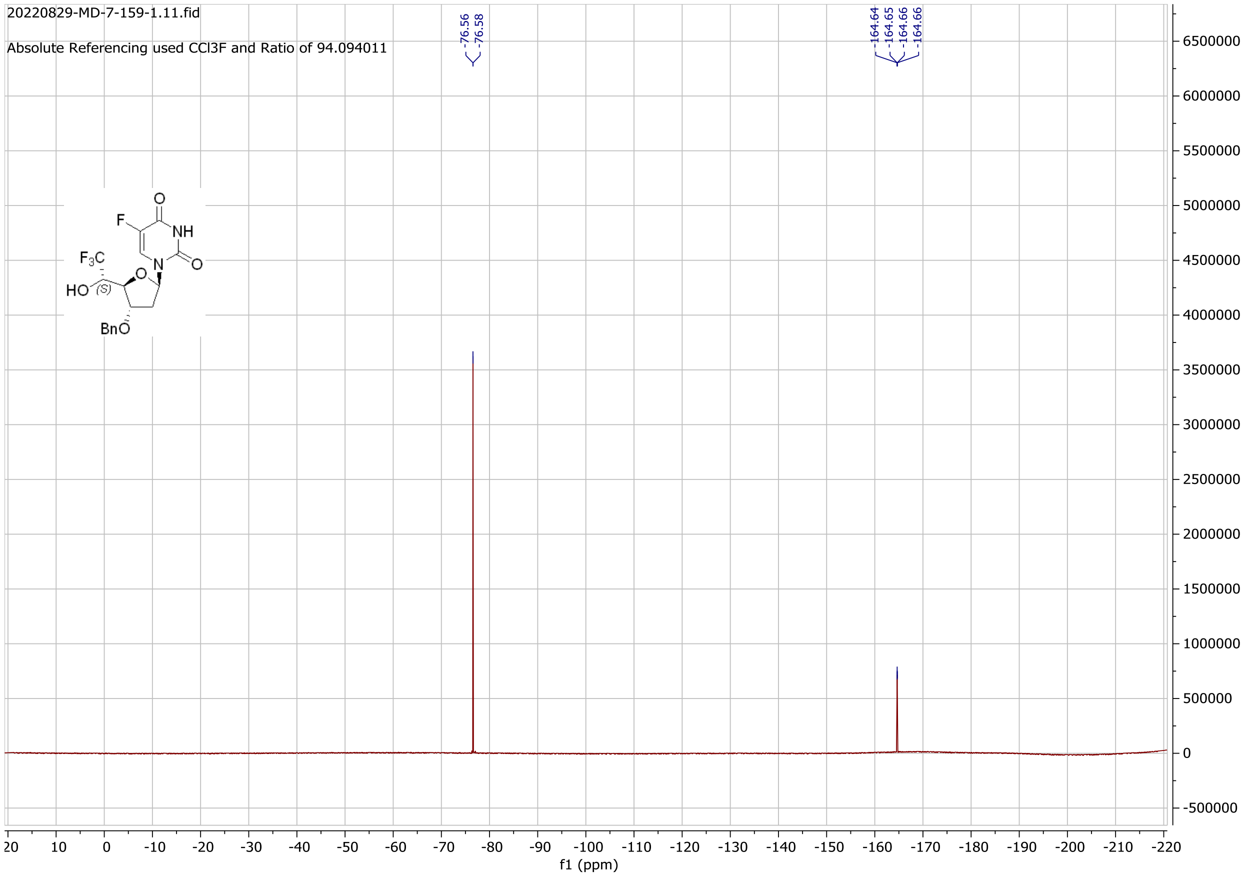
20220829-MD-7-159-1.11.fid
Absolute Referencing used CCl3F and Ratio of 94.094011
-76.56
-76.58
-164.64
-164.65
-164.66
-164.66
F
O
NH
N
O
F3C
HO
(S)
O
BnO
6500000
6000000
5500000
5000000
4500000
4000000
3500000
3000000
2500000
2000000
1500000
1000000
500000
0
-500000
20 10 0 -10 -20 -30 -40 -50 -60 -70 -80 -90 -100 -110 -120 -130 -140 -150 -160 -170 -180 -190 -200 -210 -220
f1 (ppm)

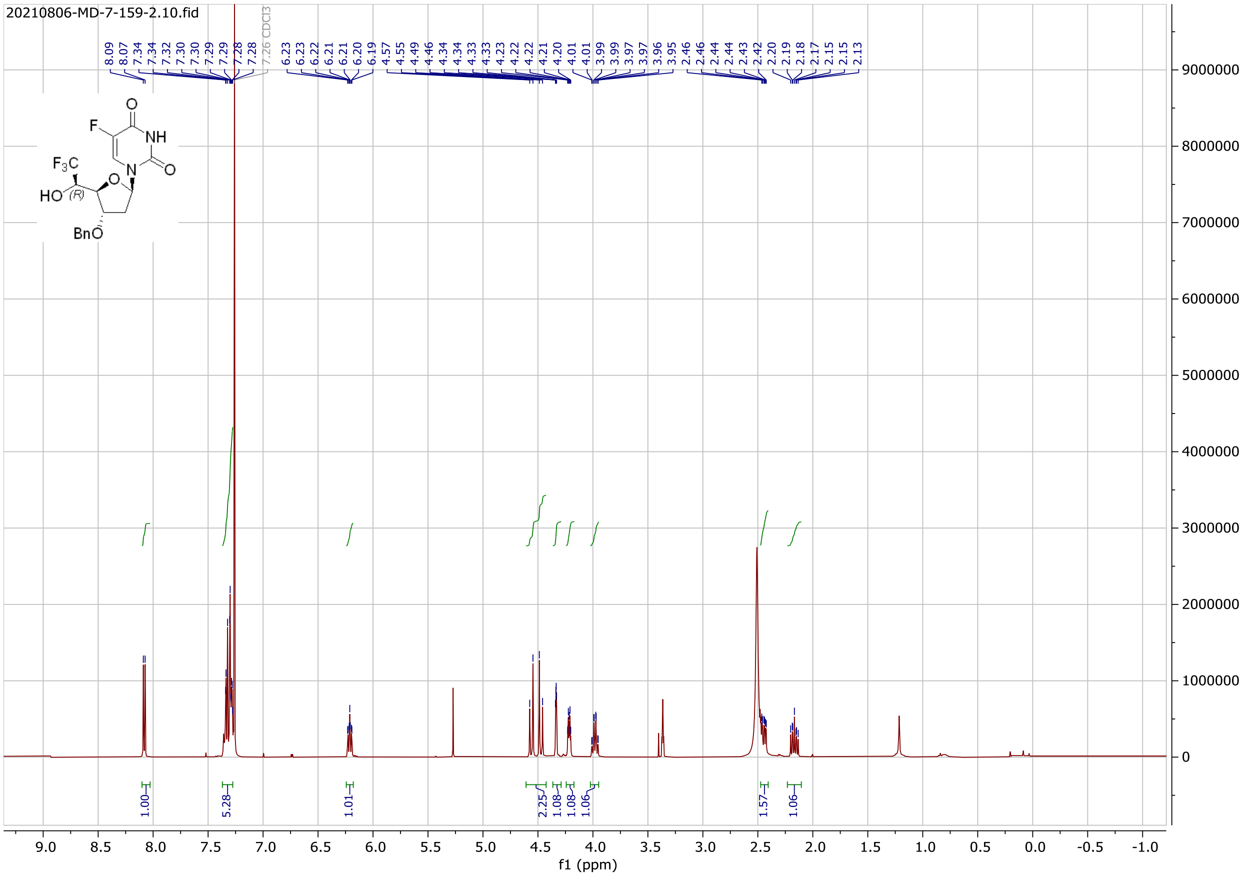
20210806-MD-7-159-2.10.fid
8.09 8.07 7.34 7.34 7.32 7.30 7.30 7.29 7.29 7.28 7.28
7.26 CDCl3
6.23 6.23 6.22 6.21 6.21 6.20 6.19
4.57 4.55 4.49 4.46 4.34 4.34 4.33 4.33 4.23 4.22 4.22 4.21 4.20 4.01 4.01 3.99 3.99 3.97 3.97 3.96 3.95
2.46 2.44 2.44 2.43 2.42 2.20 2.19 2.18 2.17 2.15 2.13
F
O
NH
N
O
F3C
O
HO
(R)
BnO
1.00
5.28
1.01
2.25
1.08
1.08
1.06
1.57
1.06
9000000
8000000
7000000
6000000
5000000
4000000
3000000
2000000
1000000
0
9.0 8.5 8.0 7.5 7.0 6.5 6.0 5.5 5.0 4.5 4.0 3.5 3.0 2.5 2.0 1.5 1.0 0.5 0.0 -0.5 -1.0
f1 (ppm)

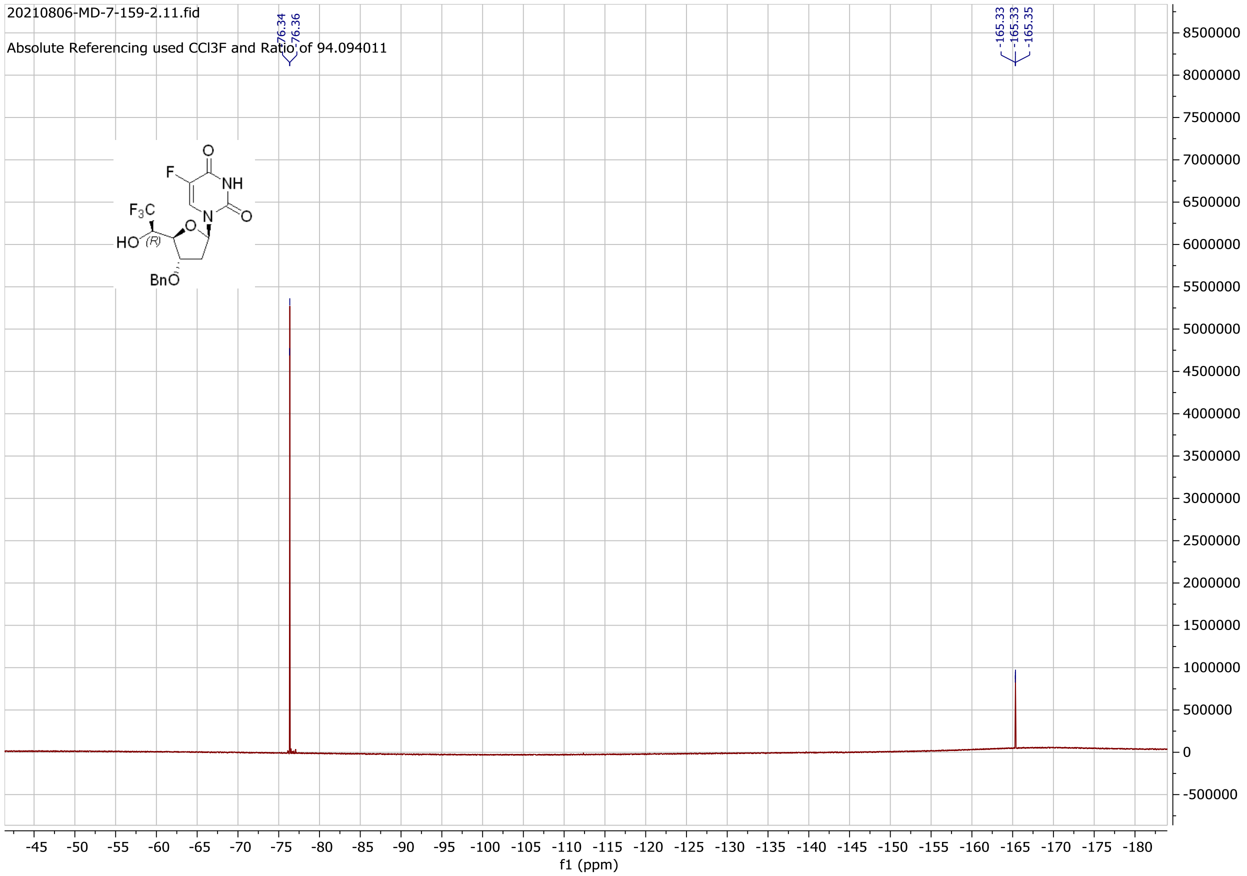
20210806-MD-7-159-2.11.fid
Absolute Referencing used CCl3F and Ratio of 94.094011
-76.34
-76.36
-165.33
-165.33
-165.35
F
O
NH
O
N
F3C
O
HO
(R)
BnO
f1 (ppm)

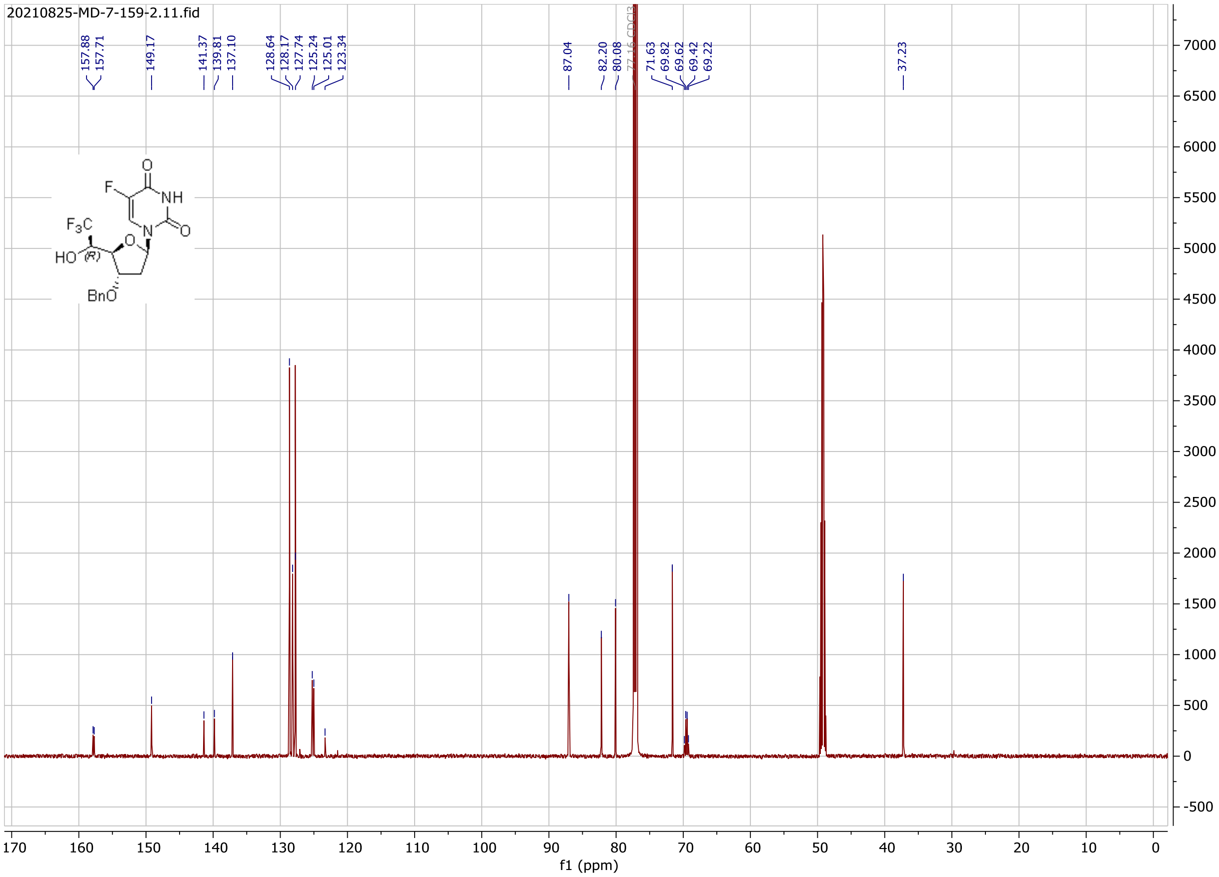
20210825-MD-7-159-2.11.fid
157.88
157.71
149.17
141.37
139.81
137.10
128.64
128.17
127.74
125.24
125.01
123.34
87.04
82.20
80.08
77.16 CDCl3
71.63
69.82
69.62
69.42
69.22
37.23
F
O
NH
O
N
F3C
O
HO
(R)
BnO
f1 (ppm)

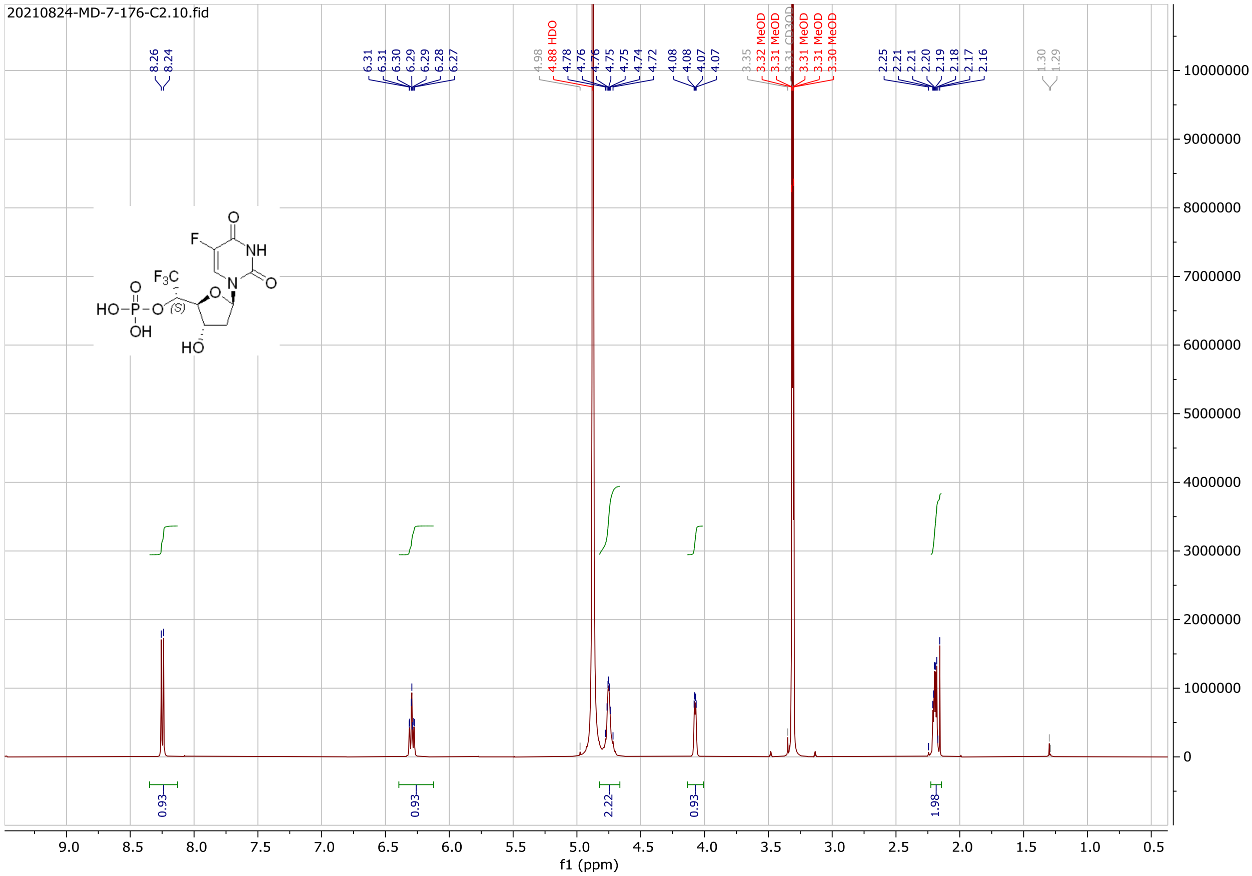

20210824-MD-7-176-C2.10.fid
8.26
8.24
6.31
6.31
6.30
6.29
6.29
6.28
6.27
4.98
4.88 HDO
4.78
4.76
4.76
4.75
4.75
4.74
4.72
4.08
4.08
4.07
4.07
3.35
3.32 MeOD
3.31 MeOD
3.31 CD3OD
3.31 MeOD
3.31 MeOD
3.30 MeOD
2.25
2.21
2.21
2.20
2.19
2.18
2.17
2.16
1.30
1.29
0.93
0.93
2.22
0.93
1.98
f1 (ppm)

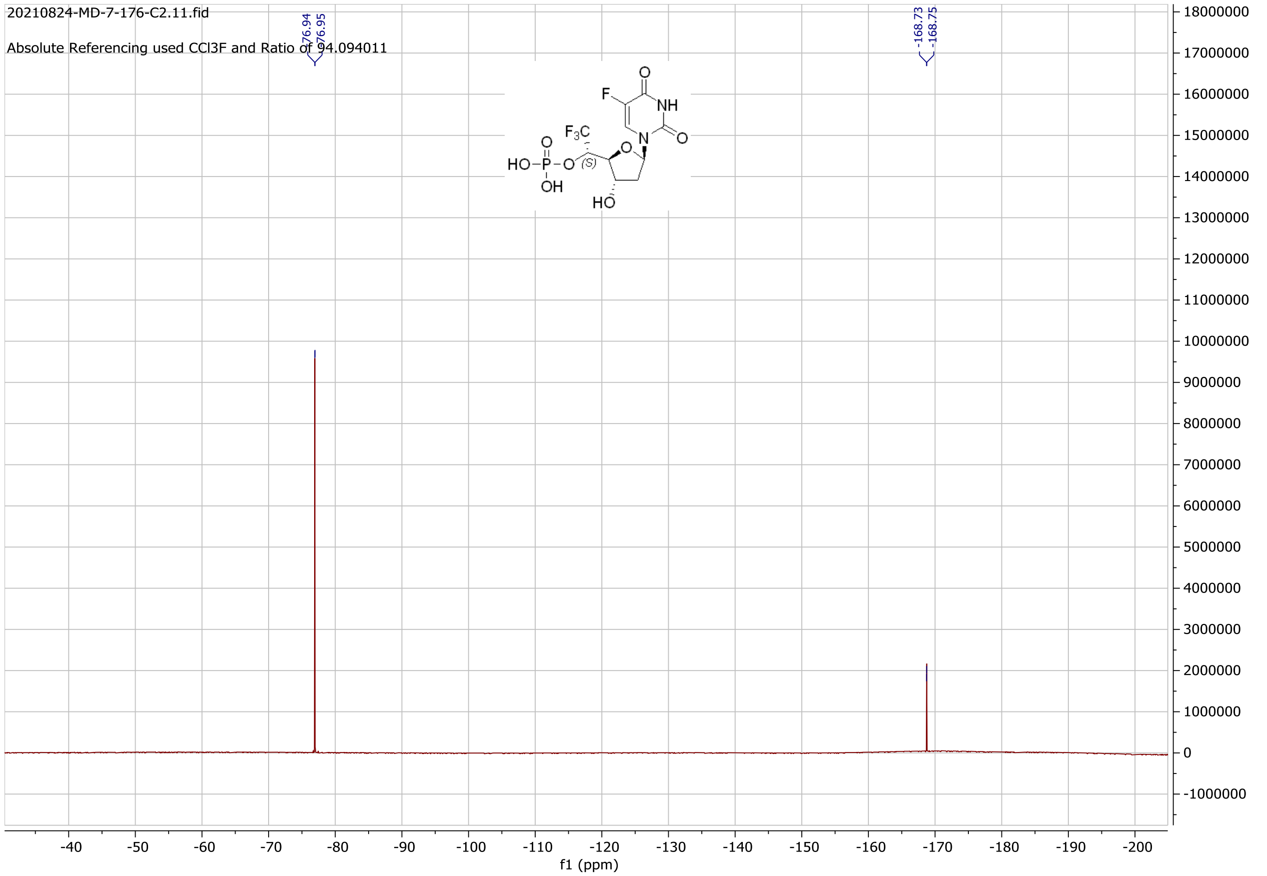

20210824-MD-7-176-C2.11.fid
Absolute Referencing used CCl3F and Ratio of 94.094011
-76.94
-76.95
-168.73
-168.75
f1 (ppm)

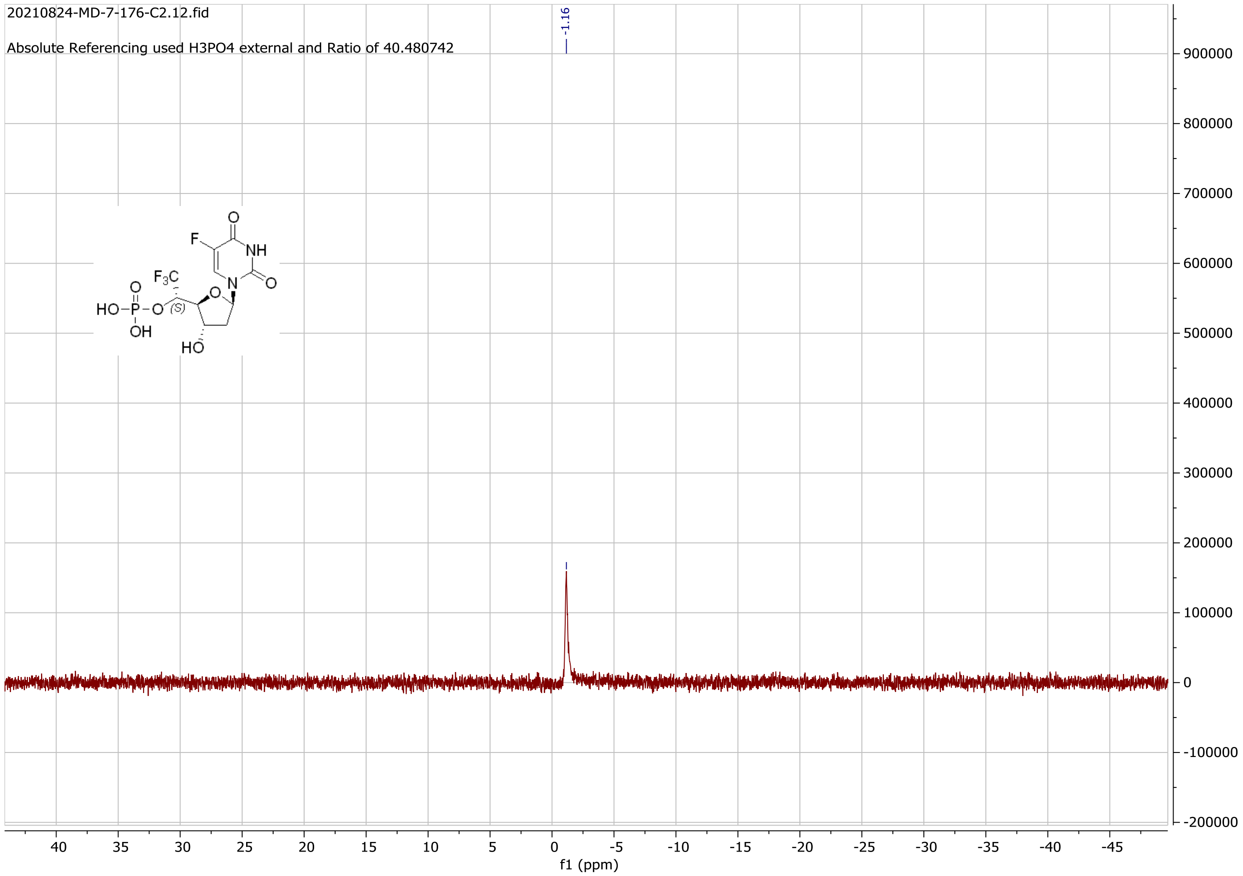
20210824-MD-7-176-C2.12.fid
Absolute Referencing used H3PO4 external and Ratio of 40.480742
-1.16
F
O
NH
N
O
F3C
O
HO-P-O
(S)
O
OH
HO
900000
800000
700000
600000
500000
400000
300000
200000
100000
0
-100000
-200000
40 35 30 25 20 15 10 5 0 -5 -10 -15 -20 -25 -30 -35 -40 -45
f1 (ppm)

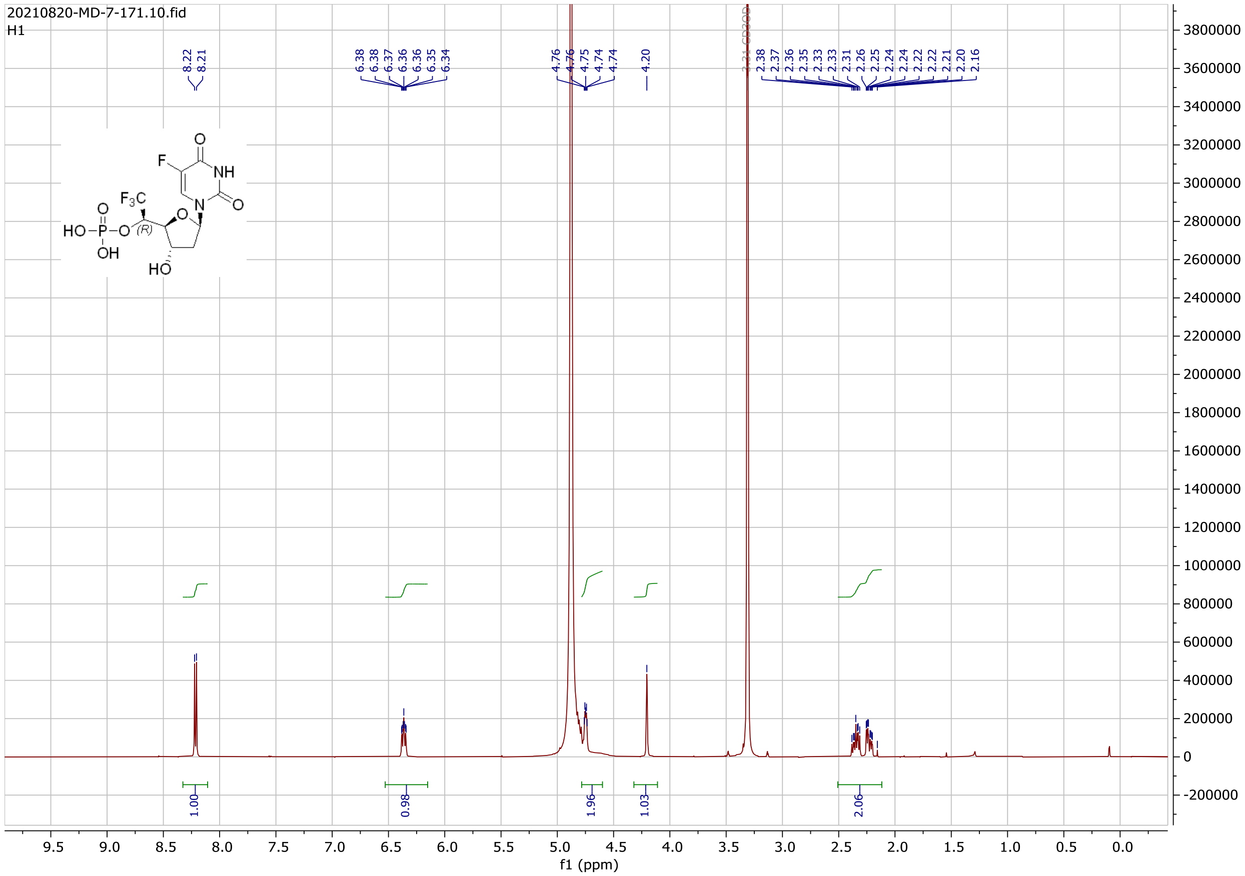

20210820-MD-7-171.10.fid
H1
8.22
8.21
6.38
6.38
6.37
6.36
6.36
6.35
6.34
4.76
4.76
4.75
4.74
4.74
4.20
2.38
2.37
2.36
2.35
2.33
2.33
2.31
2.26
2.25
2.24
2.24
2.22
2.22
2.21
2.20
2.16
F
O
NH
N
O
F_3C
HO
P
O
OH
(R)
HO
1.00
0.98
1.96
1.03
2.06
f1 (ppm)

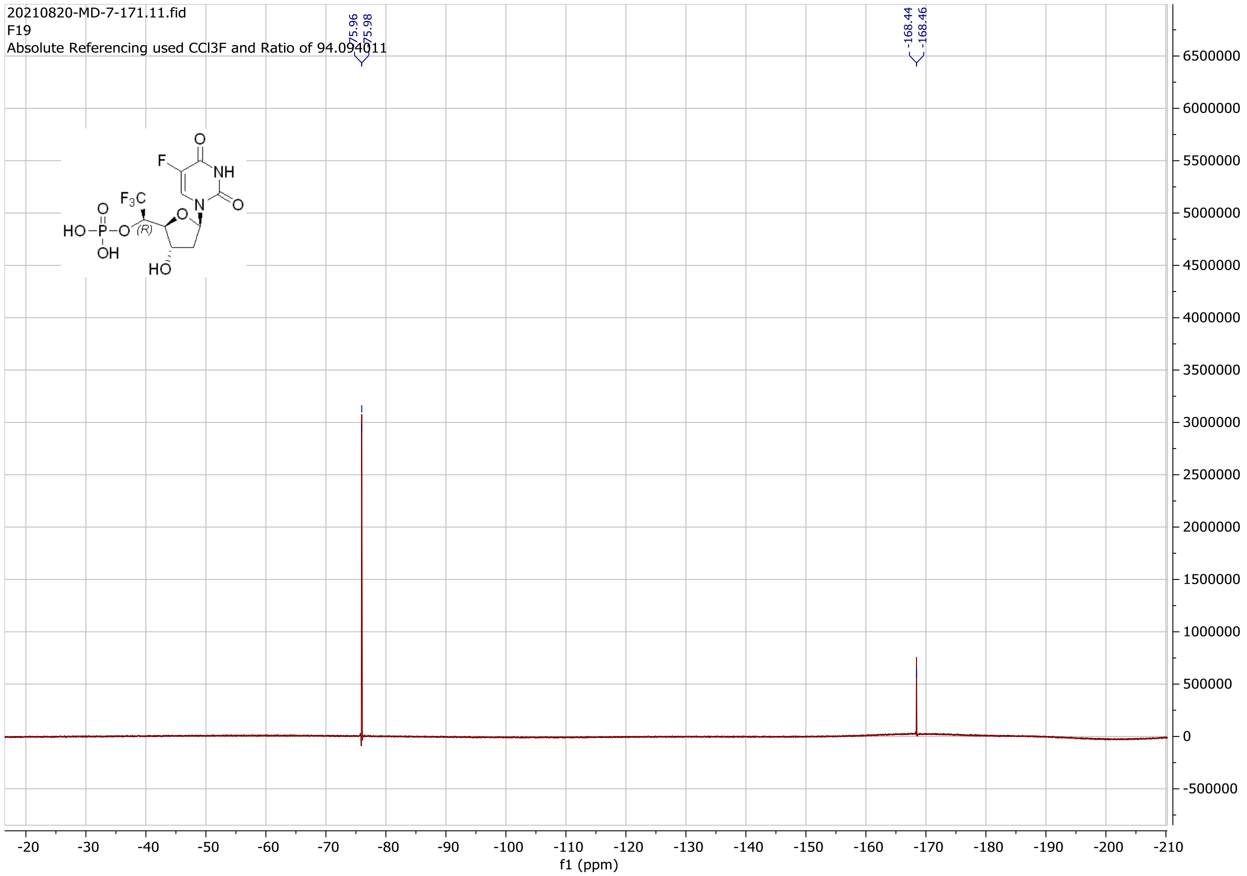
20210820-MD-7-171.11.fid
F19
Absolute Referencing used CCl3F and Ratio of 94.094011
-75.96
-75.98
-168.44
-168.46
F
O
NH
O
N
O
F3C
O
HO-P-O
(R)
OH
HO
f1 (ppm)

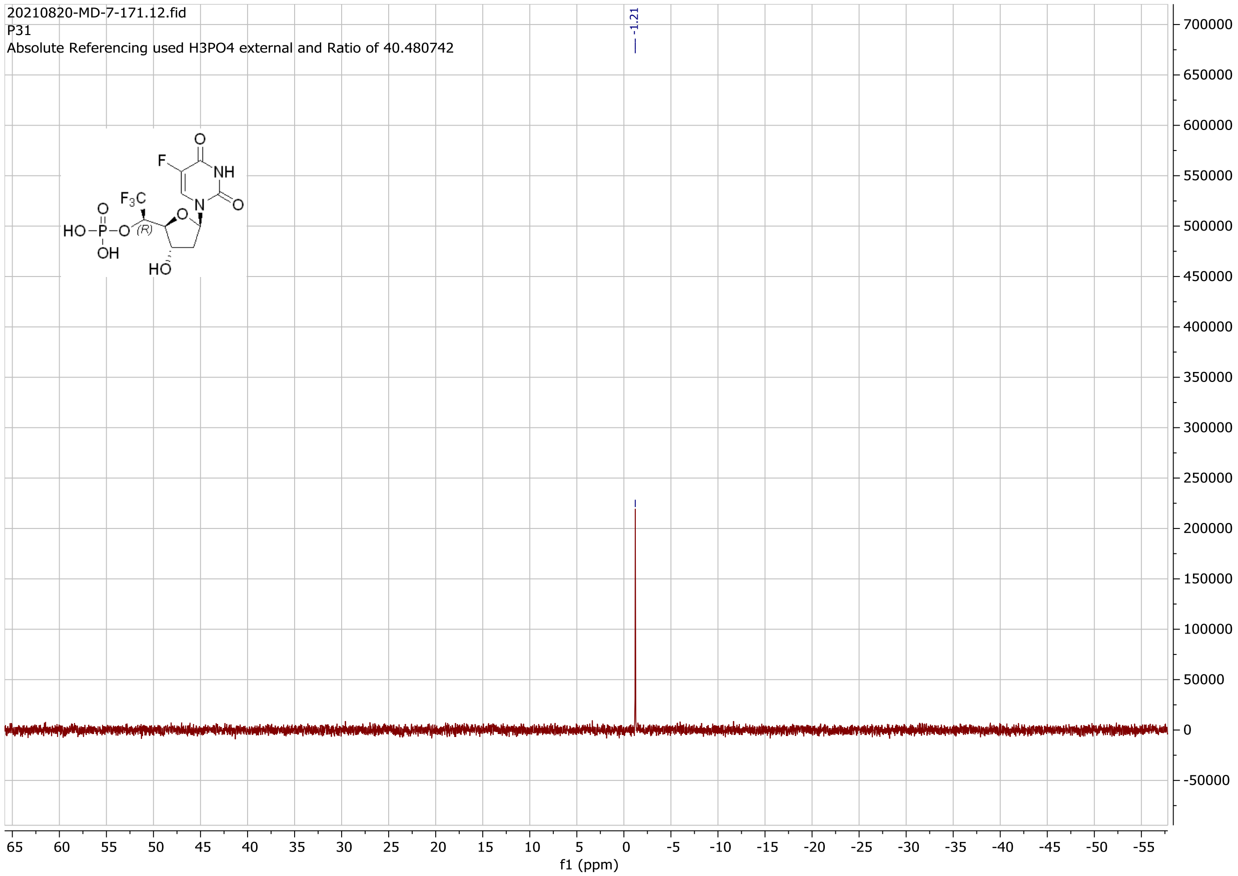
20210820-MD-7-171.12.fid
P31
Absolute Referencing used H3PO4 external and Ratio of 40.480742
-1.21
f1 (ppm)

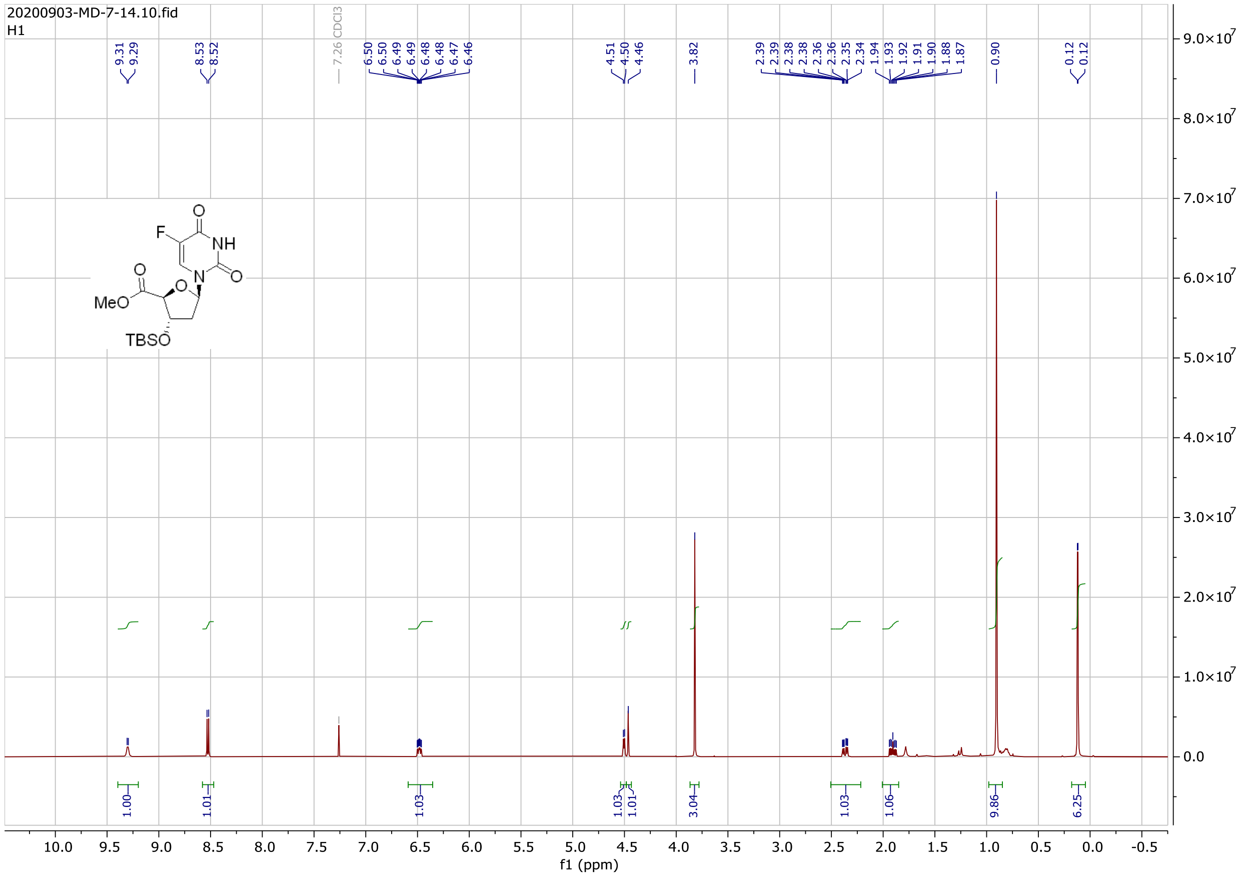

20200903-MD-7-14.10.fid
H1
f1 (ppm)

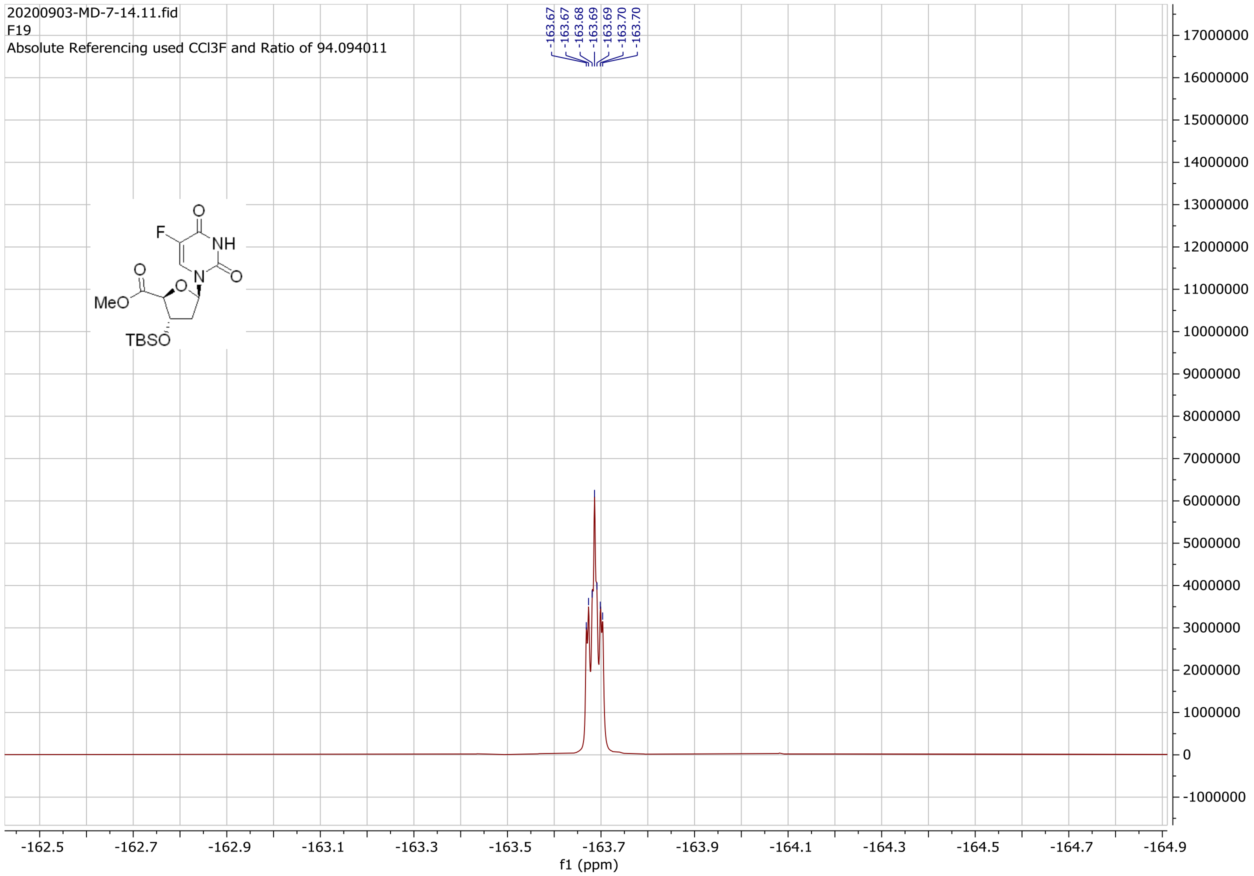
20200903-MD-7-14.11.fid
F19
Absolute Referencing used CCl3F and Ratio of 94.094011
-163.67
-163.67
-163.68
-163.69
-163.69
-163.70
-163.70
F
O
NH
N
O
O
MeO
O
TBSO
17000000
16000000
15000000
14000000
13000000
12000000
11000000
10000000
9000000
8000000
7000000
6000000
5000000
4000000
3000000
2000000
1000000
0
-1000000
-162.5
-162.7
-162.9
-163.1
-163.3
-163.5
-163.7
-163.9
-164.1
-164.3
-164.5
-164.7
-164.9
f1 (ppm)

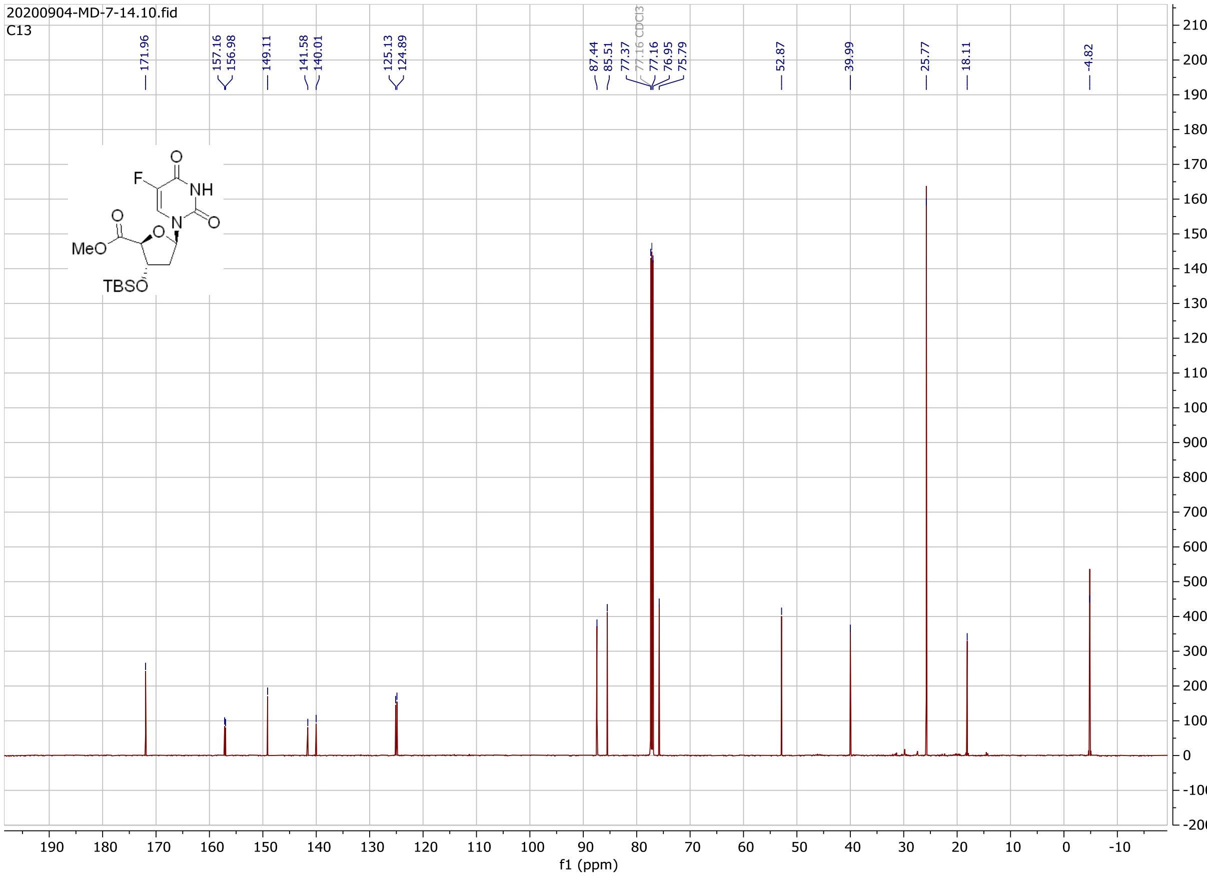
20200904-MD-7-14.10.fid
C13
171.96
157.16
156.98
149.11
141.58
140.01
125.13
124.89
87.44
85.51
77.37
77.16 CDCl3
77.16
76.95
75.79
52.87
39.99
25.77
18.11
-4.82
F
O
NH
N
O
O
MeO
O
TBSO
f1 (ppm)

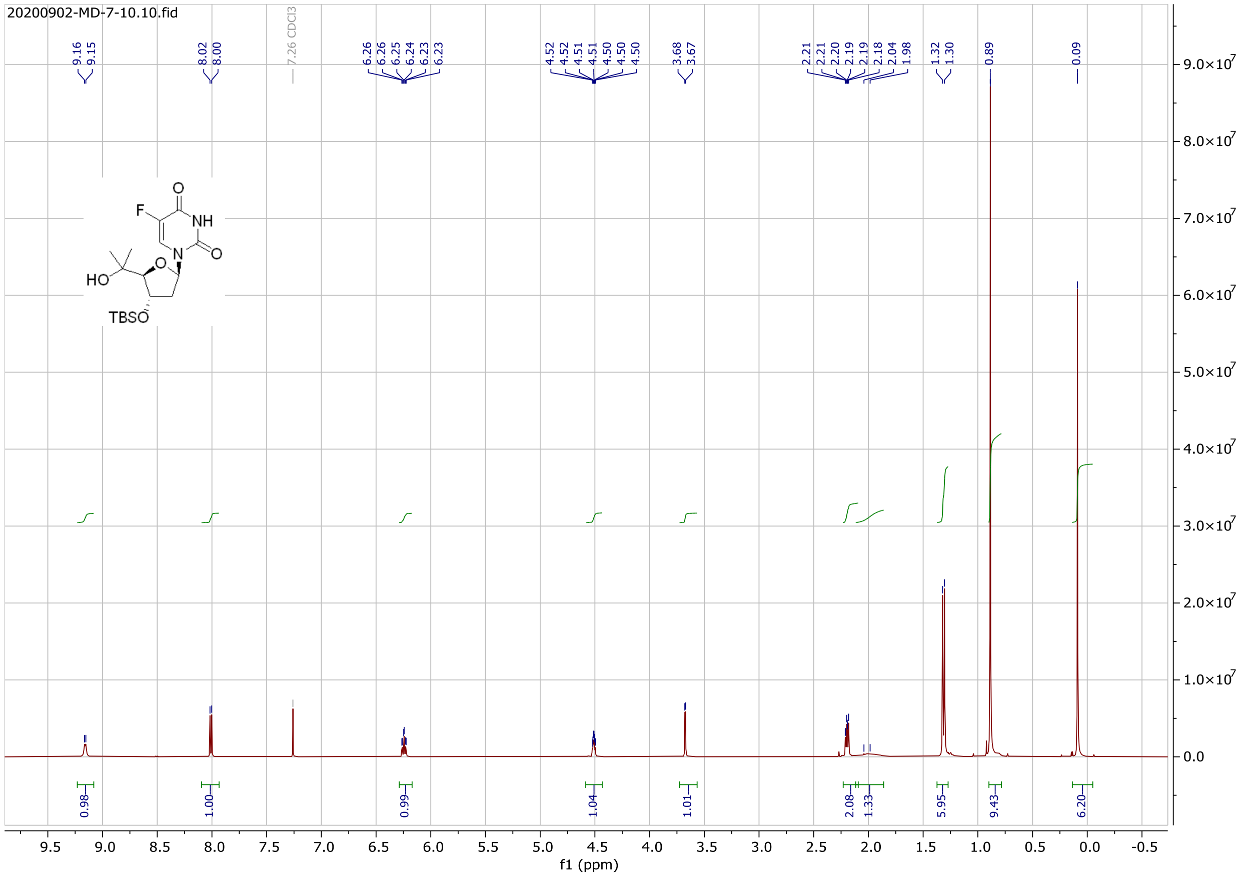
20200902-MD-7-10.10.fid
f1 (ppm)
F
O
NH
O
N
O
HO
TBSO

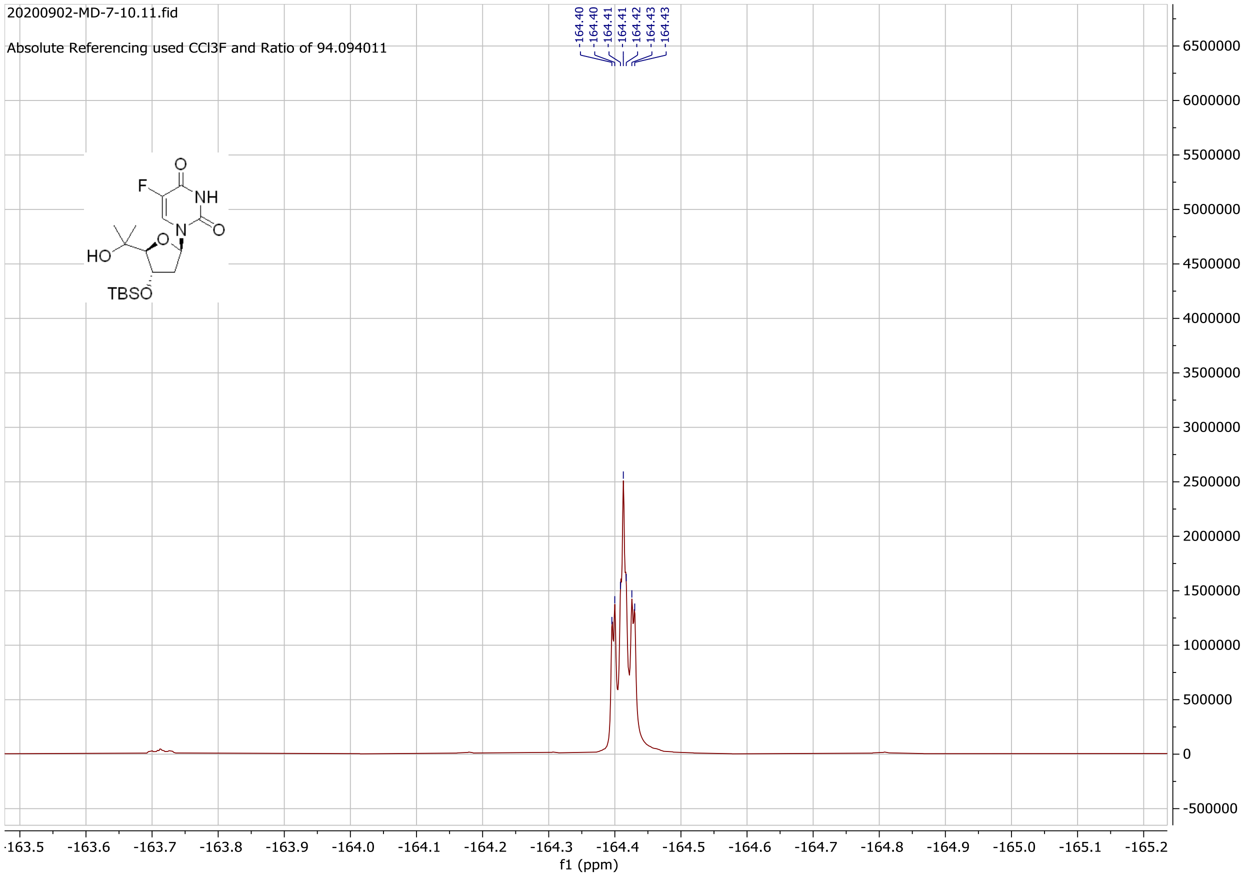

20200902-MD-7-10.11.fid
Absolute Referencing used CCl3F and Ratio of 94.094011
-164.40
-164.40
-164.41
-164.41
-164.42
-164.43
-164.43
F
O
NH
N
O
O
HO
TBSO
f1 (ppm)

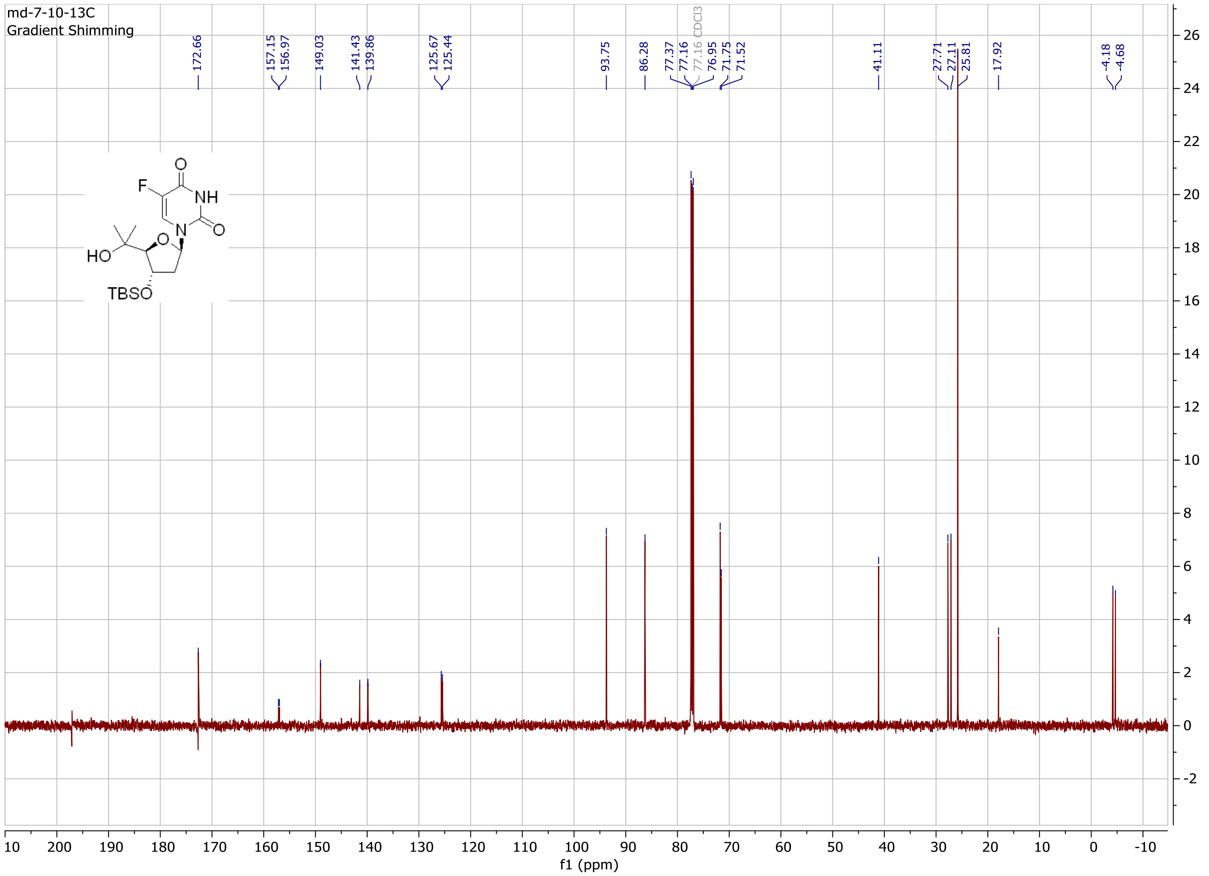
md-7-10-13C
Gradient Shimming
172.66
157.15
156.97
149.03
141.43
139.86
125.67
125.44
93.75
86.28
77.37
77.16
77.16 CDCl3
76.95
71.75
71.52
41.11
27.71
27.11
25.81
17.92
-4.18
-4.68
F
O
NH
N
O
HO
O
TBSO
f1 (ppm)

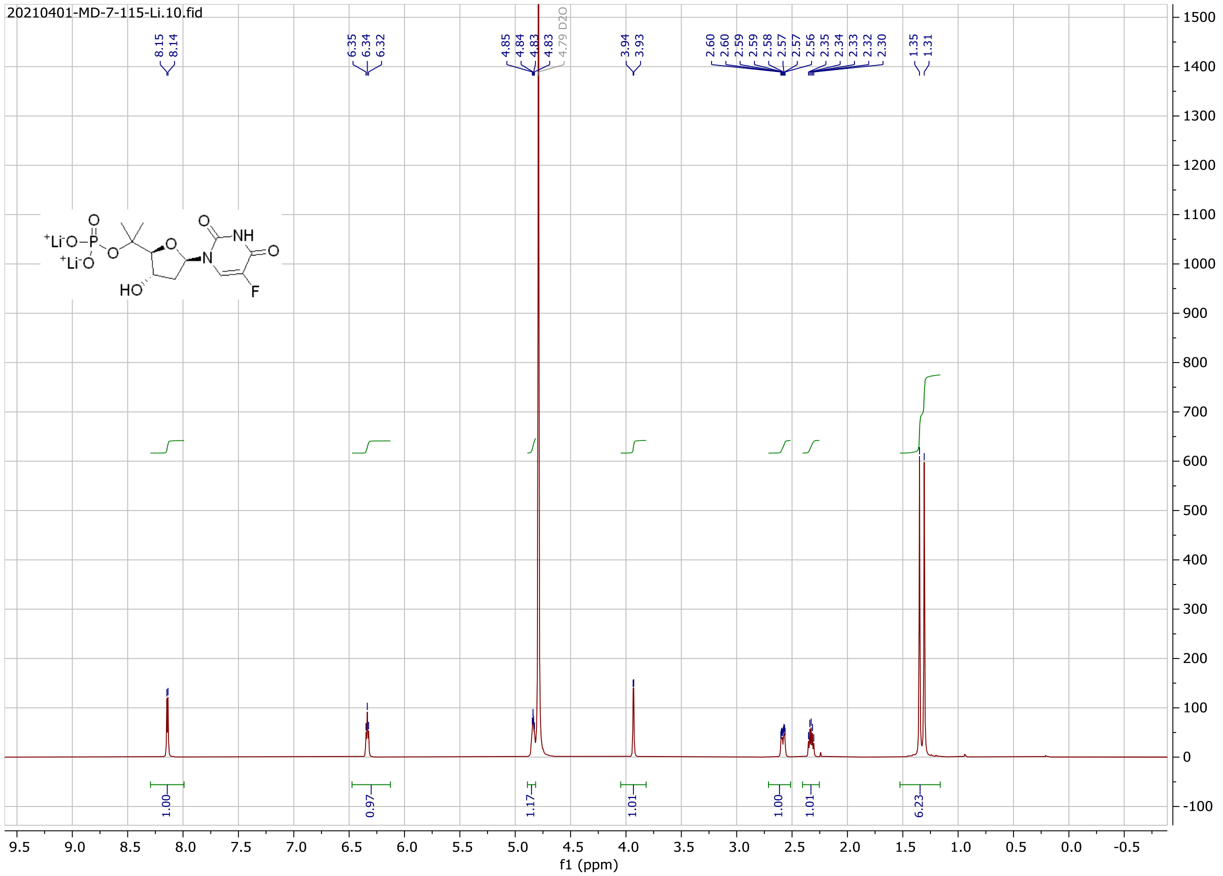
20210401-MD-7-115-Li.10.fid
8.15
8.14
6.35
6.34
6.32
4.85
4.84
4.83
4.83
4.79 D2O
3.94
3.93
2.60
2.60
2.59
2.59
2.58
2.57
2.57
2.56
2.35
2.34
2.33
2.32
2.30
1.35
1.31
1.00
0.97
1.17
1.01
1.00
1.01
6.23
f1 (ppm)

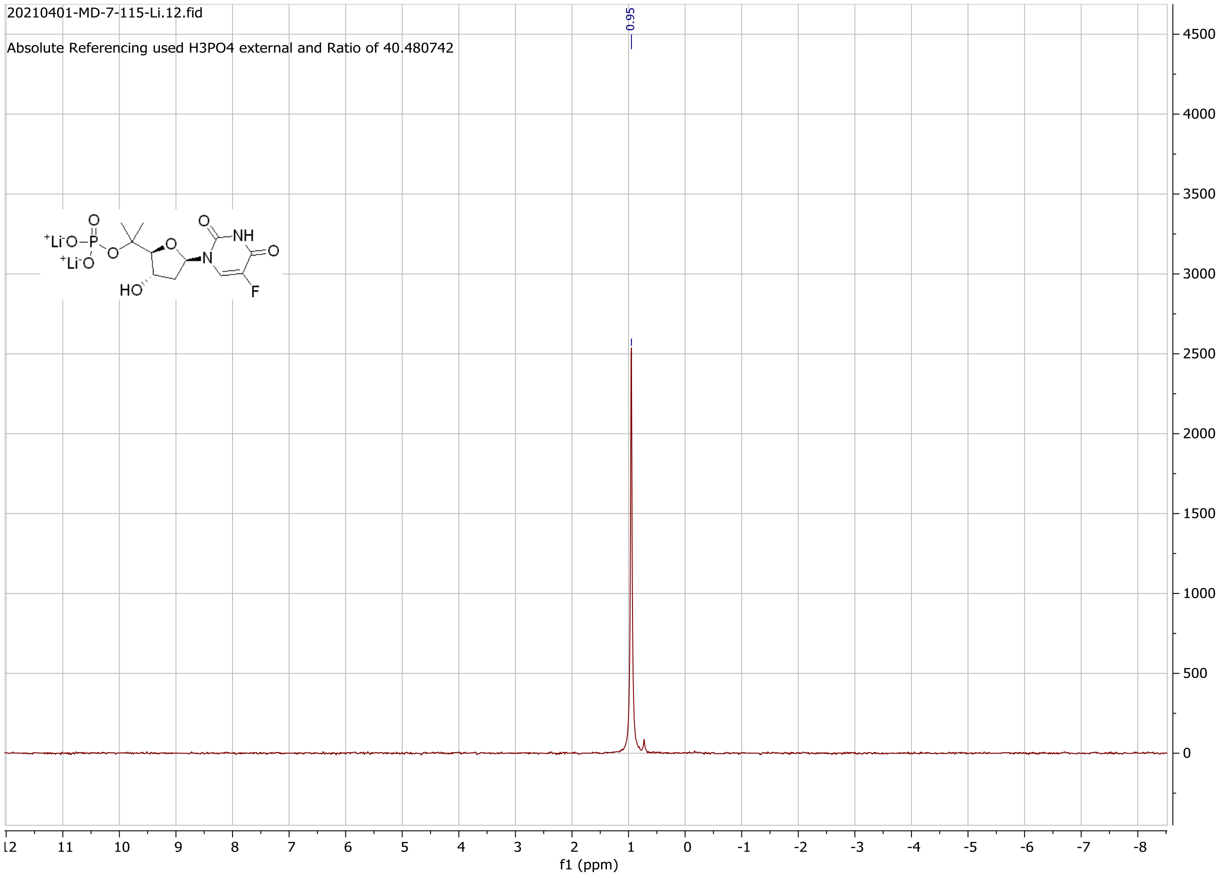

20210401-MD-7-115-Li.12.fid
Absolute Referencing used H3PO4 external and Ratio of 40.480742
0.95
f1 (ppm)

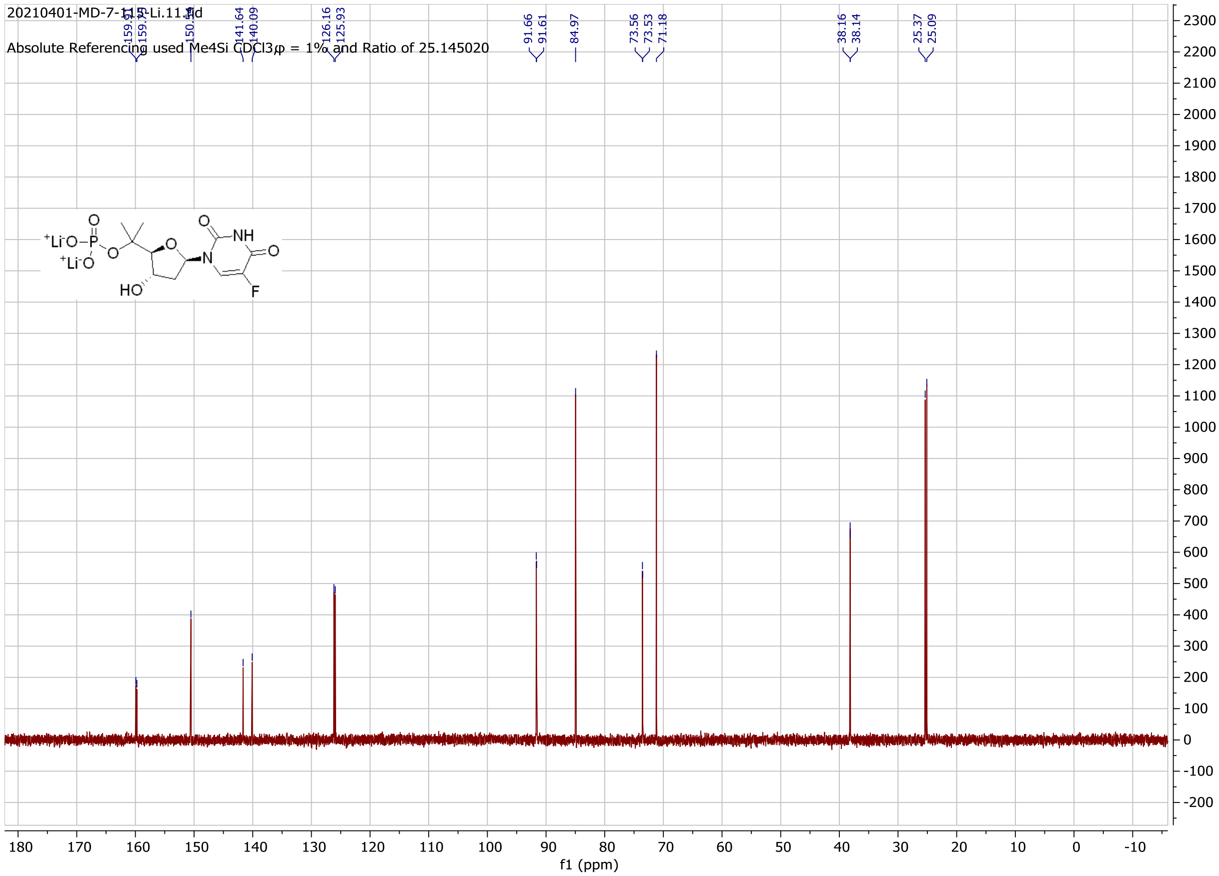
20210401-MD-7-115-Li.11.fid
Absolute Referencing used Me4Si CDCl3,φ = 1% and Ratio of 25.145020
159.79
150.54
141.64
140.09
126.16
125.93
91.66
91.61
84.97
73.56
73.53
71.18
38.16
38.14
25.37
25.09
+Li-O
+Li-O
P
O
O
O
HO
N
NH
O
O
F
f1 (ppm)
